# Acquired resistance to the RAS(ON) multi-selective inhibitor daraxonrasib guides rational combination therapy strategies in pancreatic cancer

**DOI:** 10.1038/s41591-026-04537-w

**Published:** 2026-08-11

**Authors:** Ida Aronchik, Sumit Kar, Yongxian Zhuang, Ethan Ahler, Lick Pui Lai, Vidya Seshadri, Yu Chi Yang, Ashenafi Bulle, Marie Menard, Biswadeep Nayak, Mark P. Labrecque, Julien Dilly, Eejung Kim, Lingyan Jiang, Jason Yano, Urszula N. Wasko, Ciara Helland, Sean Bredeson, Brett Garrick, Yevgeniy Gindin, Brad Sickler, Xing Wei, Kyle Seamon, Jingjing Jiang, Kian-Huat Lim, Matthew Holderfield, Elsa Quintana, Aparna Hegde, Zeena Salman, Alexander Starodub, Alexander Spira, Wungki Park, David S. Hong, Minal Barve, Meredith Pelster, David Sommerhalder, Salman R. Punekar, Ignacio Garrido-Laguna, Brian M. Wolpin, Anirban Maitra, W. Clay Gustafson, Steve Kelsey, Jacqueline A. M. Smith, Kevin K. Lin, Andrew J. Aguirre, Mallika Singh

**Affiliations:** 1 Revolution Medicines, Redwood City, CA USA; 2 Division of Oncology, Department of Internal Medicine, Washington University School of Medicine, St. Louis, MO USA; 3 Department of Medical Oncology, Dana-Farber Cancer Institute, Boston, MA USA; 4 Harvard Medical School, Boston, MA USA; 5 Broad Institute of MIT and Harvard, Cambridge, MA USA; 6 The Christ Hospital Health Network, Parkview Physician Group - Parkview Cancer Institute, Cincinnati, OH USA; 7 NEXT Oncology Virginia, Virginia Cancer Specialists Research Institute, Fairfax, VA USA; 8 Memorial Sloan Kettering Cancer Center, David M. Rubenstein Center for Pancreatic Cancer Research, Weill Cornell Medical College, New York, NY USA; 9 The University of Texas MD Anderson Cancer Center, Department of Investigational Cancer Therapeutics, Houston, TX USA; 10 University of Utah, Huntsman Cancer Institute, Salt Lake City, UT USA; 11 Mary Crowley Cancer Research, Texas Oncology, Dallas, TX USA; 12 Sarah Cannon Research Institute, Nashville, TN USA; 13 NEXT Oncology, San Antonio, TX USA; 14 NYU Langone Health, Perlmutter Cancer Center, New York, NY USA

**Keywords:** Cancer therapy, Cancer therapeutic resistance, Pancreatic cancer

## Abstract

Daraxonrasib is an orally bioavailable RAS(ON) multi-selective tri-complex inhibitor of the oncogenic mutant and wild-type variants of N, H and KRAS. We previously reported encouraging efficacy in a phase 1/2 clinical trial evaluating daraxonrasib monotherapy at clinically active dose levels in patients with previously treated, RAS mutant metastatic pancreatic adenocarcinoma (PDAC), providing the basis for confirmatory evaluation in the randomized phase 3 RASolute 302 clinical trial. Here we report mechanisms of acquired resistance to daraxonrasib monotherapy observed through targeted sequencing of over 800 genes in paired pretreatment and end of treatment circulating tumor DNA samples from 44 patients in the phase 1/2 clinical trial. Treatment-emergent genomic alterations in the RAS signaling pathway were observed in more than half (26 of 44; 59%) of these patients, including, most notably, mutant *KRAS* amplifications in one-third (16 of 44; 36%), as well as alterations in receptor tyrosine kinase (RTK) (4 of 44; 9%), MAPK (11 of 44; 25%) and PI3K (4 of 44; 9%) pathways. Notably, no acquired secondary *KRAS* mutations were observed, distinct from resistance profiles of mutant-selective KRAS G12C(OFF) inhibitors. To corroborate these clinical findings, we found, or mechanistically established, concordant mechanisms of daraxonrasib resistance in human and murine preclinical models of PDAC, including mutant *KRAS* and *MYC* amplification and RTK upregulation, with these alterations guiding various combination therapy concepts. Notably, daraxonrasib combined with agents targeting DNA damage response, RTKs or the mutant-selective RAS(ON) G12D inhibitor zoldonrasib averted resistance in preclinical models. Collectively, these results show that most daraxonrasib genomic resistance mechanisms drive reactivation of RAS pathway signaling and guide potential combination strategies in PDAC for further investigation.

## Abstract (web summary)

Circulating tumor DNA analyses in 44 patients with pancreatic cancer receiving the RAS(ON) multi-selective inhibitor daraxonrasib as part of a phase 1/2 trial, and preclinical translational modeling approaches, identify mechanisms of acquired resistance to monotherapy and inform potential combination strategies to overcome resistance.

## Main

Hotspot RAS mutations are among the most prevalent oncogenic drivers in cancer, including in PDAC, non-small cell lung cancer (NSCLC) and colorectal cancer (CRC). Mutated RAS is an oncogenic driver in over 90% of PDAC cases^1,2^. These hotspot mutations stabilize RAS in its active GTP-bound (‘ON’) state, driving increased RAS/MAPK signaling, proliferation, metastasis and immune evasion^3–5^. Therefore, broad-spectrum RAS(ON) inhibition holds the potential to benefit a large population of patients with cancers driven by activating RAS mutations^6,7^.

Daraxonrasib is a RAS(ON) multi-selective inhibitor that targets the active, GTP-bound forms of KRAS, HRAS and NRAS, with affinity for both mutant and wild-type (WT) variants^8,9^. Daraxonrasib is currently being investigated in multiple clinical trials, including an ongoing phase 1/2 monotherapy study in patients with previously treated advanced RAS mutant tumors (NCT05379985)^10,11^. We previously reported the antitumor activity of daraxonrasib in this single-arm study in patients with metastatic PDAC receiving daraxonrasib as second-line treatment at 300 mg daily^12^). In the second-line RAS mutant population (*n* = 38), we observed a median progression-free survival (PFS) of 8.1 months (95% CI 5.9–10.1) and a median overall survival of 15.6 months (95% CI 10.9–not evaluable) with a median follow-up of 28 months (range 17.1–32.6)^12^. We recently reported confirmatory results in this second-line population in the randomized phase 3 RASolute 302 clinical trial^13^.

While the clinical activity of daraxonrasib monotherapy is encouraging, many patients eventually experience disease progression indicating the development of acquired resistance on treatment. Mechanisms of resistance to RAS(ON) inhibitors in PDAC have not been elucidated and may be distinct from those for daraxonrasib in other solid tumors^14^ given the exquisite RAS-dependency of this disease^15,16^. Mechanisms of acquired resistance to KRAS G12C(OFF) inhibitors have been described in NSCLC, CRC and, to a more limited degree, in PDAC, which include secondary oncogenic *KRAS* mutations and mutant *KRAS* allele amplification (*KRAS*^*amp*^)^17,18^. Identifying treatment-emergent resistance mechanisms has the potential to guide rational combination therapies. This study presents the first insights into the genomic mechanisms of acquired resistance to daraxonrasib monotherapy in PDAC and identifies potential combination therapy strategies to overcome resistance.

## Results

### Acquired genomic alterations in patients with metastatic PDAC following progression on daraxonrasib monotherapy

**Extended Data Fig. 1:**
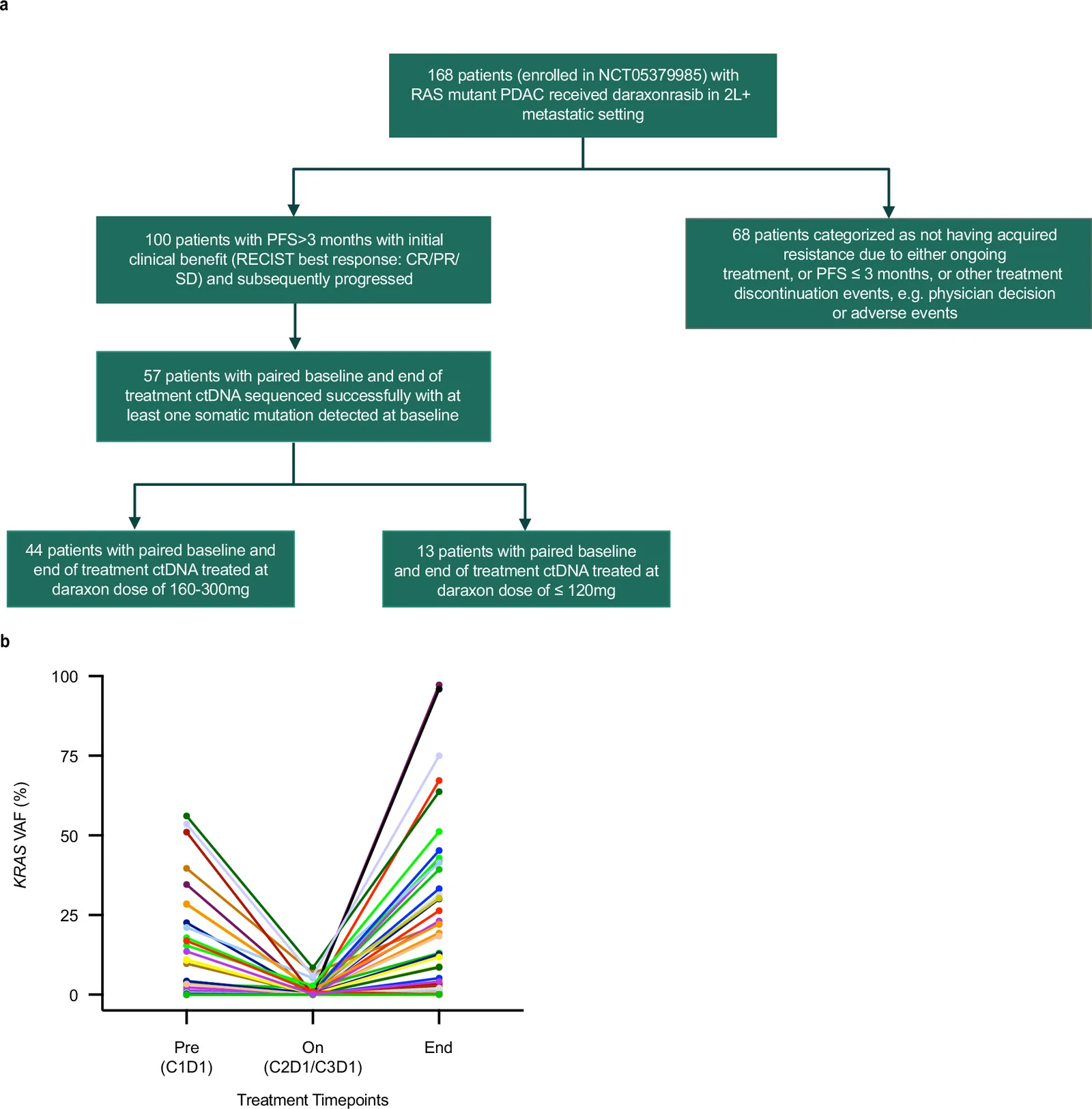
Patient cohort for acquired resistance. **a**, CONSORT diagram outlining selection of patients with PDAC with acquired resistance to daraxonrasib in the Phase 1/2 Study (NCT05379985) and ctDNA profiling. **b**, Changes in ctDNA *KRAS* VAF detected at pretreatment (Pre), early on treatment (either cycle 2 day 1 or cycle 3 day 1; On), and EOT (End) demonstrate clinical benefit from daraxonrasib followed by acquired resistance in this patient population. Source data

We profiled paired circulating tumor DNA (ctDNA) samples (pretreatment and end of treatment (EOT) at the time of progression) from 44 patients with RAS mutant metastatic PDAC treated at a dose range of 160–300 mg daraxonrasib daily as second or later line therapy in a single-arm phase 1/2 trial (NCT05379985)^12^. Previously reported translational modeling predicted this dose range drives RAS pathway inhibition ≥90% and antitumor activity in humans^8^. Each patient exhibited either a complete response (CR), partial response (PR) or stable disease (SD) on treatment with a PFS of >3 months followed by subsequent progression on therapy (Extended Data Fig. 1a). Consistent with daraxonrasib activity, each patient showed a relative reduction in mutant *KRAS* variant allele frequency (VAF) in ctDNA from pretreatment time point (cycle 1, day 1; C1D1) to early on treatment (cycle 2, day 1 (C2D1) or cycle 3, day 1 (C3D1)). We subsequently observed an increase in mutant *KRAS* VAF at EOT in most patients (Extended Data Fig. 1b).

**Fig. 1:**
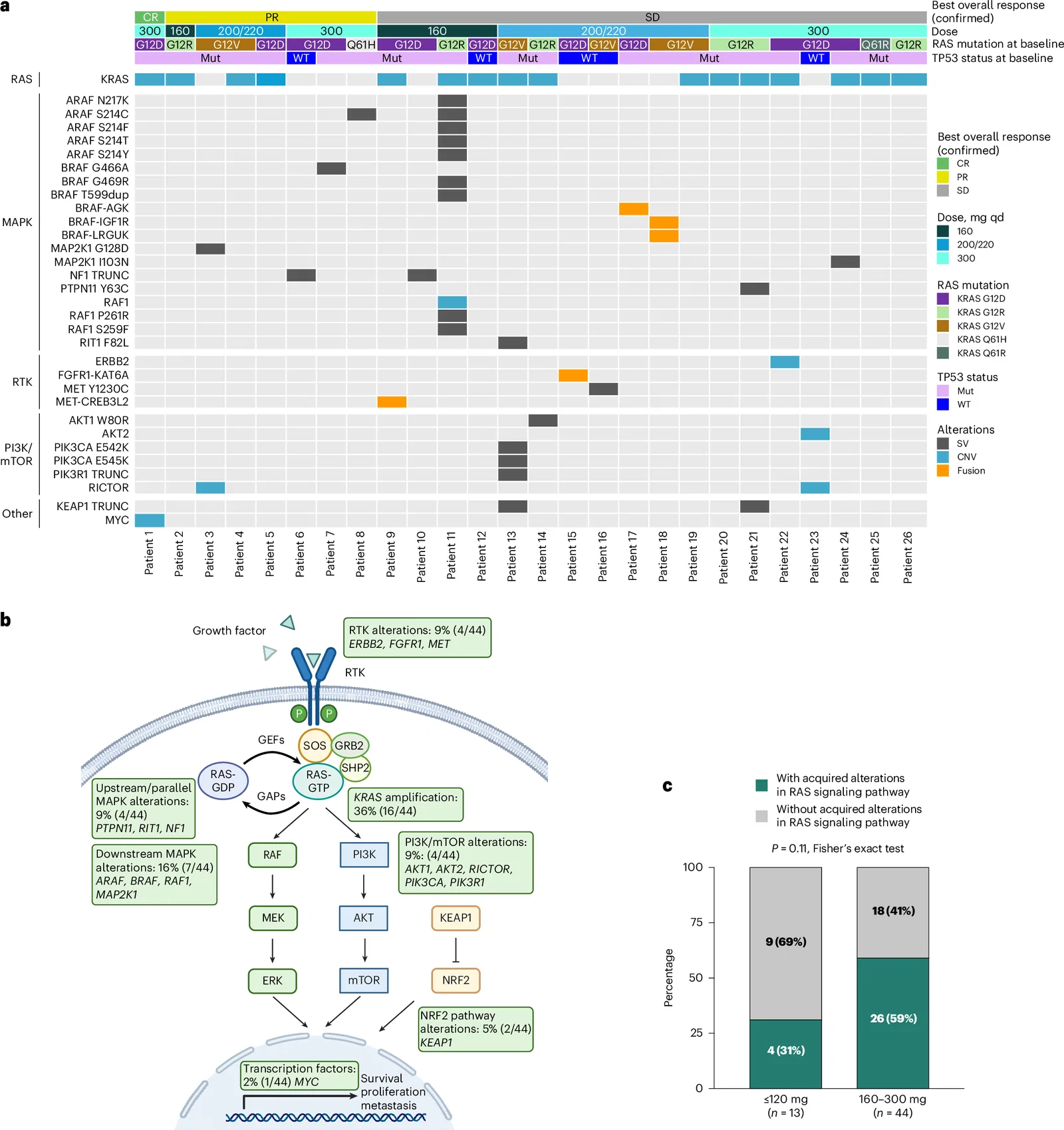
Putative acquired genomic resistance mechanisms to daraxonrasib treatment in patients with PDAC. **a**, Putative acquired genomic resistance mechanisms were identified in a majority of the 44 patients profiled. Each column represents a single patient, and each row represents a specific genomic alteration. First four rows depict confirmed best overall response by RECIST v.1.1, planned dose, RAS mutation at enrollment and pretreatment *TP53* alteration status (based on pretreatment ctDNA). Supplementary Table 3 lists the *KRAS* CN for each patient. Gene names not italicized per standard oncoprint conventions. **b**, Summary of putative acquired genomic resistance mechanisms (RAS, MAPK, RTK, PI3K/mTOR and other (*KEAP1* and *MYC*) pathways) in patients with PDAC. Eleven patients acquired more than one alteration in this pathway. **c**, Association of dose level of daraxonrasib (*x* axis) with acquisition of RAS signaling pathway genomic alterations (all genes shown in the oncoprint) at progression. *P* = 0.11, two-sided Fisher’s exact test. SV, short variant, defined as single-nucleotide variant (SNV) or short indel; CNV, copy-number variant, defined as ‘focal amplification’ (for oncogenes) or ‘homozygous deletion’ (for tumor-suppressor genes); copy number (CN) above 2 is defined as amplification; Fusion, requires at least one of the partner genes to be in pathway of interest; TRUNC, variant resulting in predicted truncated protein, defined as nonsense mutation, splice site or indel; qd, once daily. Panel **b** created in BioRender; Nayak, B. https://BioRender.com/23cpkzi (2026). Source data

We detected acquired oncogenic alterations in 26 of 44 (59%) patients, including several putative genomic resistance mechanisms resulting in aberrant RAS signaling: alterations in RAS, MAPK, PI3K and RTK components (Fig. 1a and Supplementary Table 1). Among the acquired oncogenic alterations in RAS pathway signaling genes, *KRAS*^*amp*^ was the most frequent, identified in 36% (16 of 44) of the patient samples profiled, accounting for 62% (16 of 26) of all putative RAS pathway genomic resistance mechanisms. Of note, we did not observe acquired secondary mutations in *KRAS*, in contrast to previous observations of these as resistance drivers to mutant-selective KRAS G12C(OFF) inhibition^19,20^.

We detected multiple acquired alterations in ctDNA from 11 of 44 patients (25%). Putative genetic mechanisms of acquired resistance are summarized in the context of the RAS signaling pathway in Fig. 1b. We documented acquired alterations in RAF genes (5 of 44; 11%), including multiple single-nucleotide *ARAF*, *BRAF* and *RAF1* variants along with co-occurring *KRAS*^*amp*^ in one patient (patient 11; Fig. 1a). RTK alterations (4 of 44; 9%), included HER2 (*ERBB2*) amplification (copy number of 2.31), a gene fusion in *MET*, each co-occurring with mutant *KRAS*^*amp*^; a *FGFR1* gene fusion, and a single-nucleotide variation in *MET* observed in two other patients. PI3K/mTOR signaling pathway genes (*PIK3CA*, *AKT1*, *AKT2*, *RICTOR* and *PIK3R1*) were altered in a minority of patient samples (4 of 44; 9%) with only an *AKT2* amplification found without co-occurring *KRAS*^*amp*^. We also observed truncating mutations in *NF1* (2 of 44; 4%), *KEAP1* (2 of 44; 4%), a *PTPN11* mutation or *MYC* amplification in one patient each (1 of 44; 2%).

Of 18 of 44 patients with no putative genomic resistance mechanism identified in ctDNA, 5 had no detectable acquired alterations in any of the 800+ genes examined (Supplementary Table 1). The remaining 13 of 18 patients acquired one or more alterations in genes either associated with clonal hematopoiesis of indeterminate potential, such as *TP53, ASXL1, PPM1D* and *TET2* (refs. ^21–23^) or non-recurrent oncogenic alterations in tumor-suppressor genes not detected in pretreatment ctDNA, potentially due to low shedding (*CDKN2A, SMAD4, ARID1A* and *BRCA1*; Supplementary Table 4). Additionally, 2 of these 18 patients had pre-existing *TP53* alterations and acquired additional loss of function mutations in this gene.

Some patients were treated at doses lower than 160 mg in the dose-escalation phase of the aforementioned phase 1/2 trial^12^ (Extended Data Fig. 1). We found that acquired RAS pathway alterations were more frequent at the higher dose range of 160–300 mg daily than in ctDNA of 13 patients treated with daraxonrasib ≤120 mg daily (59% versus 31%), although this difference did not reach statistical significance (*P* = 0.11, two-sided Fisher’s exact test; Fig. 1c). Of note, the types of acquired alterations seen in 4 of 13 patients at lower doses were consistent with those seen in the higher dose range (*KRAS*^*amp*^, and SNVs in *BRAF*, *KEAP1*, *PTPN11*, *NF1*, *RIT1* and *PIK3CA*; Supplementary Table 2).

### Acquired mutant *KRAS*^*amp*^ as a resistance mechanism

We further examined genomic features in ctDNA from 16 patients with acquired *KRAS*^*amp*^, the most frequently observed resistance mechanism (Fig. 1a and Supplementary Table 3). While the acquired *KRAS*^*amp*^ copy number observed at progression showed a wide range from 2.21 to 28.36 copies, we found no relationship between this copy number and daraxonrasib dose or baseline *KRAS* mutant allele (*KRAS* G12D, *KRAS* G12V, *KRAS* G12R and *KRAS* Q61H).

**Fig. 2:**
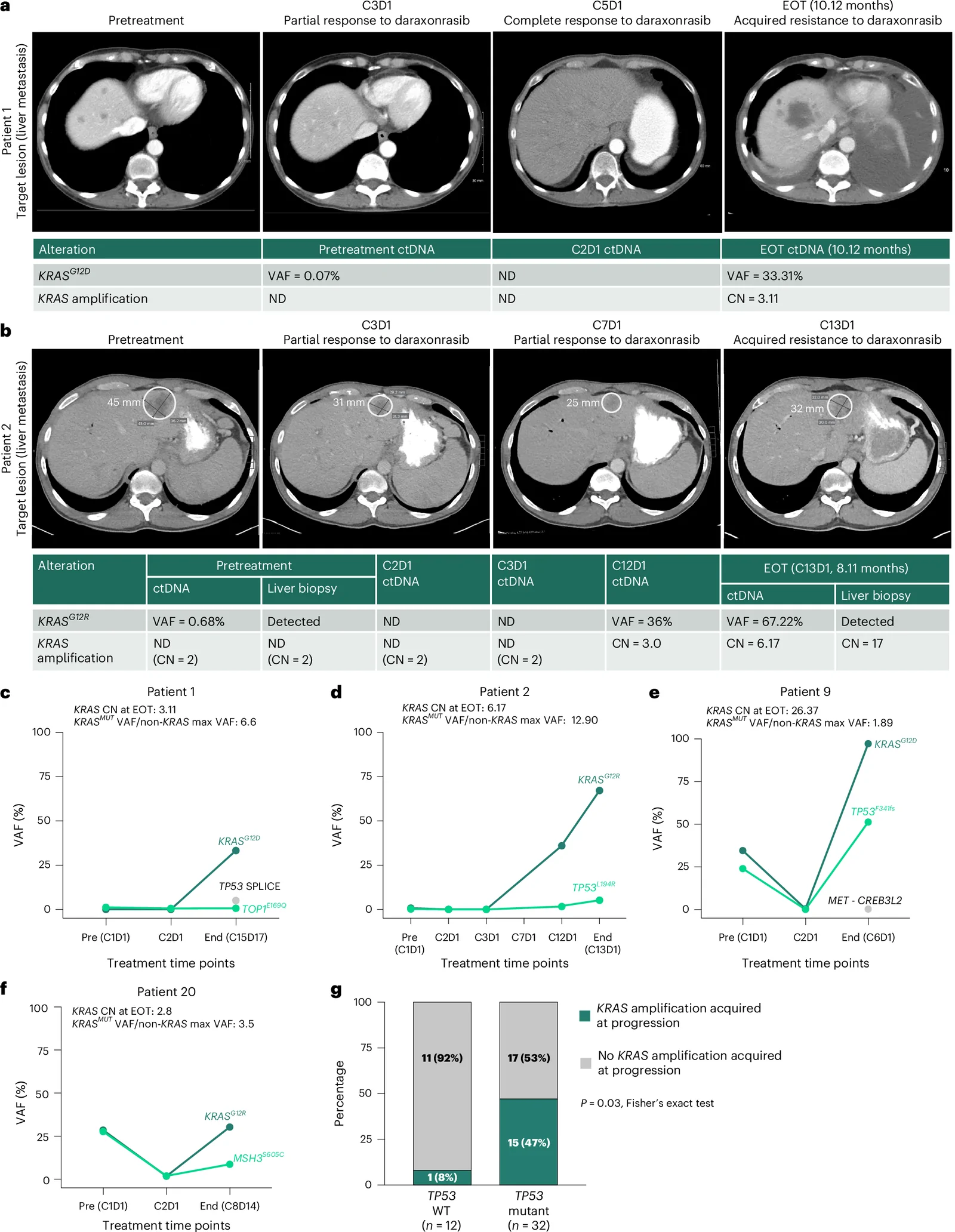
*KRAS*^*amp*^ is a recurrent mechanism of acquired genomic resistance to daraxonrasib in patients with PDAC. **a**,**b**, Computed tomography scans for two patients who had complete (**a**) or partial responses (**b**) to daraxonrasib (21-day cycles) followed by disease progression. ctDNA profiling in both patients showed complete clearance of *KRAS*^*G12D*^ or *KRAS*^*G12R*^ early on-treatment preceding the clinical response followed by a large increase in *KRAS*^*G12D*^ or *KRAS*^*G12R*^ VAF at progression (EOT) and newly acquired *KRAS* amplification (**a**,**b**, bottom tables). Additionally, patient 1 was also found to have acquired *MYC* amplification as detected in the EOT ctDNA. In patient 2, a paired pretreatment and EOT liver biopsy was sequenced with comprehensive genomic profiling which confirmed the acquired *KRAS* amplification at progression detected in ctDNA. The patient also had an oncogenic *TP53* L194R alteration before therapy detected in pretreatment ctDNA and liver biopsy. **c**–**f**, Representative ctDNA dynamics in individual patients with varying acquired *KRAS* amplification copy numbers at EOT. VAF at pretreatment (Pre), early on-treatment at cycle 2 day 1 (On), and EOT at progression (End) with daraxonrasib. RAS mutant allele represented in dark green and the highest somatic mutation in a non-RAS gene shown in light green. Other somatic oncogenic alterations are shown in gray. In each patient, *KRAS* amplification was not detected at Pre and On time points (CN = 2) and was detected at EOT at the CN shown. **g**, Association of acquired *KRAS* amplification at progression (*x* axis) with pretreatment *TP53* oncogenic mutation. *P* = 0.03, two-sided Fisher’s exact test. One patient with acquired *KRAS* amplification at progression did not have a pretreatment *TP53* alteration detected in pretreatment ctDNA (low shedding with max VAF = 0.59%); however, a baseline *TP53* alteration was detected in archival tumor tissue testing (13.5% VAF). ctDNA dynamics for all patients with *KRAS* amplification are shown in Supplementary Tables 1 and 3. ND, not detected. Source data

**Extended Data Fig. 2:**
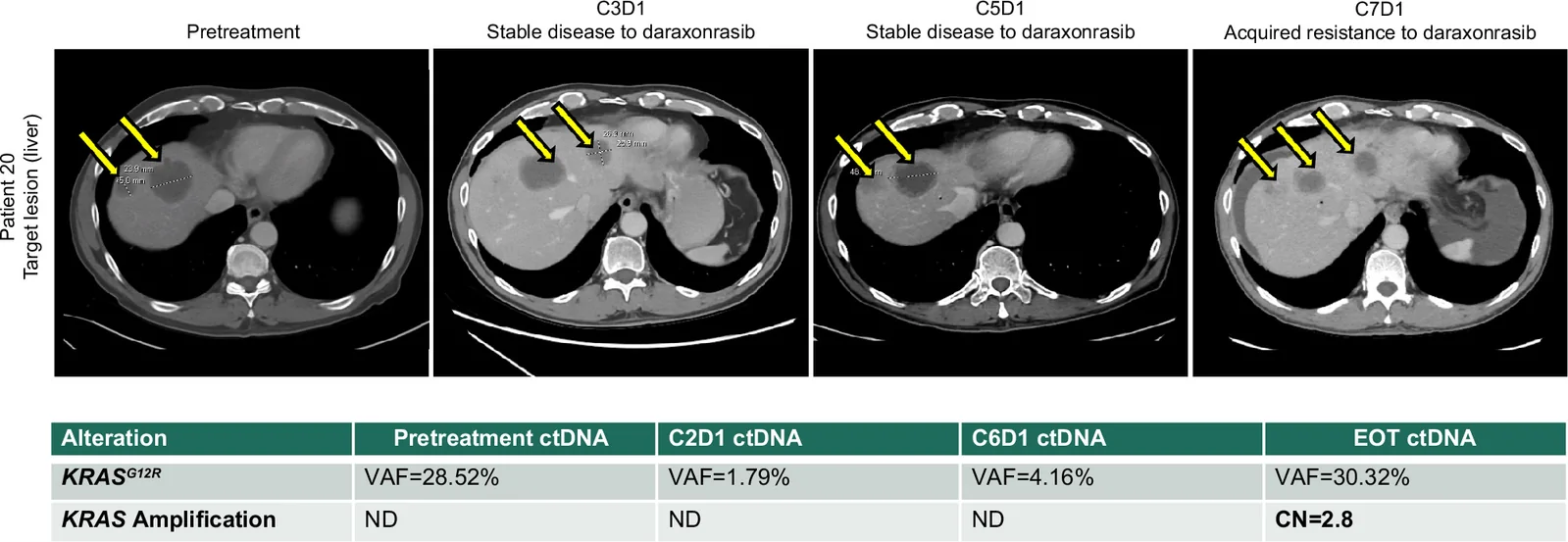
Additional patient case study of KRAS amplification. Computed tomography scans for a patient who had stable disease to daraxonrasib (21-day cycles) followed by disease progression at 4.11 months (Panel A). ctDNA profiling in this patient showed reduction of *KRAS*^*G12R*^ early on-treatment followed by a large increase in *KRAS*^*G12R*^ VAF at C6D1 and progression (EOT) and newly acquired *KRAS* amplification (bottom table). ND = not detected. A *KRAS* ddPCR assay at C6D1 prior to progression did not detect acquired *KRAS*^*Amp*^, which was later detected by NGS at progression at a copy number (CN) of 2.8 along with new metastatic lesions, but demonstrated an increase in *KRAS*^*G12R*^ VAF. Source data

Next, we dissected the dynamics of *KRAS*^*amp*^ acquisition in ctDNA from four patients, one who experienced a CR, and three with PR or SD, followed by progression on treatment (Fig. 2 and Extended Data Fig. 2). Each patient had a baseline *KRAS* copy number of 2 in pretreatment ctDNA, and as noted above, showed a relative reduction in mutant *KRAS* VAF in ctDNA at C2D1. We then observed a marked increase in mutant *KRAS* VAF at progression in conjunction with detection of *KRAS*^*amp*^ in ctDNA. In patient 2, we confirmed acquired *KRAS*^*amp*^ in EOT ctDNA via next-generation sequencing from a tumor biopsy collected at progression (Fig. 2b). In this patient, we detected *KRAS*^*amp*^ in ctDNA one cycle immediately before (C12D1) radiological tumor progression at C13, consistent with *KRAS*^*amp*^ as a putative resistance mechanism. EOT ctDNA from patient 1 showed acquisition of *MYC*^*amp*^ as an additional putative resistance mechanism. Using a previously reported method to infer whether amplification observed in ctDNA occurred in the mutant or WT *KRAS* allele^24^, we calculated the ratio of mutant *KRAS* VAF relative to non-RAS tumor mutation VAFs (*KRAS*^MUT^ VAF/non-*KRAS* max VAF). We observed a relative ratio increase of >1 for mutant *KRAS* VAF relative to other somatic mutations in all 16 patients with *KRAS*^*amp*^ at progression, indicating that the mutant *KRAS* allele present before therapy is amplified in each case (Supplementary Table 3 and Fig. 2c–f).

Finally, we evaluated the association of mutant *KRAS*^*amp*^ at acquired resistance with common genomic co-alterations occurring at > 10% frequency in ctDNA from the 44 patients profiled herein. *TP53* mutations were the most common co-alterations found (Fig. 2g; present in 31 of 44; 70%), consistent with the expected frequency in *KRAS* mutant PDAC^1,2^. Furthermore, we observed a significant association between a pretreatment *TP53* alteration and acquired *KRAS*^*amp*^ at progression: 15 of 32 (47%) patients with pretreatment oncogenic *TP53* mutation in ctDNA showed acquired *KRAS*^*amp*^ at progression in comparison to 1/12 (8%) patients with pretreatment WT *TP53* (*P* = 0.03, two-sided Fisher’s exact test). We did not find any significant associations between the acquisition of *KRAS*^*amp*^ at progression and pretreatment co-alterations in *SMAD4, CDKN2A*, *ARID1A* and *CHEK2* (Supplementary Table 4), with each of these occurring at frequencies near the expected prevalence in PDAC^1^.

### Absence of acquired secondary‑site mutations in *KRAS*

**Extended Data Fig. 3:**
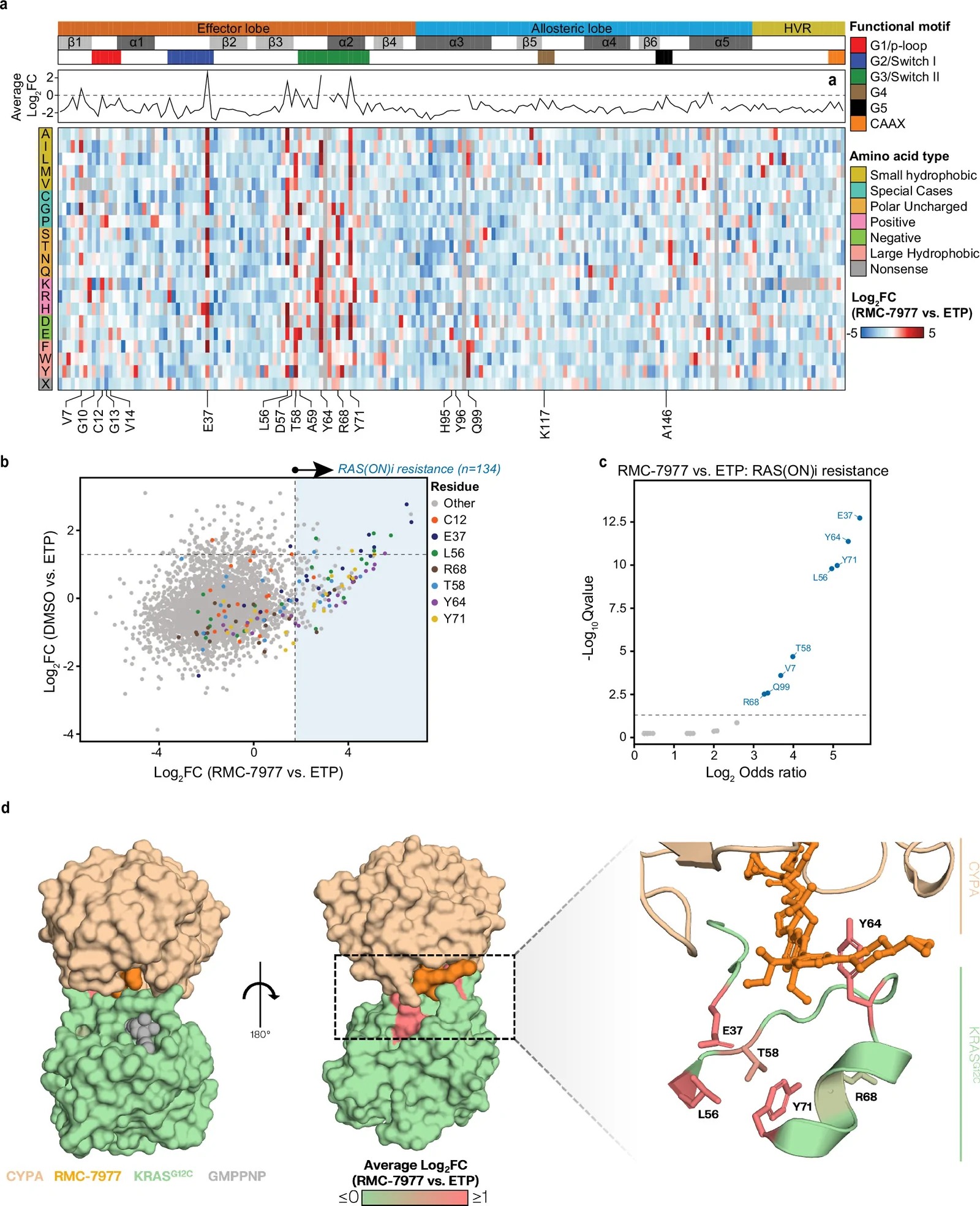
Deep mutational scanning screen to identify second-site mutations in *KRAS* that confer resistance to RAS(ON) multiselective tri-complex inhibitors. **a**, Heatmap depicting the enrichment (red) and depletion (blue) values as log_2_ fold change (Log_2_FC) for each allele from *KRAS*^*G12C*^ deep mutational scanning (DMS) gain-of-function screen in Ba/F3 cells, comparing day 6 with RMC-7977 treatment and day 0 (ETP). Each column represents an amino acid in KRAS^G12C^, and each row a substituted residue grouped by physicochemical properties. The secondary structures, binding motifs (G1-G5), and the two Switch motifs are annotated on top. The average enrichment value for each residue in the DMS screen is displayed in the trace plot above. Gray cells indicate alleles missing from the library. **b**, Scatter-plot depicting the allelic Log_2_FC between 6-day RMC-7977 (x-axis) or 6-day DMSO (y-axis) compared to day 0 (ETP). Thresholds represent the library’s average Log_2_FC + 2*S.D for each comparison and capture therapeutic resistance (x-axis) or growth advantage (y-axis). A subset of alleles is colored according to their location in the KRAS^G12C^ protein. **c**, Volcano plot depicting the residue-wise enrichment of RAS(ON)inhibitor resistance-inducing residues as identified in panel b. Odds-ratios and Adjusted P-values determined via Fisher’s Exact test and adjusted with Benjamini-Hochberg correction. **d**, left panel. Mapping of the DMS enrichment results onto the GMPPNP:KRAS^G12C^:RMC-7977:CYPA protein structure complex (PDB: 8BTK). The coloration represents the residue-level average Log_2_FC from the day 6 RMC-7977-treated arm compared to day 0 ETP. right panel. Zoom-in on the binding interface of the tri-complex inhibitor with KRAS^G12C^ (green) and CYPA (beige) showing KRAS^G12C^ residues involved in the binding to either RMC-7977 and/or CYPA. Source data

No secondary gene mutations were detected in ctDNA from the 44 patients with PDAC profiled in this study. Of note, Sang et al. recently demonstrated that daraxonrasib treatment-emergent mutations at *KRAS* Y64 in non-PDAC patients disrupt the affinity of tri-complex formation^14^. In parallel, we performed a deep mutational scanning (DMS) screen in the Ba/F3 mouse pro-B cell line to evaluate such possible second-site mutations in *KRAS* that confer resistance to daraxonrasib. This screen identified mutations at multiple *KRAS* residues that conferred resistance, including at E37, Y64, Y71, L56, T58 and R68, among others; many of these mutations were also identified via base-editing screens in mutant *Kras*^25^. Analysis of each of these second-site KRAS alterations in the X-ray crystal structure of the tri-complex containing daraxonrasib bound to KRAS G12C mutant protein and cyclophilin A (CYPA) revealed that most of these changes reside at the inhibitor-binding interface and are predicted to attenuate tri-complex formation by daraxonrasib^14^ (Extended Data Fig. 3). Daraxonrasib treatment-emergent mutations at Y64 disrupt RAS binding, whereas changes at Y71 enhance RAF-binding as potential resistance mechanisms^14^.

Consistent with Sang et al., showing that *BRAF* mutations can confer resistance to RAS(ON) multi-selective inhibition^14^, the observation of RAF gene alterations in PDAC in this study suggests that these acquired mutations may attenuate the activity of daraxonrasib and reactivate downstream RAS pathway signaling. Consistent with the clinical observations described herein, we did not identify second-site mutations in *KRAS* in any preclinical PDAC model with acquired resistance to long-term RAS(ON) inhibitor treatment (in vitro or in vivo, see below).

### Modeling acquired resistance to daraxonrasib monotherapy

**Fig. 3:**
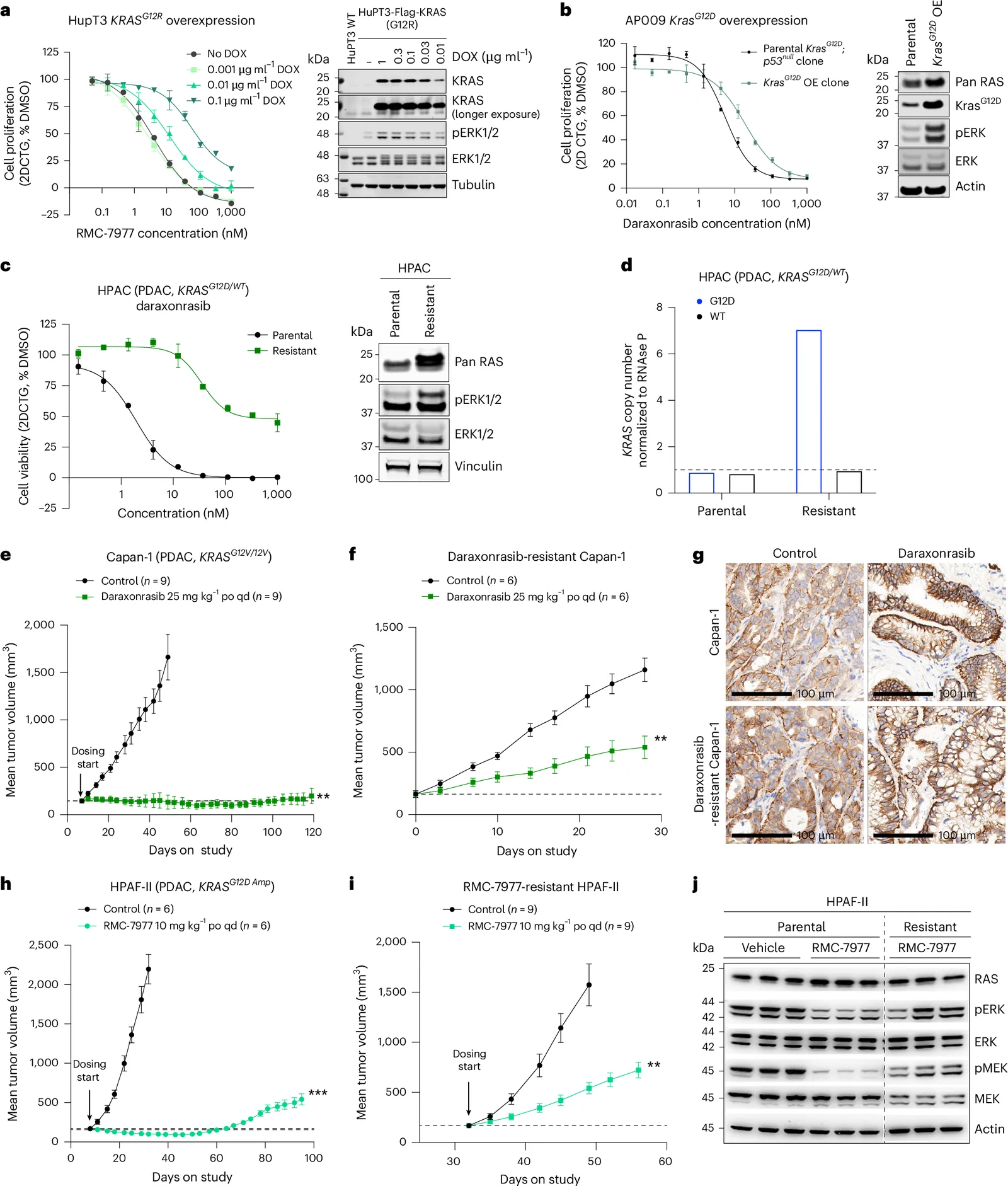
Acquired resistance to daraxonrasib monotherapy in preclinical models. **a**, Five-day two-dimensional (2D) viability assays were performed using HuP-T3 cells with doxycycline (DOX)-inducible *KRAS*^*G12R*^ overexpression. One of two independent experiments is shown; technical duplicates were included. Data represent mean ± s.d. **b**, Five-day 2D viability assays were performed in HuP-T3 cells with DOX-inducible *KRAS*^*G12R*^ expression and AP009 murine pancreatic organoids engineered to overexpress *Kras*^*G12D*^. RMC-7977 dose–response curves and immunoblots of KRAS, pERK and ERK following KRAS induction are shown. **c**, HPAC xenograft tumors that regrew after initial response to the RAS(ON) G12D-selective inhibitor RMC-9945 were used to generate resistant cell lines. Sensitivity to daraxonrasib was assessed by 5-day 2D viability assays in parental and resistant HPAC cells. One of two independent experiments is shown; technical duplicates were included, and data represent mean ± s.d. Immunoblot analysis of pERK, ERK and RAS is shown. **d**, Digital PCR was used to quantify *KRAS*^*G12D*^ and WT *KRAS* CN in parental and resistant HPAC cells. Relative CN was normalized to RNase P (mean ± s.e.m., *n* = 2). **e**,**f**, Mice bearing parental (**e**) or daraxonrasib-resistant Capan-1 (**f**) CDX tumors were treated with vehicle or daraxonrasib at 25 mg kg^−1^ orally, once daily (po qd). Tumor volumes were assessed throughout the treatment period (mean ± s.e.m.). Vehicle control and treatment groups were compared by two-way repeated-measures analysis of variance (ANOVA) on the last measurement day of the control group (**P < 0.01). **g**, Representative images of immunohistochemistry (IHC) staining of HER2 in Capan-1 parental and daraxonrasib-resistant tumors collected at 4 h post-dosing of vehicle control or daraxonrasib on day 7. **h**,**i**, Mice bearing parental (**h**) and resistant HPAF-II (**i**) CDX tumors were treated with vehicle control or RMC-7977 at 10 mg kg^−1^ po qd. Tumor volumes were monitored throughout treatment (mean ± s.e.m.) and compared by two-way repeated-measures ANOVA (**P < 0.01; ***P < 0.001). **j**, HPAF-II parental tumors treated with vehicle control or RMC-7977 and RMC-7977-resistant HPAF-II tumors were collected for immunoblot analysis of the indicated markers. DMSO, dimethylsulfoxide. Source data

**Extended Data Fig. 4:**
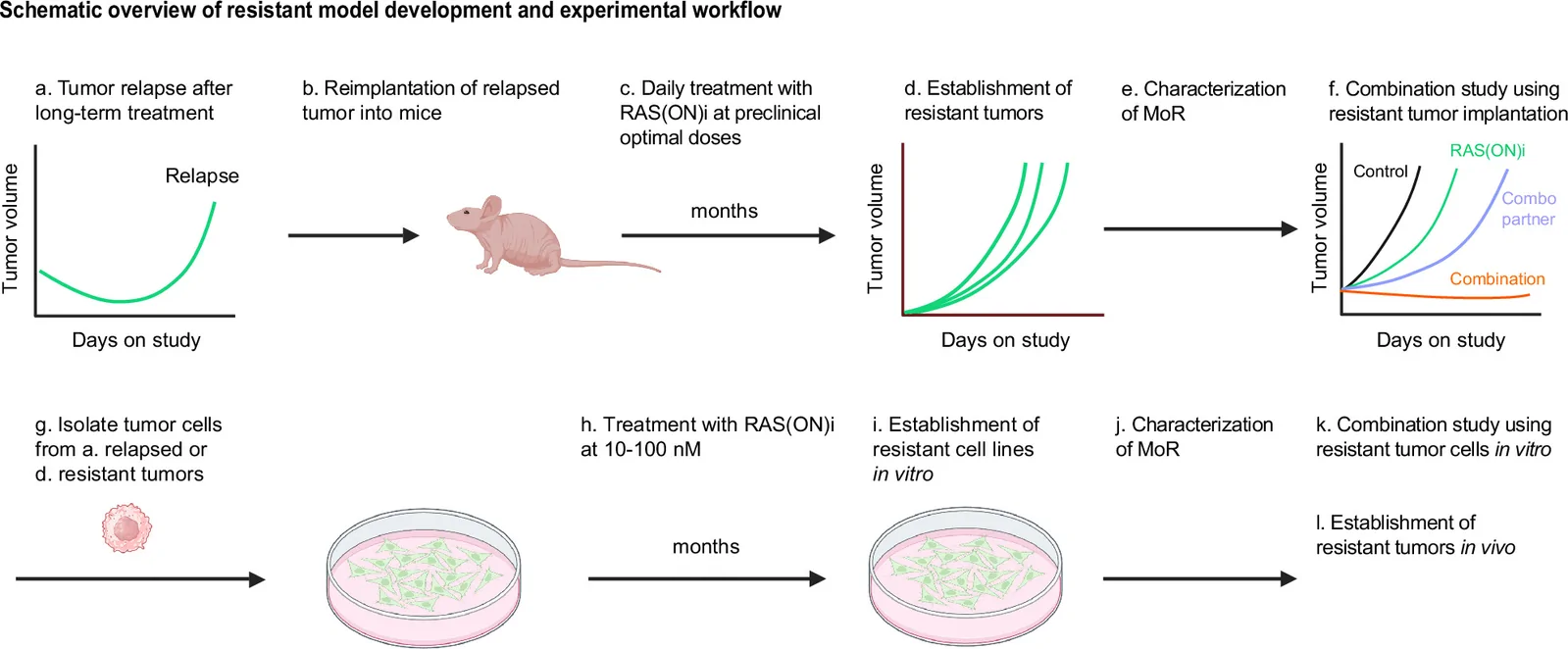
Schematic overview of resistant model development and experimental workflow. **a**, Long-term treatment of tumor-bearing mice with RAS(ON)i leads to initial tumor regression followed by relapse, as monitored by tumor volume over time. **b**, Relapsed tumors are harvested and reimplanted into naïve mice. **c**, Mice bearing reimplanted tumors are treated daily with RAS(ON)i at preclinical optimal doses. **d**, Continued treatment results in the establishment of tumors with acquired resistance to RAS(ON)i. **e**, Resistant tumors are characterized to define mechanisms of resistance (MoR). **f**, Combination therapy studies are performed using resistant tumor implants in vivo. **g**, Tumor cells are isolated from either relapsed tumors or resistant tumors. **h**, Isolated tumor cells are treated in vitro with RAS(ON)i at concentrations ranging from 10–100 nM. **i**, Resistant cell lines are established in vitro. **j**, In vitro resistant cell lines are characterized to identify MoR. **k**, Combination therapy studies are conducted using resistant tumor cells in vitro. **l**, Resistant cell lines are used to establish additional resistant tumors in vivo for further therapeutic evaluation.

**Extended Data Fig. 5:**
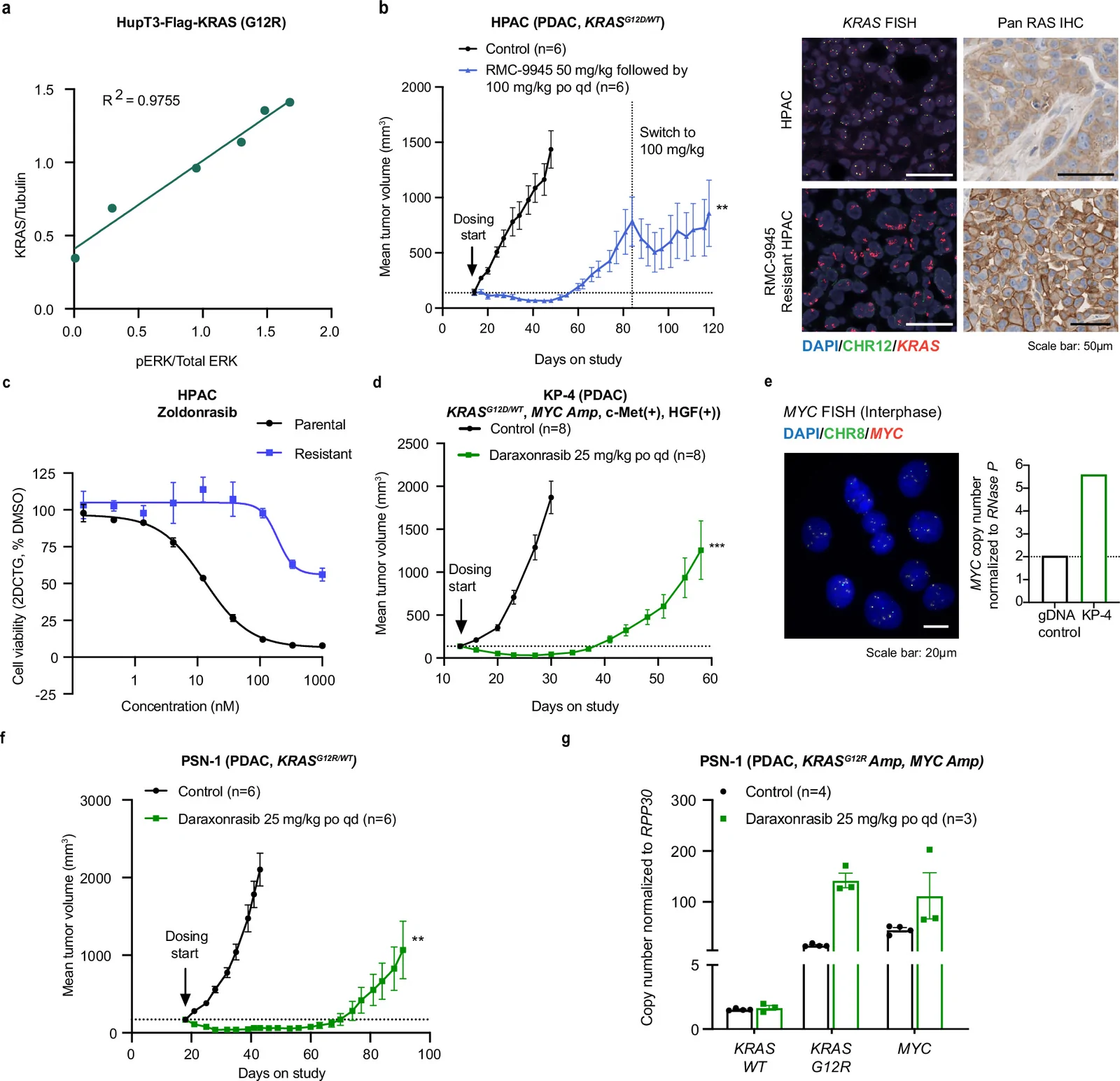
Characterization of Additional Preclinical Resistance Models. **a**, Densitometric quantification of Western blot analyses showing the relationship between KRAS protein abundance (normalized to αtubulin) and downstream RAS/MAPK pathway activation, measured as phosphoERK relative to total ERK, in HuPT3FlagKRAS(G12R) cells. Each data point represents an independent condition with increasing KRAS expression. Linear regression analysis demonstrates a strong positive correlation between KRAS levels and ERK pathway activation (R^2^ = 0.9755), consistent with dose dependent increases in oncogenic RAS signaling. **b, left panel** Mice bearing HPAC parental model xenografts were orally administered with vehicle control, RMC-9945 at 50 mg kg^−1^ po qd followed by 100 mg kg^−1^ po qd and tumor volumes were assessed throughout the treatment period (mean ± SEM). Vehicle control and treatment group were compared by two-way repeated-measures ANOVA on the last measurement day of the control group (**, P < 0.01). **Right panel**, Representative DNA FISH and pan RAS IHC images of end of study HPAC tumors from the left panel. Dual color FISH probes to *KRAS* (red) and chromosome 12 centromere (green) were used, and nuclei were co-stained with DAPI. **c**, Relapsed tumors were collected for in vitro resistant cell line development. 5-day 2D cellular viability assay was performed to test the sensitivity to zoldonrasib in parental and resistant cells. Dose response curves of cells treated with the indicated concentrations of zoldonrasib from one of two independent experiments are shown. Technical duplicates were included in each experiment, and data represent means ± standard deviations. **d**, Mice bearing KP-4 tumors (n = 8) were orally administered with vehicle control or daraxonrasib at 25 mg kg^−1^ po qd and tumor volumes were assessed through the treatment period (mean ± SEM). Vehicle control and treatment group were compared by two-way repeated-measures ANOVA on the last measurement day of the control group (***, P < 0.001). **e**, dPCR was performed to examine *MYC* copy number in the KP-4 cell line. Relative copy number of *MYC* was normalized to *RNaseP*. Representative *MYC* FISH image of KP-4 cells at interphase. Dual color FISH probes to *MYC* (red) and chromosome 8 centromere (green) were used, and nuclei were co-stained with DAPI. Scale bar represents 20 µm. Data from one of two independent experiments are shown. **f**, Mice bearing PSN-1 CDX tumors (n = 6 per group) were orally administered with daraxonrasib at 25 mg kg^−1^ or vehicle control daily. Tumor volumes were assessed through the treatment period (mean ± SEM). Vehicle control and treatment group were compared by two-way repeated-measures ANOVA on the last measurement day of the control group (**, P < 0.01). **g**, PSN-1 tumors relapsed on daraxonrasib at 25 mg kg^−1^ po qd were collected for ddPCR analysis. Relative copy numbers of *KRAS*^*G12R*^, wild-type *KRAS* and *MYC* were normalized to *RPP30* (mean ± SEM; control, n = 4; daraxonrasib, n = 3). Source data

To study the causal role of each candidate resistance mechanism and to evaluate potential combination approaches to mitigate resistance, we adopted two complementary approaches to develop preclinical models: (1) targeted genetic engineering introducing candidate genomic resistance drivers into treatment-naive models, and (2) isolation of resistant clones from initially treatment‑naive tumor models that outgrew under sustained in vivo daraxonrasib (or a relevant RAS(ON) inhibitor) treatment pressure with subsequent molecular characterization and serial passaging under continued RAS(ON) inhibition in vitro and/or in vivo (Fig. 3, Extended Data Figs. 4 and 5, Supplementary Table 5 and Methods). The majority of key acquired resistance mechanisms identified in the EOT ctDNA samples (for example, mutant *KRAS*^*amp*^, RTK alterations and *MYC*^*amp*^) were recapitulated in daraxonrasib-resistant preclinical models derived following prolonged RAS(ON) inhibition.

### RAS pathway hyperactivation drives resistance to daraxonrasib

We then tested the hypothesis that acquired mutant *KRAS*^*amp*^, the most frequently observed genomic alteration in ctDNA, would result in hyperactivation of RAS pathway flux and thereby cause attenuation of daraxonrasib response in tumors.

To model KRAS overexpression resulting from mutant *KRAS*^*amp*^, we engineered the human PDAC cell line HuP-T3 (*KRAS*^*G12R*^) with a doxycycline-inducible *KRAS*^*G12R*^ construct and assessed doxycycline concentration-dependent response to daraxonrasib. We also engineered a murine PDAC organoid-derived cell line model AP009 with different levels of cytomegalovirus promoter-driven *Kras*^*G12D*^ overexpression. We found that increasing levels of *KRAS*^*G12R*^ in HuP-T3 cells or of *Kras*^*G12D*^ in AP009 proportionally modulated pERK levels and attenuated response to daraxonrasib, resulting in decreased antiproliferative potency and depth of response in vitro (Fig. 3a,b and Extended Data Fig. 5a).

We also generated a RAS(ON) multi- and RAS(ON) G12D-selective inhibitor cross-resistant cell line derived from HPAC *KRAS*^*G12D*^ xenograft tumors^26^ (Fig. 3d and Extended Data Fig. 5b). Consistent with the in vivo model, this cell line was cross-resistant to daraxonrasib and zoldonrasib (Fig. 3c and Extended Data Fig. 5c) and exhibited acquired *KRAS*^*G12D*^ amplification resulting in increased KRAS protein levels and proportional modulation of pERK (Fig. 3c), as well as acquired *MAP2K1* and *PIK3CA* mutations. Together, these data link mutant *KRAS* amplification to increased RAS signaling and attenuated daraxonrasib response.

We generated a series of xenograft models of PDAC to interrogate the impact of RAS pathway hyperactivation on daraxonrasib sensitivity in vivo. A serially passaged daraxonrasib-resistant Capan-1 *KRAS*^*G12V*^ homozygous cell line-derived xenograft (CDX) (Fig. 3e,f) revealed elevated HER2 protein expression localized to the cell membrane (Fig. 3g). This observation is consistent with previous studies reporting the upregulation of *ERBB2/HER2* following genetic or pharmacological downregulation of RAS pathway signaling^27^ and with acquired resistance driven by RTK genomic alterations identified in EOT ctDNA of patients 8, 14, 15 and 21.

Recently, we characterized the HPAF-II CDX model that exhibits baseline amplification of *KRAS*^*G12D*^ (copy number of 6)^26^. In this model, RMC-7977, a RAS(ON) multi-selective inhibitor preclinical tool representative of daraxonrasib^28^, treatment drove initial tumor regressions with eventual relapse observed after long-term dosing (Fig. 3h), providing a useful model with baseline *KRAS*^*amp*^ in which to examine combination therapies. In HPAF-II tumors growing out under constant therapeutic pressure with RAS(ON) inhibition (Fig. 3i) we observed reactivation of RAS signaling, as demonstrated by elevated pERK and pMEK levels (Fig. 3j).

We also utilized two relatively insensitive models in which daraxonrasib monotherapy drives an initial response followed by relapse on treatment. The *KRAS*^*G12D*^ KP-4 CDX model of PDAC (Extended Data Fig. 5d) also harbors *MYC* amplification and elevated EGFR and c-MET expression, the latter previously reported to hyperactivate RTK signaling via an HGF/c-MET autocrine loop^29^ (Extended Data Fig. 5e). The *KRAS*^*G12R*^ mutant PSN-1 xenograft model showed evidence for mutant *KRAS*^*amp*^ and *MYC* amplification upon relapse (Extended Data Fig. 5f,g). *MYC* amplification was observed in ctDNA of patient 1, whereas elevated c-MET expression models genomic alterations in patients 9 and 16.

Collectively, this curated series of preclinical models consistently mirrored the findings in the ctDNA of patients with PDAC progressing on daraxonrasib monotherapy. We then used these models to test combination therapeutic strategies aimed at averting or overcoming daraxonrasib resistance.

### Combination approaches to treat monotherapy resistance to daraxonrasib

**Extended Data Fig. 6:**
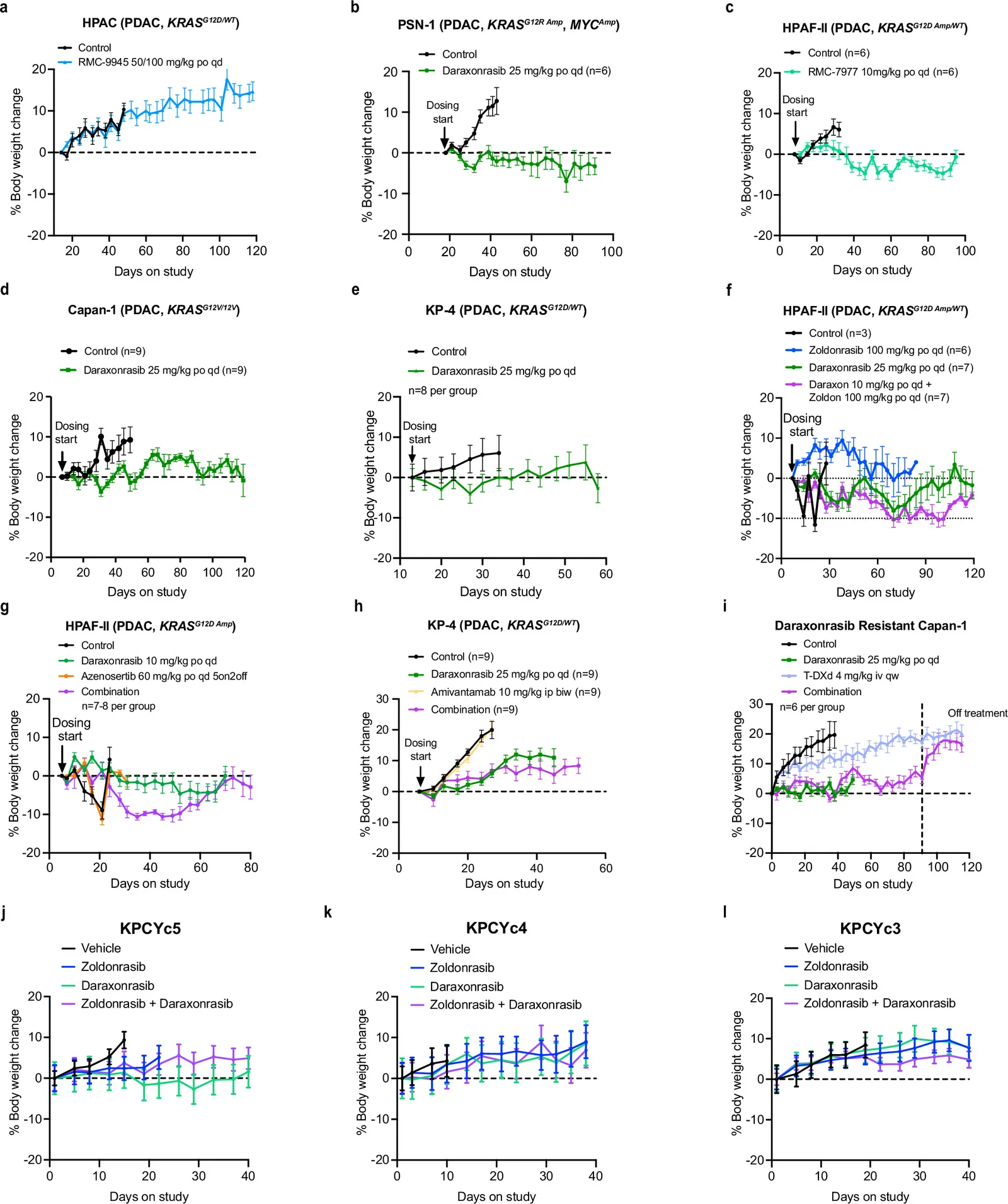
Body Weight characteristics of models and treatments used in the study. **a**, HPAC (n = 6 per group), **b**, PSN-1 (n = 6 per group), **c**, HPAF-II (n = 6 per group), **d**, Capan-1 (n = 9 per group), **e**, KP-4 (n = 8 per group), **f**, HPAF-II (control, n = 3; zoldonrasib, n = 6; daraxonrasib, combination, n = 7 per group), **g**, HPAF-II (control and zenosertib, n = 8 per group; daraxonrasib and combination, n = 7 per group), **h**, KP-4 (n = 9 per group), **i**, Daraxonrasib-resistant Capan-1 (n = 6 per group), **j**, KPCYc5 (n = 9–10 per group), **k**, KPCYc4 (n = 6–7 per group), **l**. KPCYc3 (n = 9–10 per group). Dotted line indicates the initial average body weight of each group. Error bars indicate SEM. Source data

**Extended Data Fig. 7:**
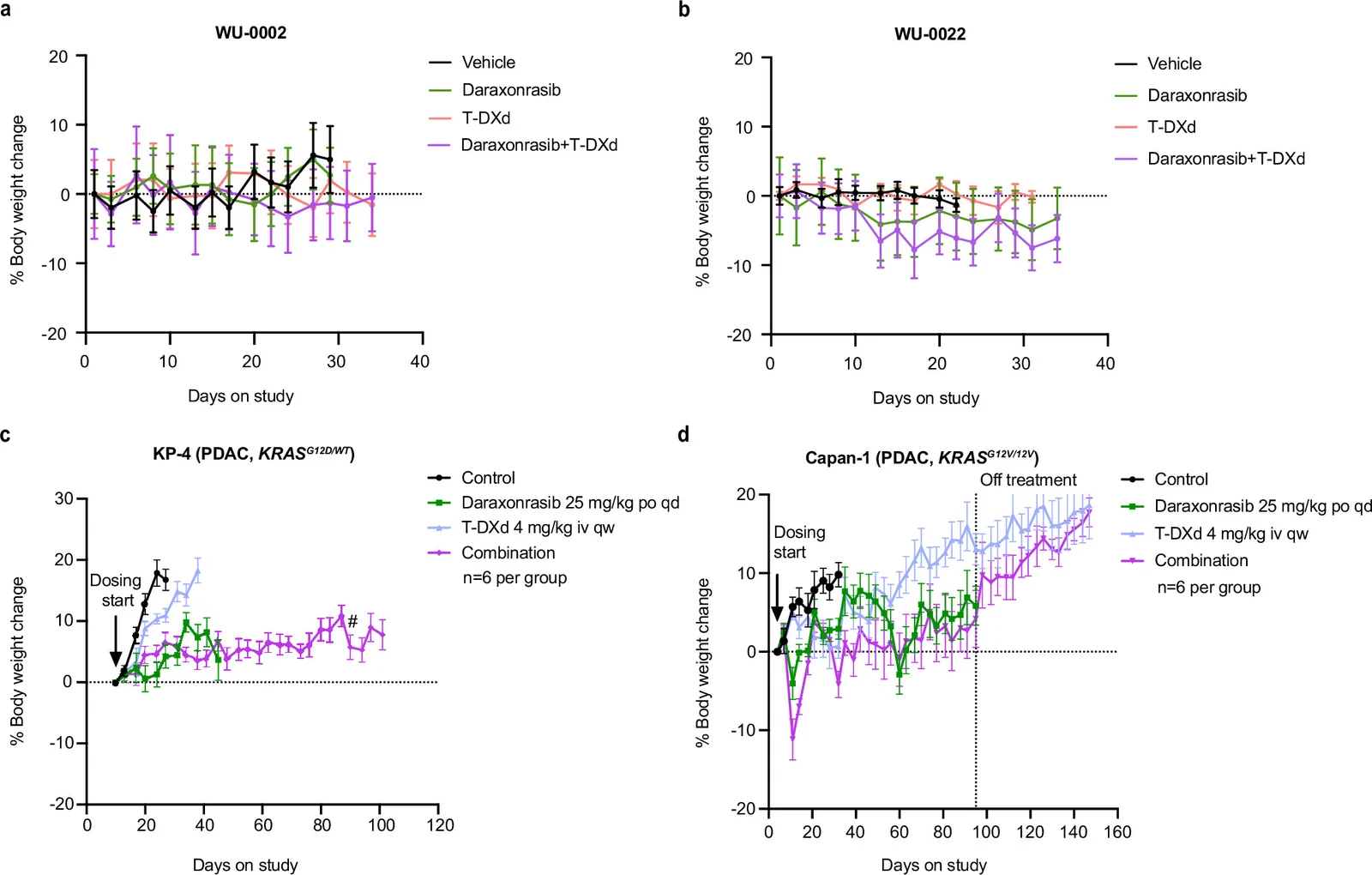
Body Weight characteristics of models and treatments used in the study. **a**, WU-0002 (control and combination, n = 5 per group; daraxonrasib and T-DXd, n = 6 per group), **b**, WU-0022 (control, n = 4; daraxonrasib, n = 5; T-DXd, n = 6; combination, n = 7), **c**, KP-4 (n = 6 per group), **d**, Capan-1 (n = 6 per group). Dotted line indicates the initial average body weight of each group. Error bars indicate SEM. # indicates that one mouse was euthanized. Source data

Where possible, we tested whether combination regimens could be effective in models with acquired resistance to daraxonrasib as well as in treatment-naive models, the latter to evaluate the potential of the upfront combination to delay or prevent said resistance. All treatment regimens included in this study and described below were tolerated in mice based on body weight assessment (Extended Data Figs. 6 and 7).

### Orthogonal combinations with daraxonrasib

**Extended Data Fig. 8:**
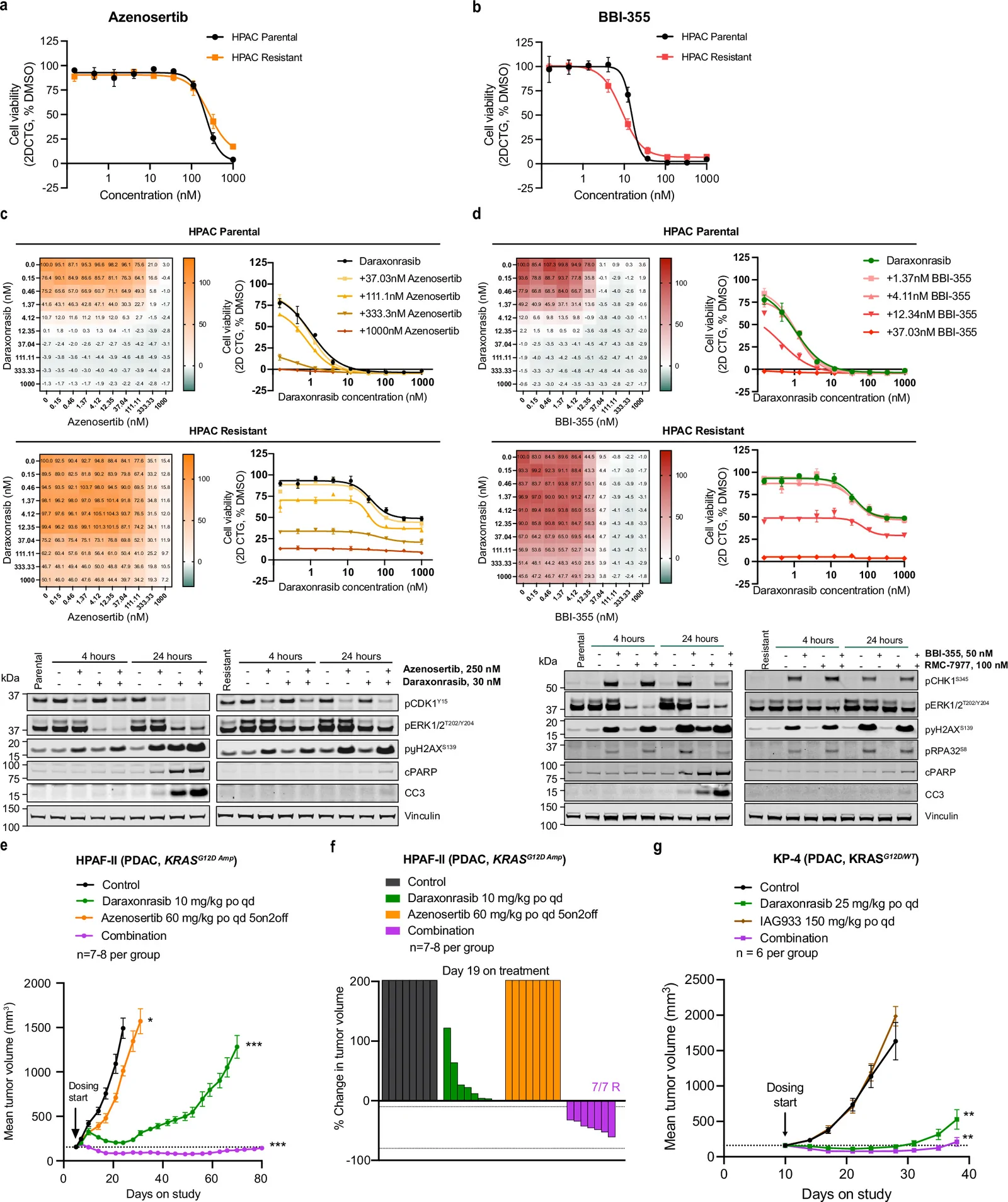
Orthogonal combinations with daraxonrasib. **a, b**, 5-day 2D cellular viability assay was performed using HPAC parental and resistant lines to test the sensitivity to azenosertib, or BBI-355. Dose response curves of cells treated with the indicated concentrations of azenosertib or BBI-355. Data from one of two independent experiments are shown. Technical triplicates were included in each experiment data represent means ± standard deviations. **c** and **d**, upper panels, 5-day 2D cellular viability assay was performed with HPAC parental and resistant cell lines to assess sensitivity to combination treatments of daraxonrasib and azenosertib or BBI-355. Data were presented in a matrix as % of DMSO and as dose response curves. Data from one of two independent experiments are shown. Technical duplicates were included in each experiment and data represent means ± standard deviations Lower panels, Immunoblots of lysates from HPAC parental and resistant cell lines at baseline or treated with DMSO control, RMC-7977 (100 nM) or BBI-355 (50 nM) as single agents or combination for 4 and 24 hours. Primary antibodies to pharmacodynamic (PD; pCHK1^S345^, pERK^T202/Y204^), DNA Damage Response (DDR; pgH2AX^S139^, RPA32^S8^) and apoptosis (cPARP and CC3) markers shown. Vinculin was used as loading control**. e,f**, Mice bearing HPAF-II CDX tumors (control and zenosertib, n = 8 per group; daraxonrasib and combination, n = 7 per group) were orally administered with either vehicle control, daraxonrasib at 10 mg kg^−1^ po qd, azenosertib at 60 mg kg^−1^ po for repeating cycles of 5 days on and 2 days off, or the combination. Tumor volumes were assessed through the treatment period (mean ± SEM). Vehicle control and treatment groups were compared by two-way repeated-measures ANOVA on the last measurement day of the control group (*, P < 0.05; ***, P < 0.001). The dotted line indicates the initial average tumor volume. The waterfall plot shows the percentage change in individual tumor volumes after 19 days of treatment. **g**, Mice bearing KP-4 CDX tumors (n = 6 per group) were orally administered with vehicle control, daraxonrasib at 25 mg kg^−1^ po qd, IAG933 at 150 mg kg^−1^ po qd, or the combination. Tumor volumes were assessed through the treatment period (mean ± SEM). R = regression; CR = complete regression. Vehicle control and treatment groups were compared by two-way repeated-measures ANOVA on the last measurement day of the control group (**, P < 0.01). The dotted line indicates the initial average tumor volume. Source data

We observed in clinical samples that mutant *KRAS*^*amp*^, as a frequent potential resistance driver, was associated with oncogenic LOF *TP53* alterations in pretreatment ctDNA (Fig. 2g). Loss of p53 function and/or KRAS alteration can induce replication stress, creating vulnerability to DNA damage response (DDR) inhibitors^30^. We therefore evaluated daraxonrasib in combination with inhibitors of the ATR–CHK1–WEE1 axis. We evaluated BBI-355 (a CHK1 inhibitor)^31^ and azenosertib (a WEE1 inhibitor)^32^ in the parental and daraxonrasib-resistant *KRAS*^*G12D*^; *TP53*^*WT*^ HPAC cell line models. Both models were sensitive to single-agent DDR inhibition and combination treatment (Extended Data Fig. 8a–d). Notably, in the resistant cell line with mutant *KRAS*^*amp*^ antiproliferative effects were driven primarily by DDR inhibition, indicating increased DDR dependence (Extended Data Fig. 8c,d). Daraxonrasib reduced pERK and pCDK1 in both parental and resistant lines, albeit with more rapid restoration of pERK in the resistant model suggesting RAS hyperactivation driven by *KRAS*^*G12D*^ amplification. Combination treatment increased DDR pathway activation marker γH2AX and induced apoptosis (cleaved caspase-3, PARP) in parental cells, with only modest effects in resistant cells (Extended Data Fig. 8c,d). We then assessed the combination of daraxonrasib and the WEE1 inhibitor azenosertib in vivo in the parental *KRAS*^*G12D*^ amplified; *TP53*^*R273H*^ HPAF-II CDX PDAC tumor model. Azenosertib showed minimal single-agent activity, while daraxonrasib induced transient tumor stasis followed by regrowth; the combination achieved deeper and more durable regressions, though not exceeding optimal-dose daraxonrasib monotherapy (Extended Data Fig. 8e,f). Together, these data support DDR machinery as a therapeutic vulnerability in RAS mutant PDAC.

We next explored strategies targeting *MYC*^*amp*^-mediated resistance, observed in patient 1 and previously in the KPC genetically engineered mouse model (GEMM)^33^. Although prevalent in GEMMs, *MYC*^*amp*^ is less frequent in KRAS G12C(OFF) inhibitor-treated PDAC and in this cohort^19^. We previously discovered that the combination of TEA domain transcription factor and RAS(ON) multi-selective inhibition was effective in the context of *MYC*^*amp*^ in vitro^33^; we now evaluated the combination of daraxonrasib with the pan-TEA domain transcription factor inhibitor IAG933 in *MYC*^*amp*^ KP-4 CDX tumors (along with additional candidate resistance drivers). IAG933 as a single agent was inactive, while daraxonrasib induced transient response (Extended Data Fig. 8g). The combination achieved deeper responses but did not prevent on-treatment relapse, suggesting the combinatorial potential of this approach may be limited (at least in this model).

### Engaging RTKs in combination with RAS(ON) multi-selective inhibition

**Fig. 4:**
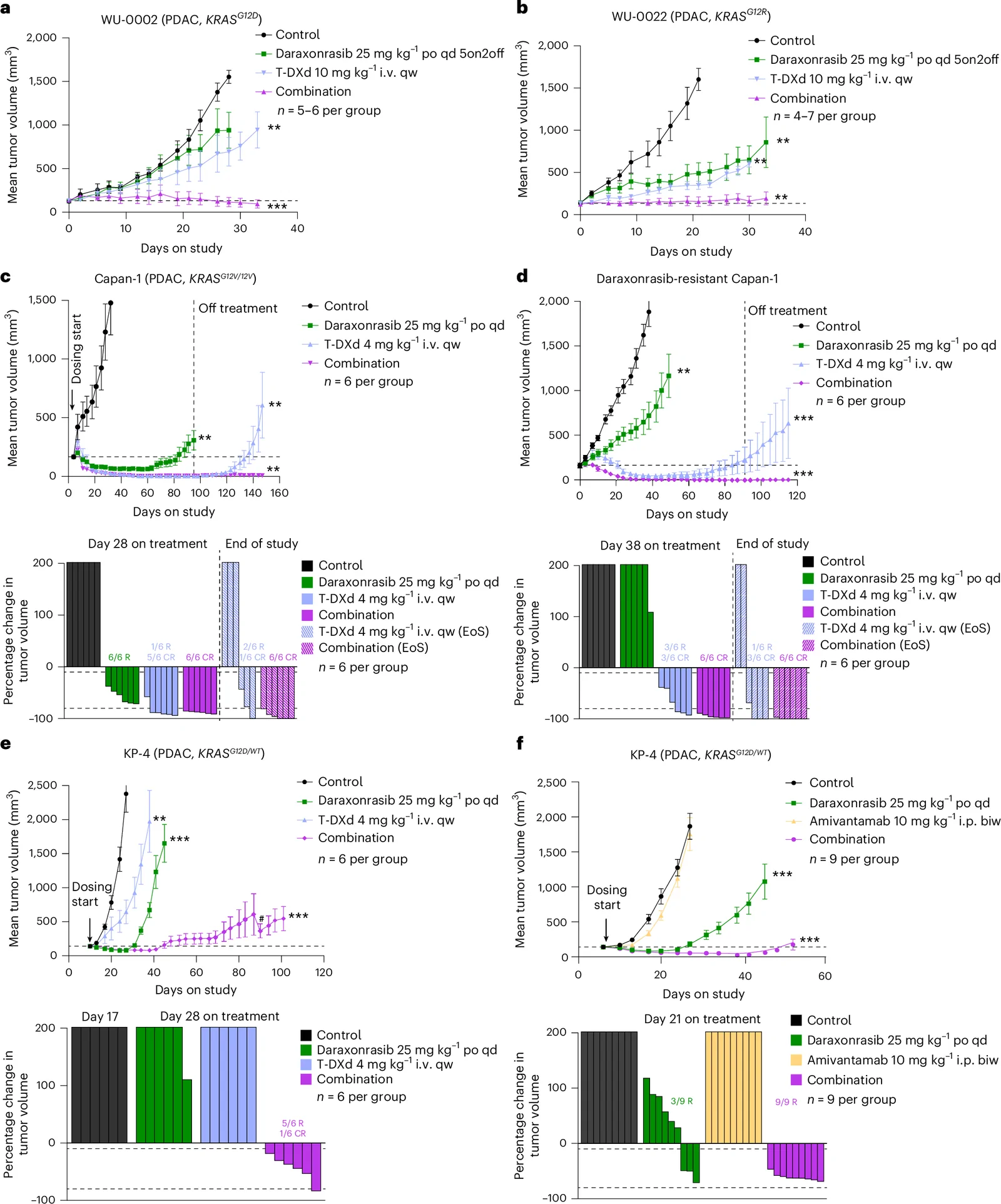
Combinations of daraxonrasib and RTK targeting agents overcome tumor growth in models with RTK activation. **a**,**b**, Mice bearing WU-0002 (**a**) or WU-0022 patient-derived (**b**) xenograft (PDX) tumors were treated with vehicle, daraxonrasib at 25 mg kg^−1^ po for repeating cycles of 5 days on and 2 days off, T-DXd intravenously (i.v.) at 10 mg kg^−1^ per week, or the combination. Group sizes were: WU-0002, *n* = 5 for vehicle and combination and *n* = 6 for daraxonrasib and T-DXd; WU-0022, *n* = 4 for vehicle, *n* = 5 for daraxonrasib, *n* = 6 for T-DXd and *n* = 7 for combination. Tumor volumes were assessed through the treatment period (mean ± s.e.m.). Vehicle control and treatment groups were compared by two-way repeated-measures ANOVA on the last measurement day of the control group (**P < 0.01, ***P < 0.001). **c**,**d**, Mice bearing parental (**c**) or daraxonrasib-resistant Capan-1 (**d**) CDX tumors (*n* = 6 per group) were treated with vehicle, daraxonrasib (25 mg kg^−1^, po qd), T-DXd (4 mg kg^−1^, i.v., weekly) or the combination. Waterfall plots show the percentage change in individual tumor volumes after the indicated treatment duration. **e**, Mice bearing KP-4 CDX tumors (*n* = 6 per group) were treated with vehicle control, daraxonrasib at 25 mg kg^−1^ po qd, T-DXd i.v. at 4 mg kg^−1^ per week, or the combination. # indicates that one mouse was euthanized. **f**, Mice bearing KP-4 CDX tumors (*n* = 9 per group) were treated with vehicle, daraxonrasib (25 mg kg^−1^ po qd), amivantamab (10 mg kg^−1^, intraperitoneally (i.p.) twice weekly) or the combination. Mean tumor volumes and individual percentage changes across the course of treatment are shown. For all studies, tumor volumes are shown as mean ± s.e.m.; vehicle and treatment groups were compared by two-way repeated-measures ANOVA on the final control-group measurement day (**P < 0.01, ***P < 0.001). biw, twice weekly; R, regression; qw, once weekly. Source data

**Extended Data Fig. 9:**
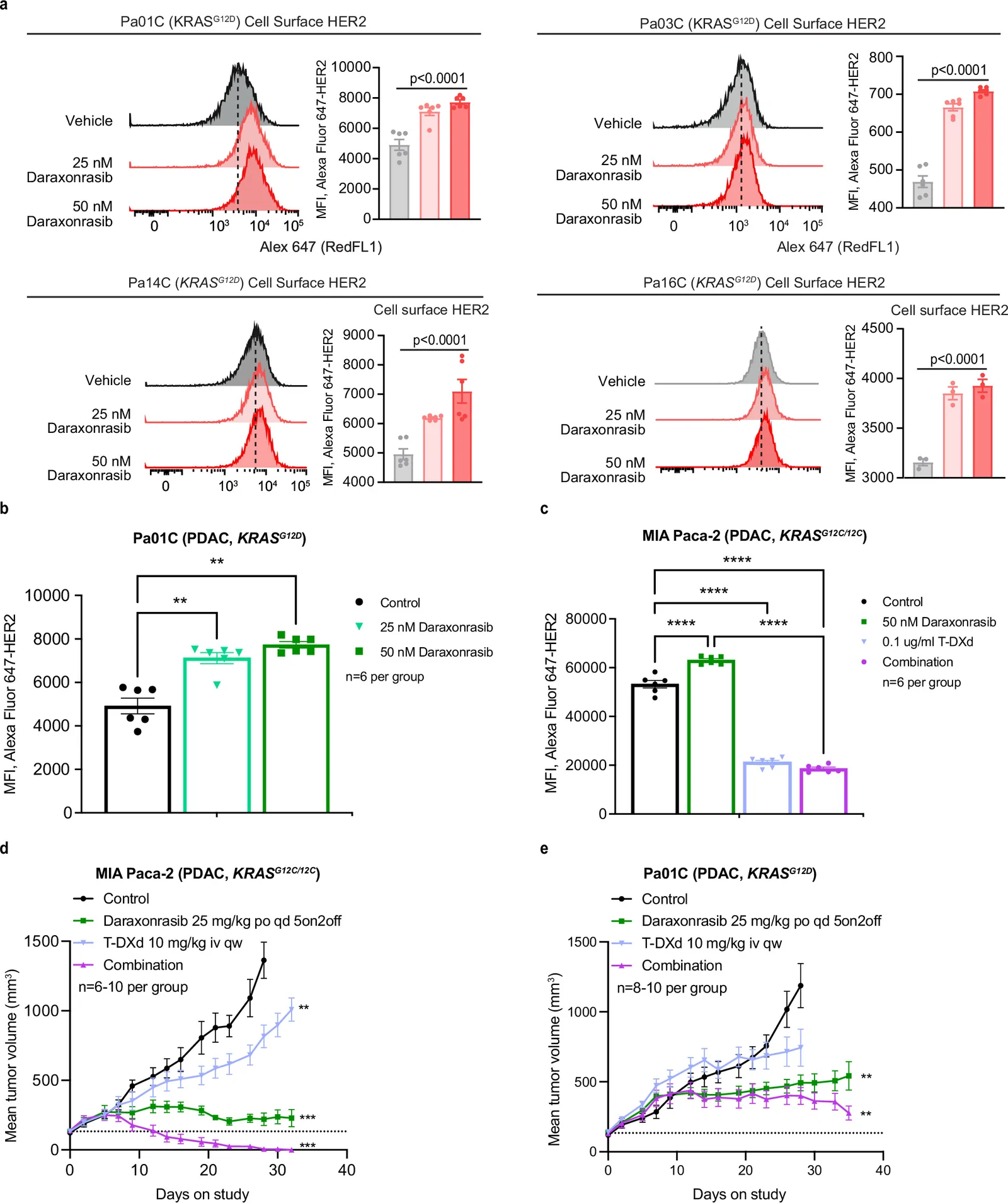
Expression of HER2 on cell surface of preclinical models and combination benefit mediated by T-DXd and daraxonrasib. **a, b**, and **c**, HER2 surface levels in indicated cells were assessed after treatment with daraxonrasib (0.05 μM), T-DXd (0.1 μg/mL), or combination. Daraxonrasib treatment increased surface HER2 expression, while subsequent treatment with T-DXd (DS-8201a) led to efficient HER2 internalization. Notably, MIA PaCa-2 cells exhibit higher basal HER2 expression compared to other PDAC lines. All experiments were performed independently twice, each in triplicate. The treatment groups were compared with vehicle control using one-way ANOVA followed by Dunnett multiple comparison test (**, P < 0.01; ****, P < 0.0001). **d,e**, Tumor growth kinetics of the indicated PDAC models implanted subcutaneously into NOD-SCIDγ mice. Treatments were initiated when tumors reached 100–150 mm^3^. Mice bearing MIA PaCa-2 tumors (control, n = 9; daraxonrasib, n = 6; T-DXd, n = 7; combination, n = 10) or Pa01C tumors (control, daraxonrasib, and combination, n = 8 per group; T-DXd, n = 10) were treated as indicated. Daraxonrasib was administered po at 25 mg kg^−1^ for 5 days per week (5 days on, 2 days off), and T-DXd was given via tail vein injection at 10 mg kg^−1^ per week. Mean tumor volumes of each group were plotted across the course of treatment (mean ± SEM). Vehicle control and treatment groups were compared by two-way repeated-measures ANOVA on the last measurement day of the control group (**, P < 0.01; ***, P < 0.001). Source data

Based on the prevalence of RTK alterations in our ctDNA analyses and daraxonrasib‑induced RTK upregulation in the Capan‑1 model, we hypothesized that combining RAS(ON) multi-selective inhibition with therapies that leverage RTKs as targets could mitigate resistance and restore tumor sensitivity. Consistent with previous studies with RAS pathway inhibitors^27^, we observed that daraxonrasib treatment resulted in a concentration-dependent increase in surface HER2 expression, though the magnitude of induction varied between cell lines (Extended Data Fig. 9a,b)^34^. We hypothesized that the collateral vulnerability of HER2 upregulation at the tumor cell membrane would drive internalization of the antibody–drug conjugate (ADC) trastuzumab deruxtecan (T-DXd)-modified receptor and delivery of a cytotoxic payload, which would synergize with daraxonrasib-mediated RAS pathway inhibition. In the MIA Paca-2 cell line^27^, daraxonrasib further enhanced surface HER2 levels, and subsequent treatment with T-DXd led to rapid and efficient HER2 internalization, suggesting functional target engagement (Extended Data Fig. 9c). We then assessed the in vivo antitumor activity of daraxonrasib in combination with T-DXd in four xenograft models of PDAC harboring distinct *KRAS* mutations: MIA Paca-2 (*KRAS*^*G12C*^), WU-0022 (*KRAS*^*G12R*^), WU-0002 (*KRAS*^*G12D*^) and Pa01C (*KRAS*^*G12D*^)^27^. In the first three models, the treatment resulted in combinatorial activity and sustained tumor regressions (Fig. 4a,b and Extended Data Fig. 9d). We did not observe a meaningful combinatorial effect in the Pa01C model (Extended Data Fig. 9e), which shows very low initial levels of surface HER2 with minimal enhancement following daraxonrasib treatment, and no response to T-DXd monotherapy, indicating that the combination may require a threshold level of HER2 expression, robust ADC internalization, and/or baseline sensitivity to topoisomerase inhibition (Extended Data Fig. 9b). We further evaluated this combination in the parental and daraxonrasib-resistant Capan-1 CDX model pair described above, simulating upfront and sequential combination treatment paradigms, respectively. In both models, the combination led to dramatic complete and durable tumor regressions in Capan-1 parental tumors, which were sustained even after treatment cessation (Fig. 4c,d). Consistent with these findings, the combinatorial activity of daraxonrasib and T-DXd was also observed in the high-bar KP-4 CDX model, where the combination achieved more durable tumor responses (Fig. 4e).

Given the detection of c-MET alterations in the ctDNA of some patients who progressed on daraxonrasib monotherapy and the reciprocal relationship of c-MET and EGFR in the context of KRAS G12C(OFF) inhibitor resistance in PDAC^35^, we hypothesized that the combined inhibition of c-MET and EGFR may effectively block acquired RTK-mediated daraxonrasib resistance. We therefore modeled the combination of daraxonrasib and amivantamab, a bispecific inhibitory antibody against EGFR and MET^36^, in the KP-4 CDX model which expresses high levels of c-MET^33^. Daraxonrasib monotherapy drove initial tumor regressions with tumor outgrowth on treatment observed after 14 days, while the combination of daraxonrasib and amivantamab resulted in a significant improvement in both the depth and duration of tumor responses as compared to either agent alone (Fig. 4f). In sum, these results support the combination of RTK targeting therapeutic modalities such as ADCs and bispecific agents with daraxonrasib to overcome resistance driven by RTK overexpression and activation.

### Doubling down on RAS pathway signaling by combining RAS(ON) multi- and mutant-selective inhibitors

**Fig. 5:**
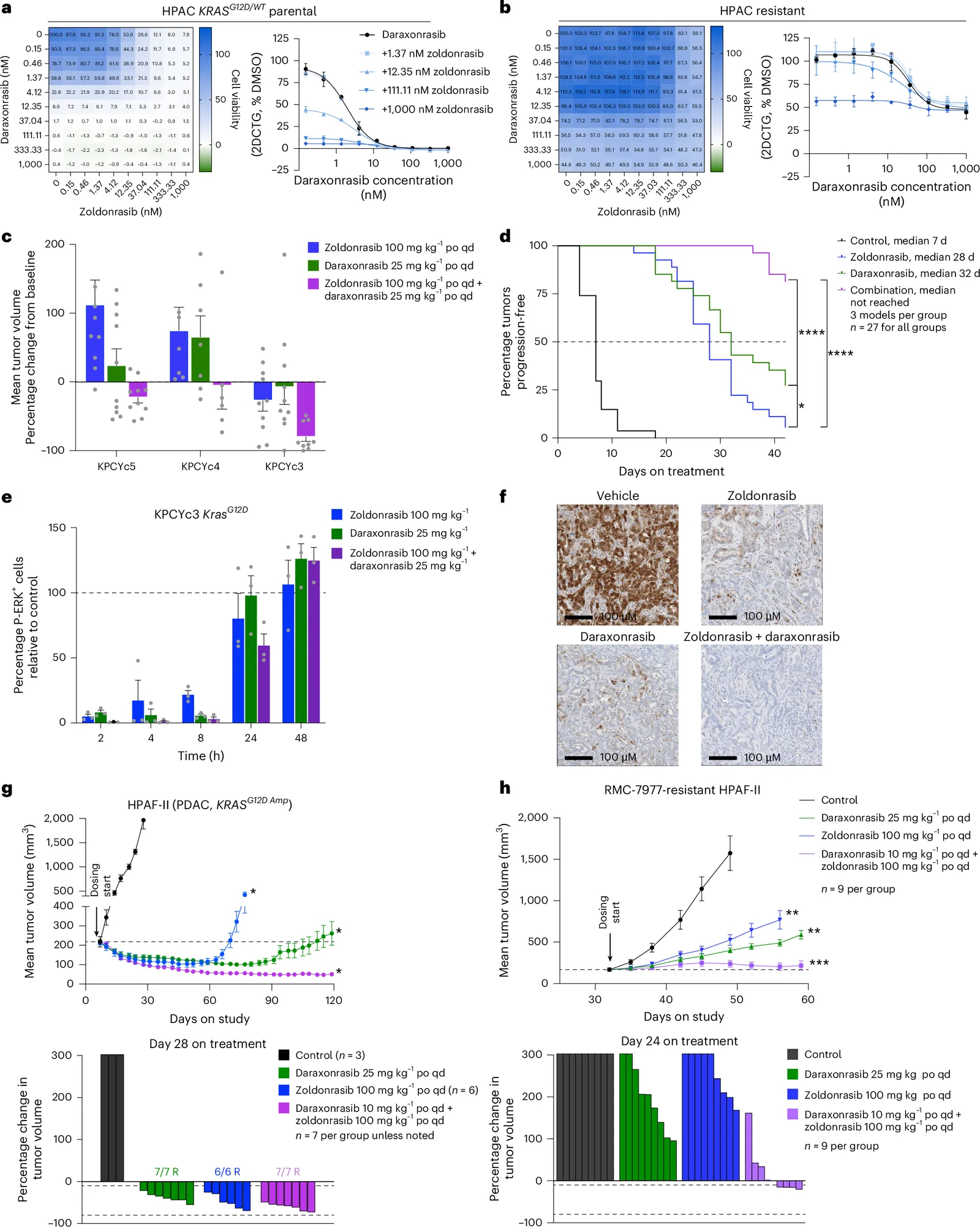
Doubling down on RAS pathway signaling by combining RAS(ON) multi- and mutant-selective inhibitors. **a**,**b**, Five-day 2D viability assays were performed in parental (**a**) and resistant (**b**) HPAC cell lines treated with daraxonrasib, zoldonrasib or the combination. Data are shown as heatmaps (% of DMSO control) and dose–response curves. One of two independent experiments is shown; technical duplicates were included, and data represent mean ± s.d. **c**, Mice bearing KPC/Y GEMM-derived tumors (KPCYc3, *n* = 10 per group except combination *n* = 9; KPCYc4, *n* = 7 per group except combination *n* = 6; KPCYc5, *n* = 10 per group) were treated with vehicle, daraxonrasib (25 mg kg^−1^ po qd), zoldonrasib (100 mg kg^−1^ po qd) or the combination. Mean tumor volume change from baseline on day 21 is shown (mean ± s.e.m.). **d**, Combined Kaplan–Meier curves from experiments in **c** representing PFS, with progression defined as tumor doubling from baseline on treatment (*n* = 27 mice per treatment group). log-rank test *P = 0.0467, ****P < 0.0001. **e**,**f**, Pharmacodynamic effects were assessed in KPCYc3 tumors following a single dose of daraxonrasib, zoldonrasib or the combination. Mean pERK positivity in pan cytokeratin (panCK)-positive tumor cells was quantified by IHC relative to vehicle (mean ± s.e.m., *n* = 3 mice per group per time point; **e**) and representative pERK IHC images (**f**) at 8 h post-dose are shown. **g**, HPAF-II tumor-bearing mice were treated with vehicle (*n* = 3), daraxonrasib (25 mg kg^−1^; *n* = 7), zoldonrasib (100 mg kg^−1^; *n* = 6) or daraxonrasib (10 mg kg^−1^) plus zoldonrasib (100 mg kg^−1^; *n* = 7). Tumor growth and individual tumor responses after 28 days are shown. **h**, Mice bearing resistant HPAF-II tumors (*n* = 9 per group) received the same treatments. Tumor growth and individual responses after 24 days are shown. For **g** and **h**, data represent mean ± s.e.m.; groups were compared by two-way repeated-measures ANOVA on the final vehicle-group measurement day (*P < 0.05, **P < 0.01, ***P < 0.001). Source data

**Extended Data Fig. 10:**
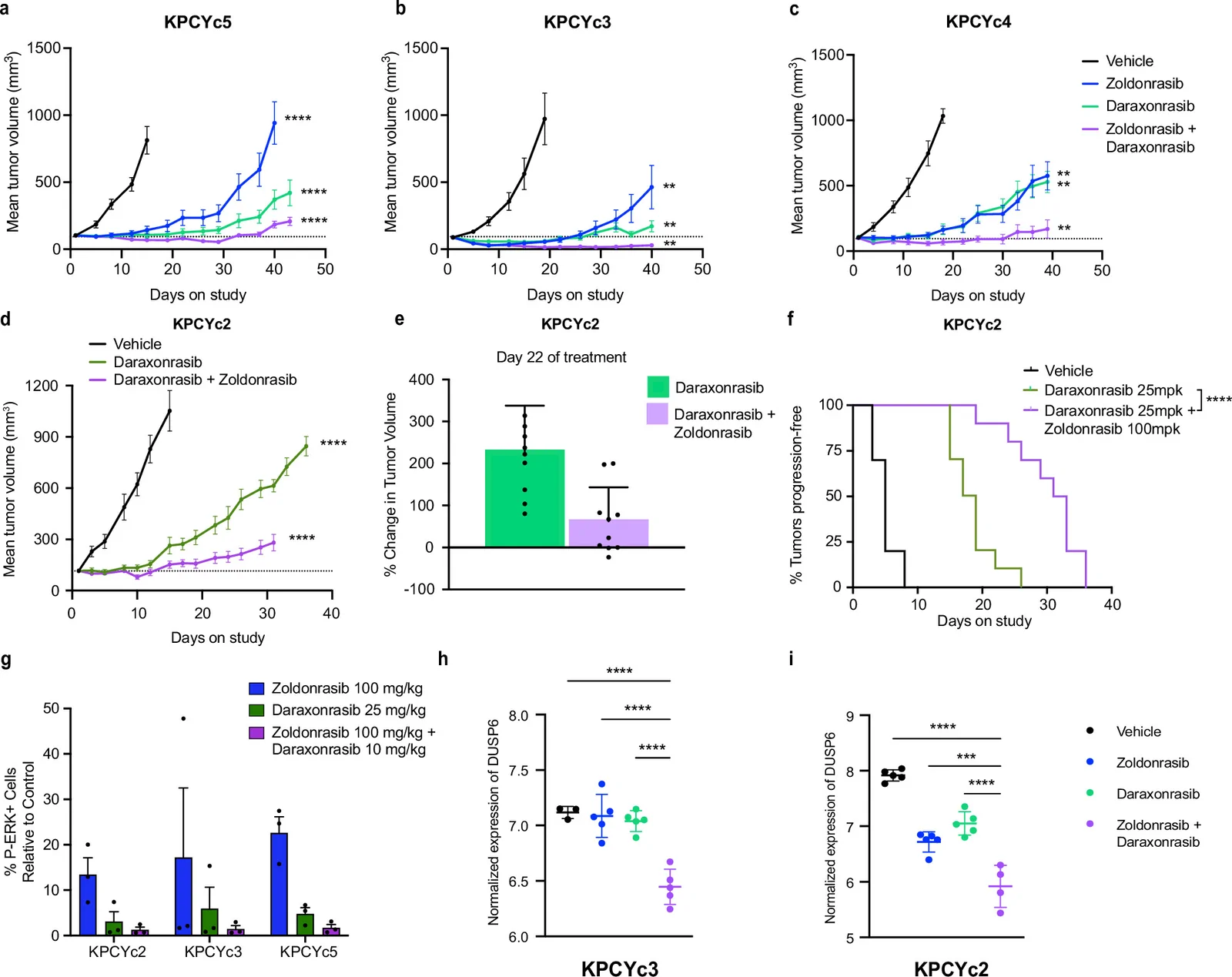
Doubling down on RAS pathway by combining zoldonrasib and daraxonrasib in immunocompetent KPCY models. **a-c**, Mice bearing KPC/Y GEMM-derived tumors were treated with vehicle control, daraxonrasib at 25 mg kg^−1^ po qd, zoldonrasib at 100 mg kg^−1^ po qd, the combination of both. Tumor volumes were assessed through the treatment period (mean ± SEM). Vehicle control and treatment groups were compared by two-way repeated-measures ANOVA on the last measurement day of the control group. **a**, KPCYc5, n = 10 mice per treatment group. **** p < 0.0001. **b**, KPCYc3 n = 10 mice per group except daraxonrasib + zoldonrasib combination group n = 9 mice. ** p = 0.0037 for zoldonrasib group compared to vehicle group, ** p = 0.0033 for daraxonrasib group compared to vehicle group, ** p = 0.0039 for combination group compared to vehicle group. **c**, KPCYc4 n = 7 mice per treatment group. ** p = 0.0028 for zoldonrasib group compared to vehicle group, ** p = 0.0029 for daraxonrasib group compared to vehicle group, ** p = 0.0021 for combination group compared to vehicle group. **d-f**, Mice bearing KPC/Y GEMM-derived KPCYc2 tumors were treated with vehicle control, daraxonrasib at 25 mg kg^−1^ po qd, or the combination of daraxonrasib at 25 mg kg^−1^ and zoldonrasib at 100 mg kg^−1^ po qd (n = 10 mice per treatment group). **d**, Tumor volumes were assessed through the treatment period (mean ± SEM). Vehicle control and treatment groups were compared by two-way repeated-measures ANOVA on the last measurement day of the control group (**** p < 0.0001). **e**, Data represent mean tumor volume change from baseline on day 21 of treatment (mean ± SEM). **f**, Kaplan-Meier curves represent progression-free survival, with progression defined as tumor doubling from baseline. Log-rank test **** p < 0.0001. **g**, Mean percentage (%) of pERK positivity as measured by IHC in pan Cytokeratin positive tumor cells relative to vehicle control group, following treatment of mice bearing KPC/Y GEMM-derived tumors (KPCYc2, KPCYc3, and KPCYc5) with a single oral dose of zoldonrasib at 100 mg kg^−1^, daraxonrasib at 25 mg kg^−1^, or the combination of daraxonrasib at 10 mg kg^−1^ and zoldonrasib at 100 mg kg^−1^. pERK % in the vehicle control group is set to 100%. Data represent mean ± SEM (n = 3 mice per group for each model). **h, i**, *Dusp6* expression assessed by bulk RNAseq analysis of KPC/Y GEMM-derived tumors treated for 8 days with vehicle control, daraxonrasib at 25 mg kg^−1^ po qd, zoldonrasib at 100 mg kg^−1^ po qd, or the combination of daraxonrasib at 10 mg kg^−1^ and zoldonrasib at 100 mg kg^−1^, and collected 24 h after last dose. Treatment groups were compared by one-way Anova. **h**, KPCYc3, n = 5 mice per treatment group except vehicle n = 3 mice. **** p < 0.0001. **i**, KPCYc2, n = 5 mice per treatment group except zoldonrasib + daraxonrasib combination n = 4 mice. *** p = 0.0003 for zoldonrasib group compared to combination group, **** p < 0.0001 for vehicle and daraxonrasib groups compared to combination group. Source data

Next, we hypothesized that daraxonrasib-resistant models with dependency on elevated RAS signaling could benefit from a combination of multi- and mutant-selective RAS(ON) inhibitors, maximizing direct inhibition of RAS signaling through both mutant and WT RAS, analogous to our work demonstrating this combination concept in KRAS G12C‑driven lung cancer models^37^. In the HPAC parental cell line model, the combination resulted in a strong antiproliferative effect and cell killing in vitro^26^ (Fig. 5a). In the daraxonrasib (and zoldonrasib)-resistant HPAC line, we observed a combination benefit that was evident but somewhat reduced as compared to that in the parental line, consistent with increased oncogenic flux through the RAS pathway in this context (Fig. 5b). Therefore, we hypothesized that the upfront combination therapy of a RAS(ON) inhibitor doublet would be more effective in forestalling the development of RAS-driven resistance rather than sequential treatment in the context of established monotherapy resistance. We tested the upfront combination therapy in a series of treatment-naive *Kras*^*G12D*^ driven KPC/Y GEMM-derived models in vivo^38^. The RAS(ON) inhibitor doublet had a profound combination effect on antitumor activity, resulting in greater depth of response and significant extension of PFS (as measured by time to tumor doubling from baseline) compared to each monotherapy, with the upfront combination durably preventing the emergence of resistance (Fig. 5c,d and Extended Data Fig. 10a–f). In each of these models, single-agent daraxonrasib and zoldonrasib substantially suppressed RAS pathway signaling as indicated by decreased pERK signal compared with vehicle controls and the RAS(ON) inhibitor doublet induced deeper and more durable reduction of pERK at 4 h and *Dusp6* expression at 24 h post-dose compared to single agents (Fig. 5e,f and Extended Data Fig. 10g–i).

Finally, we examined the zoldonrasib and daraxonrasib combination in the paired parental RAS(ON) multi-selective inhibitor naive and treatment-resistant *KRAS*^*G12D*^ HPAF-II CDX models. In the parental model, zoldonrasib or daraxonrasib as single agents drove initial tumor regressions following relapse and outgrowth on treatment, while the RAS(ON) inhibitor doublet drove deep and durable tumor regressions (Fig. 5g) and averted resistance development, consistent with the results in the KPC/Y models above. In the corresponding resistant model, the RAS(ON) inhibitor doublet showed combinatorial antitumor activity that was significantly improved as compared to either monotherapy activity (Fig. 5h). Taken together, these results imply that a RAS(ON) inhibitor doublet can be deployed either to forestall or to overcome RAS hyperactivation-driven resistance to daraxonrasib monotherapy.

## Discussion

The development of direct RAS inhibitors, beginning with KRAS G12C(OFF) targeting compounds and now continued by the advent of RAS(ON) inhibitors beginning with daraxonrasib, has transformed the treatment of RAS-driven cancers^18,39^. The encouraging monotherapy activity for daraxonrasib in patients with PDAC following standard-of-care chemotherapy^12^ was recently confirmed by results from the RASolute 302 trial^13^. Timely interrogation of monotherapy resistance mechanisms informs rational combination strategies co-targeting key drivers and/or taking advantage of treatment-induced vulnerabilities^18,40^.

Acquired genomic alterations detected in this study converged on RAS pathway hyperactivation, indicating increased oncogenic flux as a dominant escape mechanism. Of note, resistance mechanisms appeared more frequently at higher daraxonrasib doses, suggesting a dose-dependent bottleneck in RAS-driven tumors^41^. Acquired secondary *KRAS* mutations were notably absent in this PDAC resistance study, albeit a parallel study by Sang et al. in non-PDAC tumors found secondary mutations in 2 of 40 patients^14^, distinct from secondary *KRAS* mutations that abrogate the binding of KRAS G12C(OFF) inhibitors^18^; however, acquired resistance to KRAS G12C(OFF) inhibitors in PDAC altogether shows fewer secondary *KRAS* mutations compared with those in NSCLC and CRC^19,39^. Notably, no compensatory oncogenic alterations in *NRAS* or *HRAS* following daraxonrasib treatment were identified, consistent with the broad and concomitant inhibition of active state mutant and WT canonical RAS proteins by daraxonrasib^28^. These observations support a resistance pattern dominated by pathway reactivation rather than on-target *KRAS* second-site mutations.

We demonstrate combinatorial antitumor activity for RTK-targeted biologics combined with daraxonrasib in PDAC models. In the case of T-DXd, RAS inhibition-driven HER2 upregulation may enhance delivery of a cytotoxic payload^19,20^. We also demonstrated that the RAS(ON) inhibitor doublet of daraxonrasib and zoldonrasib (under clinical evaluation; NCT06040541) was well tolerated and drove combinatorial pathway suppression sufficient to prevent resistance in a series of naive PDAC models and to overcome acquired RAS(ON) inhibitor monotherapy resistance. These results support clinical evaluation of this RAS(ON) inhibitor doublet in KRAS G12D-mutant PDAC. Collectively, these data nominate two clinically actionable strategies in PDAC: (1) combinations with RTK-directed biologics (for example, T-DXd and amivantamab) and (2) intensified direct RAS blockade with a RAS(ON) inhibitor doublet (daraxonrasib + zoldonrasib). Additionally, orthogonal combinations of daraxonrasib and DDR inhibitors in mutant *KRAS*^*amp*^ models enhanced antitumor activity albeit the feasibility of such targeted combination approaches will rely on the development of agents targeting the DDR axis with favorable safety profiles. In tumors with *TP53* inactivation, it is possible that chemotherapy-induced DNA damage could mimic replicative stress and functionally overlap with DDR-targeting approaches; combinations with chemotherapy are an active area of preclinical and clinical investigation (see RASolute 303 trial in the frontline setting; NCT07491445).

While this study presents the first comprehensive evaluation of clinical and preclinical genomic mechanisms of acquired resistance to monotherapy daraxonrasib in PDAC, we only describe genomic features detectable by paired ctDNA analyses in patients with sufficient tumor shedding into circulation. The identification of candidate genomic resistance drivers in ~60% of patients examined indicates that ~40% of PDAC tumors that relapse on daraxonrasib treatment potentially acquire resistance via nongenomic means. Moreover, multiple preclinical studies have demonstrated that genetic and nongenetic mechanisms can coevolve within the same tumors^19,40^. Additionally, the detection of alterations in 800 genes in the DNA sequencing panel selected for this study cannot rule out other resistance mutations, including in *PPIA*, the gene encoding cyclophilin A, required for RAS(ON) inhibitor activity^42^. Future studies with larger cohorts and paired tumor biopsies will be needed to validate these findings and define genomic and nongenomic resistance mechanisms. Furthermore, a rigorous evaluation of whether the genomic resistance mechanisms reported in this study are acquired de novo (or pre-existing at baseline) will require data from a much larger patient cohort such as that from RASolute 302 mentioned herein^13^.

In closing, we report the first characterization of treatment-emergent genomic alterations following daraxonrasib response and relapse in patients with PDAC, demonstrating that acquired resistance mechanisms converge on RAS pathway signaling hyperactivation and recapitulate patterns of acquired resistance for inhibitors of BRAF, MEK and RTKs^18^. These results reinforce that RAS/MAPK pathway-dependent cancers are oncogene-addicted and that the evolution of therapeutic resistance often selects for reactivation of driver oncogene signaling. Our insights guided the evaluation of rational combinations to avert and/or overcome daraxonrasib resistance in a series of preclinical models laying a translational foundation for the evaluation of promising combinations in PDAC aimed at increased and durable therapeutic benefit following on recent encouraging monotherapy results in the randomized phase 3 clinical trial RASolute 302 (ref. ^13^).

## Methods

### Ethics statement

The trial was conducted in accordance with the Good Clinical Practice guidelines of the International Council for Harmonization and the principles of the Declaration of Helsinki. The protocol and amendments were approved by the institutional review board at each participating site and regulatory authorities of participating countries. The institutional review boards that approved this study were Advarra, Dana-Farber Cancer Institute, WCG, Salus, John Hopkins Medicine, Memorial Sloan Kettering, Mary Crowley Medical Research Center, UCLA Office of Human Research Protection Program, NYU Grossman School of Medicine and Columbia University Irving Medical Center.

All patients provided written informed consent. The trial was designed by Revolution Medicines (study sponsor). The data were collected by investigators and analyzed by statisticians employed by Revolution Medicines.

All animal studies were conducted in accordance with protocols approved by the Institutional Animal Care and Use Committee at each study site and were performed in compliance with all applicable institutional and national guidelines for the care and use of laboratory animals.

### Statistics and reproducibility

The clinical study NCT05379985 was conducted as a single-arm phase 1/2 trial; therefore, participants were not randomized. Investigators were not blinded to allocation during experiments and outcome assessment unless otherwise indicated.

For preclinical studies, power analyses were used to drive sample size determination as appropriate. Randomization was used in preclinical in vivo studies. Sample sizes for preclinical experiments were determined based on study objectives, previous experience with similar models and practical considerations and were not predetermined using formal statistical methods. Unless otherwise specified, no data were excluded from the analyses.

### Circulating tumor DNA assessment in patients with acquired resistance

Patients with RAS mutant metastatic PDAC treated at 160 mg to 300 mg daraxonrasib as second-line or later therapy and disease progression were assessed for acquired resistance. Acquired resistance was defined as patients with a PFS of more than 3 months (including SD or PR or CR) and subsequent progression, in line with previous criteria applied for patients with acquired resistance^19^. Paired pretreatment and EOT at time of progression ctDNA assessment using the Guardant Health Infinity next-generation sequencing panel with 800+ genes was performed in 44 patients meeting these criteria. Tissue sequencing was also performed in one patient with a paired pretreatment and EOT tissue biopsy.

An acquired alteration in EOT ctDNA was defined as a known or likely oncogenic mutation annotated by OncoKB and not detected in pretreatment ctDNA. To account for false-negative acquired variants due to low shedding in pretreatment ctDNA, pretreatment samples were required to have at least one somatic variant detected to be considered for paired analysis. Acquired oncogenic variants were examined in RAS, MAPK, RTK, PI3K/AKT and MYC pathways based on definitions by Vega et al.^43^. CN alterations were defined as any CN <2 copies for gene deletions and >2 copies for gene amplifications; patients without reported CN alterations in a gene thus had two copies (normal diploid state) detected in total cell-free DNA. Supplementary Table 4 lists all known or likely oncogenic alterations detected in pretreatment ctDNA.

### Animal studies using xenograft and syngeneic tumor models

Studies were conducted at Revolution Medicines, Washington University School of Medicine and at contract research organizations, including Pharmaron and WuXi AppTec. No statistical methods were used to predetermine sample sizes but our sample sizes are similar to those reported in previous publications^33^. All CDX/PDX mouse studies and procedures related to animal handling, care, and treatment complied with all applicable regulations and guidelines of the Institutional Animal Care and Use Committee at each facility with approvals. The maximum tumor burden permitted was 2,000–2,300 mm^3^. Tumor growth was monitored regularly, and animals were killed according to the study protocol. In a small number of cases, due to rapid tumor growth between scheduled measurements, tumor volumes somewhat exceeded 2,000–2,300 mm^3^ just before killing.

For studies with PSN-1 and Capan-1 CDX tumors, female BALB/c nude mice 6–8 weeks old were used. The animals were purchased from ZheJiang Vital River Laboratory Animal Technology Co. and Shanghai Sino-British SIPPR/BK Laboratory Animal Co. For studies with HPAC CDX tumors, female athymic nude mice aged 6 weeks were used. For study with HPAF-II CDX tumors, female SCID Beige mice 6–8 weeks old from Beijing Vital River Laboratory Animal Technology Co. were used. For study with KP-4 CDX tumors, female BALB/c nude mice 6–8 weeks old purchased from Shanghai Sino-British SIPPR/BK Lab Animal Co. were used. For studies with KPC/Y GEMM-derived models, female C57BL/6J mice 6–8 weeks old were used obtained from The Jackson Laboratory. For study with MIA Paca-2 CDX tumors, 8–12-week-old female NSG mice were purchased from The Jackson Laboratory.

### In vivo model development

Capan-1 tumor cells (ATCC-HTB-79) were maintained in vitro as a monolayer culture in IMDM supplemented with 20% heat-inactivated fetal bovine serum, at 37 °C in an atmosphere of 5% CO_2_ in air and routinely subcultured twice weekly by trypsin–EDTA treatment. Cells in the exponential growth phase were collected and counted for tumor inoculation. Each mouse was inoculated subcutaneously on the right flank with 5 × 10^6^ Capan-1 tumor cells in a 0.2-ml PBS/Matrigel mixture (1:1 ratio) to initiate tumor development. For efficacy studies, treatments were initiated when the average tumor volume reached 146 mm^3^ (Fig. 3e) or 167 mm^3^ (Fig. 4c) Animals were assigned to groups using Studylog-based randomization software which performed stratified randomization based on tumor volumes. For the pharmacodynamic (PD) study, treatments were initiated when the average tumor volume reached 400–600 mm^3^.

The HPAF-II tumor cells (ATCC, CRL-1997) were maintained in vitro as a monolayer culture in EMEM supplemented with 10% fetal bovine serum and 1% antibiotic–antimycotic at 37 °C in an atmosphere of 5% CO_2_ in air and routinely subcultured twice weekly. Cells growing in an exponential growth phase were collected, counted for tumor inoculation, and inoculated subcutaneously at the right upper flank with 3 × 10^6^ tumor cells in 0.2 ml of PBS with Matrigel (1:1) per animal. Treatments were initiated at the time of randomization when the mean tumor volume reached 155 mm^3^.

The Capan-1 tumor model resistant to daraxonrasib (25 mg kg^−1^ po qd) was established through serial passaging with daraxonrasib treatment in vivo (Extended Data Fig. 3). For each round of tumor passaging, recipient mice were dosed with daraxonrasib at 25 mg kg^−1^ for 3 days before the implantation of tumor fragments, followed by continued daily dosing of daraxonrasib until tumor volumes >600 mm^3^ at which time these were collected and re-passaged. Daraxonrasib-resistant tumors from passage 3 (P3) and passage 5 (P5) were used for PD and efficacy studies, respectively. Each mouse was inoculated subcutaneously in the right upper flank with daraxonrasib-resistant Capan-1 tumor slices (~30 mm^3^) to initiate tumor development. For the PD study, treatments were initiated when the average tumor volume reached 400–600 mm^3^. For the efficacy study, treatments were initiated when the average tumor volume reached 163–164 mm^3^. The animals were assigned to groups (*n* = 6) using a Studylog-based randomization software as above. Similarly, the HPAF-II tumor model resistant to RMC-7977 (10 mg kg^−1^ po qd) was established through serial passaged with RMC-7977 treatment in vivo (Extended Data Fig. 3).

HPAC tumor cells (ATCC CRL-2119) were maintained as a monolayer culture in RPMI 1640 medium supplemented with 10% fetal bovine serum and 1% penicillin–streptomycin at 37 °C in a humidified atmosphere containing 5% CO_2_. The culture medium was refreshed every 2–3 days, and cells were routinely subcultured at 80–90% confluence using trypsin–EDTA, with passages limited to a maximum of 4–5. Cells in the exponential growth phase were collected, counted for tumor inoculation and each mouse was inoculated subcutaneously in the right flank with 1 × 10^7^ cells suspended in 100 µl of a 1:1 mixture of RPMI 1640 and Matrigel to establish tumors. Treatment commenced upon randomization once the mean tumor volume reached 139 mm^3^. For in vivo RAS(ON) inhibitor-resistant model derivation, HPAC xenograft tumors that emerged after initial tumor regressions in response to RMC-9945 (the RAS(ON) G12D-selective inhibitor tool representative of the investigational agent zoldonrasib^26^), wherein we observed amplification of mutant *KRAS*^*G12D*^ in vivo (Fig. 3d and Extended Data Fig. 5b). We then established a HPAC tumor-derived cell line from these RMC-9945-resistant tumors, which was maintained under constant RAS(ON) inhibitor pressure.

KPC/Y GEMM-derived tumor cells 6694c2, 2838c3, 6499c4 and 6419c5, were obtained from Kerafast and are referred to as KPCYc2, KPCYC3, KPCYc4 and KPCYc5 thereafter. Cells were maintained as a monolayer culture in DMEM supplemented with 10% fetal bovine serum and 1% penicillin–streptomycin at 37 °C in a humidified atmosphere containing 5% CO_2_. The culture medium was refreshed every 2–3 days, and cells were routinely subcultured at 80% confluence using trypsin–EDTA, with passages limited to a maximum of 3–4. Cells in the exponential growth phase were collected, counted for tumor inoculation and each mouse was inoculated subcutaneously in the right flank with 3 × 10^5^ cells resuspended in serum-free DMEM. For efficacy studies, treatment commenced upon randomization once the mean tumor volume reached 100–120 mm^3^.

For studies performed in MIA Paca-2 (ATCC CRL-1420) CDX tumors, WU-0002, WU-0022 PDX tumors, 2–5 million PDAC cells or 5 x 5 x 5-mm chunks of cryopreserved PDXs were mixed 1:1 (v/v) with Matrigel matrix (Corning) and inoculated into both flanks of 8–12-week-old female NSG mice (purchased from The Jackson Laboratory) by needle injection or small incision subcutaneously at the flanks of each mouse. Treatments were initiated when the tumors reached ~100 mm^3^ in volume.

KP-4 tumor cells (RIKEN-RCB1005) were maintained in vitro as a monolayer culture in IMDM supplemented with 20% heat-inactivated fetal bovine serum at 37 °C in an atmosphere of 5% CO_2_ in air and routinely subcultured twice weekly by trypsin–EDTA treatment. The cells growing in an exponential growth phase were collected, counted for tumor inoculation and each mouse will be inoculated subcutaneously at the right upper flank with KP-4 tumor cells (5 × 10^6^) in 0.2 ml of PBS with Matrigel (1:1) for tumor development. Treatments were initiated at the time of randomization when the mean tumor volume reached 141 mm^3^ (Fig. 4e) or 160 mm^3^ (Fig. 4f).

The PSN-1 tumor cells (ATCC CRL-3211) were cultured as a monolayer culture in RPMI 1640 medium supplemented with 10% fetal bovine serum at 37 °C in an atmosphere of 5% CO_2_ in air. The medium was renewed every 2–3 days and tumor cells were routinely subcultured at a confluence of 80–90% by trypsin–EDTA, not exceeding 4–5 passages. Cells in the exponential growth phase were collected, counted for tumor inoculation and each mouse was inoculated subcutaneously on the right flank with 5 × 10^6^ PSN-1 tumor cells in 100 µl of an RPMI 1640/Matrigel mixture (1:1 ratio) to initiate tumor development. Treatments were initiated at the time of randomization when the mean tumor volume reached 171 mm^3^.

### In vivo treatment

For tumor growth evaluation studies, tumor-bearing animals were randomized and grouped for treatments (*n* = 4–10 per group). The vehicle, daraxonrasib, zoldonrasib, RMC-7977, RMC-9945, azenosertib or IAG933 at indicated doses were administered daily or at indicated dosing schedule via oral gavage with a 10 ml kg^−1^ dosing volume. Daraxonrasib (25 mg kg^−1^ po qd) combined with zoldonrasib (100 mg kg^−1^ po qd) was well tolerated in syngeneic models using immunocompetent C57BL/6J mice; however, this same dosing regimen was not tolerated in xenograft models in immunodeficient mice and a reduced daraxonrasib dose (10 mg kg^−1^ po qd) was used in combination with zoldonrasib (100 mg kg^−1^ po qd) to ensure tolerability. T-DXd was purchased from Gruechem and dosed at 4 mg kg^−1^ via i.v. injection weekly for studies performed in Capan-1 and daraxonrasib-resistant Capan-1 tumors. DS-8201a used in studies of MIA Paca-2, WU-0002, WU-0022 and Pa01C tumors was purchased from the Washington University Siteman Cancer Center Pharmacy and approved for use under Institutional Biosafety Committee protocol 16701 v.3 and dosed at 10 mg kg^−1^ via i.v. injection weekly. Azenosertib and IAG933 were purchased from Gruechem. Animals were euthanized as previously described^8^. Snap-frozen and formalin-fixed samples were collected following the same protocols as previously described^28,44^. RMC-9945, zoldonrasib, daraxonrasib, azenosertib, IAG933 and the combination of daraxonrasib plus azenosertib or IAG933 were formulated in 10:20:10:60 (%v/v/v/v) (DMSO:PEG400:Solutol HS15:water). T-DXd was formulated in sterile saline. For studies performed in MIA Paca-2, WU-0002, WU-0022 and Pa01C tumors, daraxonrasib was formulated in vehicle consisting of 5% DMSO (CAS no. 67-68-5; Fisher Scientific).

### In vivo study data analysis

In tumor volume plots, the average tumor volume of each group in CDX models was plotted post-implantation, while the *x* axis started on the first day of treatment in daraxonrasib-resistant Capan-1, KPC, WU-0002, WU-0022, Pa01C and MIA Paca-2 tumor models. Control and treatment groups were compared by two-way repeated-measures ANOVA (Dunnett’s multiple comparisons test) on the last measurement day of the control group. Tumor diameter was measured in two dimensions using a digital caliper and mice in the study were weighed twice per week. The tumor volume in mm^3^ was calculated based on the formula: volume = (length × (width)^2^)/2. An individual tumor was deemed to have regressed (R) if it showed >10% reduction in tumor volume from the initial volume and deemed to have CR if it showed ≥80% reduction in tumor volume from the initial volume on a given day. The percentage change in body weight for each animal on a given day was determined as ((body weight on test article administration end date/body weight on test article administration start date) − 1) × 100.

### In vitro resistant cell line establishment and characterization

HPAC CDX tumors relapsed on RMC-9945 (RAS(ON) G12D-selective inhibitor) were collected and dissociated using the Tumor Dissociation kit (human, cat. no. 130-095-929) in conjunction with a gentleMACS Octo Dissociator to isolate tumor cells. These isolated tumor cells were cultured in vitro under inhibitor pressure until they demonstrated sustained growth in the presence of 100 nM RMC-9945 (Extended Data Fig. 3 provides a schematic illustration). The resistant cells were characterized to confirm cell identity, purity, and KRAS G12D/WT CN via digital PCR, along with whole-exome sequencing with ~100× coverage at Azenta.

### Droplet digital PCR and digital PCR

Genomic DNA (gDNA) was extracted from at least 20 mg of tumor tissue using a QIAamp DNA Mini kit (QIAGEN, cat. no. 51304). Then, 20 ng of gDNA was included for digital PCR (dPCR) using the Naica system multiplex digital PCR (Stilla) or Thermo Fisher QuantStudio Absolute Q Digital PCR system following the manufacturer’s protocol. Probes and primers for the following genes were included in the multiplexed droplet dPCR of PSN-1 end-of-study tumors: *KRAS*^*WT*^ (dPCR Mutation Detection Assay KRAS WT for p.G12C, Human (Apexbio, AA100902-WT)), *KRAS*^*G12R*^ (dPCR Mutation Detection Assay KRAS Mutant for p.G12R, Human (Apexbio, AA100904-MU)) and *RPP30* (*RPP30* probe, 5′-Cy5-TTCTGACCTGAAGGCTCTGCGC-3′; *RPP30* primer F, 5′-TCAGCATGGCGGTGTTT-3′; *RPP30* primer R, 5′-TGGCATGAGGTTGGCCA-3′); MYC (*MYC* probe FAM-TGCTGCTGCTGCTGGTAGAAGTTC; *MYC* primer F, 5′-GGTGCAGCCGTATTTCTACTG -3′; *MYC* primer R, 5′-AGCAGCTCGAATTTCTTCCAGA-3′). The results were normalized to *RPP30* for CN assessment.

For the dPCR analysis of HPAC resistant cells, DNA was extracted from ~2 million cells using the DNeasy Blood & Tissue kit (QIAGEN, cat. no. 69504). Then, 9 ng of gDNA was included for dPCR using the Thermo Fisher QuantStudio Absolute Q Digital PCR system following the manufacturer’s protocol. The following kits were used for KRAS WT, KRAS G12D and MYC detection and CN evaluation: Absolute Q Liquid Biopsy dPCR Assays, (Thermo Fisher Scientific, A53732, assay ID DGPRJZK), RNaseP (Thermo Fisher Scientific, 4485714) and MYC (Thermo Fisher Scientific, assay ID Hs00292858_cn). The results were normalized to RNaseP for CN assessment.

### Immunohistochemistry

Tumor fragments collected from mice after the indicated treatments were fixed in 10% neutral buffered formalin at room temperature for up to 24 h, stored at 70% ethanol and then processed into paraffin blocks. All staining was performed on 4-µm tissue sections.

For IHC staining of HER2 in tumor tissues collected from Capan-1 and daraxonrasib-resistant Capan-1 model, formalin-fixed paraffin-embedded (FFPE) sections were stained on a BOND-III Automated IHC Stainer using the manufacturer’s recommended settings. Anti-EpCAM rabbit monoclonal antibody (Cell Signaling Technology, cat. no. 14452, clone: D9S3P) was used at 1:400 dilution with EDTA-based pH 9 epitope retrieval solution. Anti-HER2 rabbit monoclonal antibody (Gene Tech, cat. no. GT224507) was used with EDTA-based pH 9 epitope retrieval solution. A BOND IHC Polymer detection kit (Leica, DS9800) was used to detect rabbit primary antibodies. DAB-stained slides were scanned and digitized with a 3DHISTECH Panoramic MIDI whole-slide scanner at ×200 magnification.

To stain tissues from KPCY allograft models, a Biocare IntelliPATH automation system was used. Anti-phospho-ERK1/2 rabbit monoclonal antibody (Cell Signaling Technology cat. no. 4370, clone D13.14.4E) was used at 1:200 dilution and anti-panCK rabbit monoclonal antibody (Abcam, cat. no. ab308262, clone EPR28285-45) was used at 1:5,000 dilution with citrate-based pH 6.2 Heat-Induced Epitope Retrieval (Biocare, cat. no. DV2004). Primary antibodies were detected with the MACH4-HRP-polymer Detection System (Biocare, MRH534). Stained slides were scanned and digitized with a TissueScope LE (Huron Digital Pathology) whole-slide scanner at ×20 magnification. Image analysis was performed using HALO software from Indica Labs.

For IHC staining of RAS in HPAC control tumors and tumors relapsed on RMC-9945 treatment, FFPE sections were stained on the Biocare IntelliPATH automated staining platform using the manufacturer’s recommended settings. Anti-human Ras rabbit monoclonal antibody (Cell Signaling, cat. no. 67648, clone E8N8L, lot no. 2, 44 μg ml^−1^) was used at 1:400 dilution (0.11 μg ml^−1^) with citrate-based pH 6.2 Heat-Induced Epitope Retrieval (Biocare, cat. no. DV2004); an isotype control (rabbit IgG) was used under the same conditions. Stained slides were digitized with a Leica ScanScope AT whole-slide scanner at ×200 magnification.

### Whole-slide image analysis

panCK staining was used to identify epithelial cells. First, the area to be analyzed was delineated, excluding necrotic regions. The random forest Tissue Classifier from the HALO Image Analysis package was used to identify the tumor compartment. Three classes were created: glass, tumor (panCK-positive) and stroma. The tumor class mark was then copied onto the serial sections stained with markers to perform the image analysis on only the tumor compartment.

Quantification of pERK was performed with the HALO Image Analysis software from Indica Labs using the Area Quantification module. The analysis was performed only on the tumor compartment copied from the panCK slide. The software was tuned to detect positive DAB staining (brown color). The percentage of area positivity was chosen to represent the results (area positive for brown/total area) and subjected to statistical analysis using GraphPad Prism (Dunnett multiple comparisons test).

### Western blotting

Tumor tissues were dissected, cut into 35–50-mg fragments, snap frozen in liquid nitrogen and stored at −80 °C. Frozen tumor fragments were homogenized in a tissue grinder (Scientz, Scientz-48) with NP-40 cell lysis buffer (Invitrogen, FNN0021). Cells cultured in vitro were washed twice with ice-cold PBS and lysed with MSD Tris Lysis Buffer (MSD, R60TX-2), and scraped and collected before centrifugation. All lysis buffers were supplemented with protease and phosphatase inhibitors (Thermo, 78444). Lysates were centrifuged at 14,000 × rpm for 10 min at 4 °C. The protein concentrations in supernatant were measured by PierceTM BCA Protein Assay kit (Thermo, 23225). Then all the lysates with equal quantities of protein were denatured at 95 °C for 10 min after adding LDS sample buffer (Thermo, NP0008) and a reducing agent (Thermo, NP0009). Samples were resolved on 4–12% Bis-Tris gels (Thermo, WG1403BOX) and western blotting was performed following the standard protocol.

The following primary antibodies were used: anti-KRAS (Santa-Cruz, sc-30), anti-pan RAS(Abcam, ab108602), anti-Ras (G12D mutant) (Thermo Fisher Scientific, MA5-36256), anti-HER2 (Cell Signaling Technology, 2242), anti-phospho-p44/42 MAPK (ERK1/2) T202/Y204 (Cell Signaling Technology, 4370), anti-p44/42 (ERK1/2) (Cell Signaling Technology, 4696 or 4695), anti-phospho-MEK1/2 Ser217/221 (Cell Signaling Technology, 9154), anti-MEK1/2 (Cell Signaling Technology, 9126), anti-Flag (Abcam, ab205606), anti-Vinculin (Cell Signaling Technology, 13901), anti-β-actin (Cell Signaling Technology, 4967) and anti-β-tubulin (Cell Signaling Technology, 86298). IRDye 800CW goat anti-mouse IgG (H + L) (Licor, 926-32210) and goat anti-rabbit IgG (H + L) HRP-conjugated (Thermo Fisher Scientific, 31462) were used as secondary antibodies.

### 2D cellular viability assays

Cells were plated in tissue culture-treated 384-well plates and incubated overnight. The following day, ten serial dilutions of compound were prepared in the growth medium. Cells were treated with compound or DMSO (0.1% v/v) for 5 days (T5). Luminescence was detected before treatment (T0) and after treatment (T5) using the Envision Plate Reader (PerkinElmer). Then, 25 μl of CellTiter-Glo 2.0 reagent (G9243, Promega) was added to each well. Plates were shaken at 450 rpm for 2 min at room temperature and then incubated for an additional 10 min without shaking at room temperature and protected from light. Luminescence signal from the CellTiter-Glo 2.0 reagent was normalized to DMSO treated wells (% DMSO = (lum_treated_(T5)-lum_DMSO_ (T0)/(lum_DMSO_ (T5)-lum_DMSO_ (T0) × 100). Data were plotted as a function of inhibitor concentration, and a four-parameter sigmoidal concentration response model was fitted.

### Cell line engineering

*KRAS*^*G12R*^ overexpression cell line experiments were performed at Pharmaron. The plasmid encoding Tet-On 3G and TRE3GS-inducible Flag-tagged *KRAS*^*G12R*^ was synthesized and packaged into lentivirus at Pharmaron. Lentivirus transductions were performed with addition of Polybrene (10 µg ml^−1^). Lentivirus particles were added to HuP-T3 cells and incubated for 24 h after which lentivirus particles were added again to improve transduction efficiency. Then, puromycin (2 µg ml^−1^) was added in the culture medium to maintain selective pressure. Expression of the transgene was induced by adding doxycycline (0.1–1 µg ml^−1^) for at least 24 h.

AP009 *KRAS*^*G12D*^ overexpression cell line: AP009 Kras^G12D/wt^;Trp53^null/null^ cell line was generated from a LSL-Kras^G12D/wt^ mouse and maintained in adherent culture as described previously^45^. AP009c2 clonal cell line was isolated from parental AP009 cells with single-cell sorted by FACS. Expanded clones were confirmed with Kras^G12D/wt^;Trp53^null/null^ genotype by sequencing. To generate clonal cell lines with mouse Kras overexpression, AP009c2 cells were transduced with lentivirus expressing mutant *Kras* G12D cDNA at optimized multiplicity of infection. On the third day after lentiviral infection cells were single-cell sorted by FACS into four 96 well plates. Expanded clones were verified by PCR and sequenced for mutant *KRAS*^*G12D*^ transgenes. Positive clones with mutant *KRAS*^*G12D*^ overexpression were evaluated by quantitative PCR using forward primer 5′-GGACTCTGAAGATGTGCCTATGGT-3′, reverse primer 5′-GCCTGTTTCGTGTCTACTGTTC-3′ and probe 5′-CCTGGTAGGGAATAAGTGTGATTTGCC-3′.

The open reading frame of mouse *Kras* cDNA (NM_021284.7) with *KRAS*^*G12D*^ mutation was cloned into the custom lentiviral cDNA expression plasmid pRR-mCMV-cDNA (Cellecta). Lentivirus was generated by packaging plasmids into VSV-G pseudotyped viral particles using Cellecta’s Ready-to-use Lentiviral Packaging Plasmid Mix (cat. no. CPCP-K2A). The titer of lentiviral packaged constructs provided by Cellecta is functionally determined by transduction of 293T cells.

#### Flow cytometry

To assess surface HER2 expression, cells were stained with 4 µg ml^−1^ Alexa Fluor 647-conjugated anti-human HER2 antibody (BioLegend, cat. no. 324412) and analyzed using FACScan 5. Data were processed and quantified using FlowJo software (BD Biosciences).

### Bioinformatics analysis (preclinical studies)

Whole-exome sequencing analysis was performed to ascertain gene mutations. For parental cell line-derived xenograft models, gene mutation data were obtained from the 23Q4 release of the DepMap Data Portal. DNA mutation calling was accomplished with TNSeq using the hg38 version of the human genome.

### Deep mutational scanning screen

DMS data were processed as previously indicated. In brief, reads were aligned to the KRAS^G12C^ reference sequence and collapsed to reads per amino acid at each position and for each amino acid substitution included in the library. Substitution not included in the library and likely PCR artifacts were removed and not considered for downstream analyses. The abundance of each allelic variant was calculated as the fraction of its read compared to the total library-wide reads in each sample independently. Variant abundances were defined as the log_2_ fold change (FC) between 6-day-treated time points (RMC-7977 or DMSO) and the initial variants representation at day 0 (ETP) and were computed by applying a moderated *t*-test as implemented in the R package limma (v.3.54.2). We defined a ‘RASi resistance’ and a fitness threshold as the average log_2_FC + 2 × s.d. in the RMC-7977-day 6 versus ETP and DMSO day 6 versus ETP comparisons, respectively.

Bulk-RNA sequencing analyses were performed to ascertain gene expression. Transcript-level quantification was performed using Salmon (v.1.0.8) in quasi-mapping mode. Reference transcriptome used for quantification was derived from the mouse genome (GRCm38). The transcript-level counts generated by Salmon were imported into R, and genes with low expression across the samples were filtered out before downstream analysis. Normalization of gene-level counts was performed using the trimmed mean of M-values method, which was used to calculate the log-transformed counts per million values using the CPM function from the edgeR package (v.4.2.2).

### Mapping phenotypic data onto protein structures

Functional data from the DMS screen was mapped onto the structure of KRAS^G12C^ bound to GNP and in complex with RMC-7977 and PPIA (PDB: 8BTK). Graphical display was carried out using the ‘define by attribute’ and ‘render attribute’ functions implemented in Chimera X. For each residue of KRAS^G12C^, the average log_2_FC of all substitutions affecting the same residue.

### FISH

Fluorescent in situ hybridization was performed on either methanol acetic acid fixed in vitro cells or FFPE tumor sections using dual labeled KRAS/centromere 12 probes from Empire Genomics (KRAS-CON12-REGR).

Metaphase cells were collected from cell lines treated with 0.2 μg ml^−1^ Colcemid (GIBCO cat. no. 15210-040) for 4 h, trypsinized and treated with 0.075 M KCl for 20 min at 37 °C before being fixed three times in 75% methanol/25% acetic acid. Fixed cells were stored at −20 °C and then metaphase spreads were prepared on glass slides and air dried. For hybridization, slides were dehydrated in a series of ethanol (70%, 85% and 100%) for 2 min each and air dried. Probes were mixed with hybridization buffer and applied to fixed metaphase slides, covered with a 22 × 22 mm cover-glass and sealed with Cytobond (SciGene cat. no. 2020-00-1). Slides were denatured at 73 °C for 3 min and hybridized overnight at 37 °C in a Cytobrite Plus slide incubation system (SciGene) and then washed with 0.4× SSC (saline sodium citrate) + 0.3% IGEPAL, pH 7 buffer (SciGene) at 73 °C for 2 min followed by another washing with 2× SSC + 0.1% IGEPAL (SciGene) at room temperature for 2 min. Slides were quickly rinsed in water and then air dried. Cells were stained with 4,6-diamidino-2-phenylindole (DAPI) with antifade and imaged with a ×40 immersion objective on a Leica DMi8 microscope.

FFPE tumor sections were dewaxed and then treated with the Abbott FFPE Pretreatment kit to prepare the fixed DNA for hybridization. Sections were incubated in 0.2 N HCl for 20 min at room temperature, followed by incubation in sodium thiocyanate for 30 min at 80 °C and digestion with protease for 30 min at 37 °C. In situ hybridizations for FFPE sections were as described for in vitro cells, except that sections were denatured at 78 °C for 7 min.

### Reporting summary

Further information on research design is available in the Nature Portfolio Reporting Summary linked to this article.

## Online content

Any methods, additional references, Nature Portfolio reporting summaries, source data, extended data, supplementary information, acknowledgements, peer review information; details of author contributions and competing interests; and statements of data and code availability are available at 10.1038/s41591-026-04537-w.

## Supplementary information

Reporting Summary

Peer Review File

Supplementary Tables 1–5 Supplementary Table 1. Acquired genomic alterations detected in paired baseline and EOT ctDNA profiled from 44 patients treated with 160–300-mg doses of daily daraxonrasib. Genomic alterations include short variants and copy number variants and changes in ctDNA RAS VAF detected at pretreatment (Pre), early on treatment (either cycle 2 day 1 or cycle 3 day 1) (On), and EOT (End) with daraxonrasib. Supplementary Table 2. Acquired genomic alterations detected in paired baseline and EOT ctDNA profiled from 13 patients treated with 120 mg or lower doses of daily daraxonrasib. Genomic alterations include short variants and copy number variants and changes in ctDNA RAS VAF detected at pretreatment (Pre), early on treatment (either cycle 2 day 1 or cycle 3 day 1) (On), and EOT (End) with daraxonrasib. Supplementary Table 3. Summary of 16 patients with acquired KRAS amplification. *Mutant KRAS allele amplification expected to increase ratio of mutant KRAS VAF relative to other tumor mutation VAF. Method is exploratory, and allele amplification is not confirmed. Values above 1 are putative amplification of RAS mutant allele and below 1 are putative amplification of RAS WT. VAFs not adjusted for CN. ^#^Amplification copy number is observed copies in total cell-free DNA and may underestimate tumor copy number. Supplementary Table 4. Baseline genomic alterations from 57 patients treated with daily dose (20–300 mg) of daraxonrasib. Genomic alterations (short variants and copy number variants) in pretreatment ctDNA from 57 patients profiled in this study. Supplementary Table 5. Summary of preclinical models in this study.

## Source data

Source Data Fig. 1 Statistical source data.

Source Data Fig. 2 Statistical source data and CT scan images.

Source Data Fig. 3 Statistical source data, unprocessed blots.

Source Data Fig. 4 Inhibitor response source data.

Source Data Fig. 5 Inhibitor response source data.

Source Data Extended Data Fig. 1 Statistical source data for changes in ctDNA.

Source Data Extended Data Fig. 2 CT scan images.

Source Data Extended Data Fig. 3 DMS heatmap, scatter-plot, residue Fisher’s test, structural mapping.

Source Data Extended Data Fig. 5 Statistical source data.

Source Data Extended Data Fig. 6 Source data of mice body weight.

Source Data Extended Data Fig. 7 Source data of mice body weight.

Source Data Extended Data Fig. 8 Statistical source data for matrix combo response, unprocessed blots.

Source Data Extended Data Fig. 9 Statistical source data.

Statistical source data.

## Data Availability

All data supporting the findings of this study are available within the article and its Supplementary Information. DNA sequencing data from the parental and resistant HPAC xenograft models have been deposited in the NCBI Gene Expression Omnibus under accession number GSE335219. Clinical and translational data were generated as part of the phase 1/2 study NCT05379985 and available as stated in 10.1056/NEJMoa2505783. Source data are provided with this paper.
