# Supplementary material for: Acquired resistance to the RAS(ON) multi-selective inhibitor daraxonrasib guides rational combination therapy strategies in pancreatic cancer
Source: Nat Med. 2026 Aug 11;32(8):2865–77. doi: 10.1038/s41591-026-04537-w (PMC13472880; doi:10.1038/s41591-026-04537-w)
Supplement: Supplementary file 1 [file 41591_2026_4537_MOESM1_ESM.pdf]

nature portfolio

Corresponding author(s): Kevin K. Lin, Andrew J. Aguirre, Mallika Singh

Last updated by author(s): Jun 18, 2026

Reporting Summary

Nature Portfolio wishes to improve the reproducibility of the work that we publish. This form provides structure for consistency and transparency in reporting. For further information on Nature Portfolio policies, see our Editorial Policies and the Editorial Policy Checklist.

Statistics

For all statistical analyses, confirm that the following items are present in the figure legend, table legend, main text, or Methods section.

n/a	Confirmed	
☐	☒	The exact sample size (*n*) for each experimental group/condition, given as a discrete number and unit of measurement
☐	☒	A statement on whether measurements were taken from distinct samples or whether the same sample was measured repeatedly
☐	☒	The statistical test(s) used AND whether they are one- or two-sided<br>*Only common tests should be described solely by name; describe more complex techniques in the Methods section.*
☐	☒	A description of all covariates tested
☒	☐	A description of any assumptions or corrections, such as tests of normality and adjustment for multiple comparisons
☒	☐	A full description of the statistical parameters including central tendency (e.g. means) or other basic estimates (e.g. regression coefficient) AND variation (e.g. standard deviation) or associated estimates of uncertainty (e.g. confidence intervals)
☒	☐	For null hypothesis testing, the test statistic (e.g. *F*, *t*, *r*) with confidence intervals, effect sizes, degrees of freedom and *P* value noted<br>*Give P values as exact values whenever suitable.*
☒	☐	For Bayesian analysis, information on the choice of priors and Markov chain Monte Carlo settings
☒	☐	For hierarchical and complex designs, identification of the appropriate level for tests and full reporting of outcomes
☒	☐	Estimates of effect sizes (e.g. Cohen's *d*, Pearson's *r*), indicating how they were calculated

Our web collection on statistics for biologists contains articles on many of the points above.

Software and code

Policy information about availability of computer code

Data collection	Clinical data were collected using validated electronic data capture systems in accordance with the study protocol and Good Clinical Practice guidelines. Flow cytometry data were acquired using FACScan 5 instrumentation (BD Biosciences). Histology images were acquired using 3DHISTECH Panoramic MIDI, TissueScope LE (Huron Digital Pathology), Leica ScanScope AT, and Leica DMi8 imaging platforms as described in the Methods.
Data analysis	Flow cytometry data were analyzed using FlowJo (BD Biosciences). Whole-slide image analyses were performed using HALO software (Indica Labs). Statistical analyses and graphical visualization were performed using GraphPad Prism. Whole-exome sequencing, bulk RNA sequencing, and deep mutational scanning analyses were performed using established bioinformatics pipelines, including Salmon (v1.0.8), edgeR (v4.2.2), limma (v3.54.2), and custom analyses implemented in R. Structural visualization and mapping of mutational data onto protein structures were performed using ChimeraX. No custom code central to the conclusions of the study was developed that requires public deposition.Code availability: No custom software was developed for this study. Analyses were performed using commercially available software and established bioinformatics tools as described in the Methods.

For manuscripts utilizing custom algorithms or software that are central to the research but not yet described in published literature, software must be made available to editors and reviewers. We strongly encourage code deposition in a community repository (e.g. GitHub). See the Nature Portfolio guidelines for submitting code & software for further information.

Data

Policy information about availability of data

All manuscripts must include a data availability statement. This statement should provide the following information, where applicable:

- Accession codes, unique identifiers, or web links for publicly available datasets
- A description of any restrictions on data availability
- For clinical datasets or third party data, please ensure that the statement adheres to our policy

All data supporting the findings of this study are available within the Article and its Supplementary Information. DNA sequencing data from the parental and resistant HPAC xenograft models have been deposited in the NCBI Gene Expression Omnibus (GEO) under accession number GSE335219. Clinical and translational data were generated as part of the Phase 1/2 study NCT05379985 and DOI: 10.1056/NEJMoa2505783.

Research involving human participants, their data, or biological material

Policy information about studies with human participants or human data. See also policy information about sex, gender (identity/presentation), and sexual orientation and race, ethnicity and racism.

Reporting on sex and gender	Sex was collected as a demographic variable at enrollment and reported according to patient self-identification. No analyses were performed to evaluate differences in treatment response or acquired resistance mechanisms by sex or gender because the study was not designed or powered for such comparisons.
Reporting on race, ethnicity, or other socially relevant groupings	Race and ethnicity were collected at enrollment according to site-specific reporting procedures and patient self-identification. Race and ethnicity were not used as inclusion or exclusion criteria and were not analyzed as variables associated with clinical outcomes or resistance mechanisms because the study was not designed or powered for such comparisons.
Population characteristics	The study included adult patients with previously treated metastatic RAS-mutant pancreatic ductal adenocarcinoma enrolled in the Phase 1/2 clinical trial (NCT05379985). Eligibility criteria, baseline demographic characteristics, disease characteristics, prior therapies, and clinical outcomes have been previously reported and are summarized in the associated clinical publication. The translational analyses presented herein were performed using paired pre-treatment and end-of-treatment circulating tumor DNA samples from 44 patients who achieved clinical benefit on daraxonrasib followed by disease progression.
Recruitment	Participants were recruited prospectively from participating clinical trial sites as part of the Phase 1/2 study of daraxonrasib in patients with advanced RAS-mutant solid tumors. All participants provided written informed consent prior to enrollment. No financial incentives were provided for participation beyond study-related procedures described in the protocol.
Ethics oversight	The clinical study was conducted in accordance with the principles of the Declaration of Helsinki, International Council for Harmonisation Good Clinical Practice guidelines, and applicable local regulations. The protocol and all amendments were approved by the institutional review board or ethics committee at each participating site. All participants provided written informed consent before enrollment. The institutional review boards overseeing the study included Advarra, Dana-Farber Cancer Institute, WCG, Salus IRB, Johns Hopkins Medicine, Memorial Sloan Kettering Cancer Center, Mary Crowley Medical Research Center, UCLA Office of Human Research Protection Program, NYU Grossman School of Medicine, and Columbia University Irving Medical Center.

Note that full information on the approval of the study protocol must also be provided in the manuscript.

Field-specific reporting

Please select the one below that is the best fit for your research. If you are not sure, read the appropriate sections before making your selection.

☒ Life sciences ☐ Behavioural & social sciences ☐ Ecological, evolutionary & environmental sciences

For a reference copy of the document with all sections, see nature.com/documents/nr-reporting-summary-flat.pdf

Life sciences study design

All studies must disclose on these points even when the disclosure is negative.

Sample size	For the clinical study, sample size was determined by the Phase 1/2 trial design and patient availability and was not based on formal statistical power calculations for the translational analyses presented herein. For preclinical studies, sample sizes were selected based on prior experience with similar models, expected effect sizes, study objectives, and practical considerations. No formal statistical methods were used to predetermine sample size.
Data exclusions	No data were excluded from the analyses unless otherwise specified. Animals were removed only if they met predefined protocol-specified criteria for euthanasia or study discontinuation. All available clinical samples meeting prespecified inclusion criteria for paired analyses were included.
Replication	Key in vitro experiments were independently repeated at least twice with comparable results. For cellular viability assays, representative

Replication	experiments are shown and technical duplicates were included within each experiment. Findings were further corroborated across multiple independent preclinical models, including engineered cell lines, organoid-derived models, xenografts, patient-derived xenografts, and genetically engineered mouse model-derived tumors. Clinical observations were independently supported by orthogonal preclinical studies.
Randomization	The clinical study was a single-arm Phase 1/2 trial and participants were not randomized. For in vivo preclinical studies, animals were randomized to treatment groups after tumor establishment using Studylog-based randomization software or equivalent study-site procedures, typically based on tumor volume stratification. Investigators were not blinded to treatment allocation unless otherwise indicated.
Blinding	The clinical study was an open-label, single-arm Phase 1/2 trial and was therefore not blinded. For preclinical studies, investigators were not blinded to treatment allocation during experiments or outcome assessment unless otherwise indicated. No blinding was used for data collection or analysis.

Reporting for specific materials, systems and methods

We require information from authors about some types of materials, experimental systems and methods used in many studies. Here, indicate whether each material, system or method listed is relevant to your study. If you are not sure if a list item applies to your research, read the appropriate section before selecting a response.

Materials & experimental systems

n/a	Involved in the study
☐	☒ Antibodies
☐	☒ Eukaryotic cell lines
☒	☐ Palaeontology and archaeology
☐	☒ Animals and other organisms
☐	☒ Clinical data
☒	☐ Dual use research of concern
☒	☐ Plants

Methods

n/a	Involved in the study
☒	☐ ChIP-seq
☐	☒ Flow cytometry
☒	☐ MRI-based neuroimaging

Antibodies

Antibodies used	The following antibodies were used in the studies described herein. For immunohistochemistry (IHC), anti-EpCAM (Cell Signaling Technology, clone D9S3P, cat. no. 14452), anti-HER2 (Gene Tech, cat. no. GT224507), anti-phospho-ERK1/2 (Thr202/Tyr204; Cell Signaling Technology, clone D13.14.4E, cat. no. 4370), anti-pan-cytokeratin (Abcam, clone EPR28285-45, cat. no. ab308262), and anti-human RAS (Cell Signaling Technology, clone E8N8L, cat. no. 67648) antibodies were used, along with a rabbit IgG isotype control. For flow cytometry, surface HER2 expression was assessed using an Alexa Fluor 647-conjugated anti-human HER2 antibody (BioLegend, cat. no. 324412). For western blotting, antibodies against KRAS (Santa Cruz Biotechnology, cat. no. sc-30), pan-RAS (Abcam, cat. no. ab108602), KRAS G12D (Thermo Fisher Scientific, cat. no. MA5-36256), HER2 (Cell Signaling Technology, cat. no. 2242), phospho-ERK1/2 (Thr202/Tyr204; Cell Signaling Technology, cat. no. 4370), ERK1/2 (Cell Signaling Technology, cat. nos. 4695 and 4696), phospho-MEK1/2 (Ser217/221; Cell Signaling Technology, cat. no. 9154), MEK1/2 (Cell Signaling Technology, cat. no. 9126), FLAG (Abcam, cat. no. ab205606), vinculin (Cell Signaling Technology, cat. no. 13901), β-actin (Cell Signaling Technology, cat. no. 4967), and β-tubulin (Cell Signaling Technology, cat. no. 86298) were used. Secondary antibodies included IRDye 800CW goat anti-mouse IgG (H+L) (LI-COR, cat. no. 926-32210) and HRP-conjugated goat anti-rabbit IgG (H+L) (Thermo Fisher Scientific, cat. no. 31462). Therapeutic antibody-based agents used in in vivo studies included trastuzumab deruxtecan (T-DXd/DS-8201a), a HER2-targeted antibody–drug conjugate, and amivantamab, an EGFR/MET bispecific antibody
Validation	All antibodies used in this study were obtained from commercial sources and used according to the manufacturers' recommendations. Antibodies selected for immunohistochemistry, flow cytometry and immunoblotting have been previously validated by the manufacturers and/or reported in peer-reviewed publications. Specificity was further supported through the use of appropriate positive and negative controls, isotype controls where indicated, expected molecular weights in immunoblotting experiments, and concordant pharmacodynamic changes following genetic or pharmacologic perturbation of the RAS/MAPK pathway. For immunohistochemistry studies, staining patterns were consistent with the expected cellular localization of the target proteins. For phospho-protein analyses, signal modulation following treatment with RAS(ON) inhibitors was consistent with pathway inhibition and biological expectations. No antibody generated unexpected staining patterns or immunoblot signals that would suggest lack of specificity.

Eukaryotic cell lines

Policy information about cell lines and Sex and Gender in Research

Cell line source(s)	Human pancreatic cancer cell lines included Capan-1 (ATCC HTB-79), HPAF-II (ATCC CRL-1997), HPAC (ATCC CRL-2119), MIA PaCa-2 (ATCC CRL-1420), and PSN-1 (ATCC CRL-3211), as well as KP-4 cells obtained from RIKEN (RCB1005). The HuP-T3 human pancreatic cancer cell line was genetically engineered to express doxycycline-inducible $KRAS^{g12R}$ for modeling KRAS overexpression. Murine pancreatic models included the AP009 $Kras^{g12D/wt}$;$Trp53^{null/null}$ organoid-derived cell line and derivatives engineered to overexpress $Kras^{g12D}$. Genetically engineered mouse model (GEMM)-derived pancreatic tumor cell lines KPCYc2, KPCYc3, KPCYc4, and KPCYc5 were obtained from Kerafast and used in vivo for efficacy studies. Patient-derived xenograft (PDX) models included WU-0002 ($KRAS^{g12D}$), WU-0022 ($KRAS^{g12R}$), and Pa01C ($KRAS^{g12D}$). In addition, several acquired resistance models were generated during the course of this work, including daraxonrasib-resistant Capan-1 xenografts established through serial in vivo passaging under continuous daraxonrasib treatment, RMC-7977-resistant HPAF-

	II xenografts generated through prolonged RAS(ON) inhibitor exposure, and an RMC-9945-resistant HPAC-derived cell line established from relapsed xenograft tumors and characterized by acquired $KRAS^{g12D}$ amplification. These models were used to investigate mechanisms of acquired resistance and evaluate rational combination therapy strategies in pancreatic cancer.
Authentication	All commercially obtained cell lines were authenticated by the originating repositories (ATCC, RIKEN, or other suppliers) before distribution. Cell line identity was routinely verified by short tandem repeat (STR) profiling performed by IDEXX BioAnalytics and compared with reference profiles when available. Engineered and acquired-resistance derivatives were generated from authenticated parental lines and validated by molecular profiling, including sequencing, digital PCR, and assessment of expected genomic alterations.
Mycoplasma contamination	Cell lines were routinely tested and confirmed to be free of mycoplasma contamination throughout the course of the study.
Commonly misidentified lines (See ICLAC register)	NA

Animals and other research organisms

Policy information about studies involving animals; ARRIVE guidelines recommended for reporting animal research, and Sex and Gender in Research

Laboratory animals	Female BALB/c nude mice (6–8 weeks old; Zhejiang Vital River Laboratory Animal Technology Co., Ltd. or Shanghai Sino-British SIPPR/BK Laboratory Animal Co., Ltd.) were used for Capan-1, daraxonrasib-resistant Capan-1, KP-4, and PSN-1 xenograft studies. Female athymic nude mice (6 weeks old) were used for HPAC xenograft studies. Female SCID Beige mice (6–8 weeks old; Beijing Vital River Laboratory Animal Technology Co., Ltd.) were used for HPAF-II xenograft studies. Female NSG mice (8–12 weeks old; The Jackson Laboratory) were used for MIA PaCa-2 CDX studies and WU-0002, WU-0022, and Pa01C PDX studies. Female C57BL/6J mice (6–8 weeks old; The Jackson Laboratory) were used for KPC/Y GEMM-derived syngeneic tumor studies.
Wild animals	NA
Reporting on sex	Female
Field-collected samples	NA
Ethics oversight	All animal experiments were conducted in compliance with institutional and national guidelines for the care and use of laboratory animals and were approved by the relevant IACUCs.

Note that full information on the approval of the study protocol must also be provided in the manuscript.

Clinical data

Policy information about clinical studies

All manuscripts should comply with the ICMJE guidelines for publication of clinical research and a completed CONSORT checklist must be included with all submissions.

Clinical trial registration	The Phase 1/2 study of daraxonrasib in patients with advanced RAS-mutant solid tumors was registered at ClinicalTrials.gov (NCT05379985) before patient enrollment.
Study protocol	Clinical trial registration. The Phase 1/2 study was registered at ClinicalTrials.gov (NCT05379985) prior to patient enrollment. The full study protocol and all protocol amendments are provided with the Supplementary Information accompanying this manuscript.
Data collection	Clinical, radiographic, safety and biomarker data were collected prospectively from patients enrolled in the Phase 1/2 study (NCT05379985) according to the study protocol. Tumor assessments were performed using RECIST v1.1, and blood samples for ctDNA analyses were collected at protocol-defined time points before, during and after treatment. Data were recorded using an electronic data capture system and analyzed by the sponsor in collaboration with study investigators.
Outcomes	Prespecified clinical outcomes included safety, tolerability, objective response rate, duration of response, progression-free survival, overall survival, and disease control rate, assessed according to RECIST v1.1. Exploratory translational outcomes included longitudinal ctDNA analyses and characterization of treatment-emergent genomic alterations associated with acquired resistance. The analyses presented in this manuscript focus on paired pre-treatment and end-of-treatment ctDNA samples collected from patients who achieved disease control on daraxonrasib and subsequently progressed on therapy.

Plants

Seed stocks	*Report on the source of all seed stocks or other plant material used. If applicable, state the seed stock centre and catalogue number. If plant specimens were collected from the field, describe the collection location, date and sampling procedures.*
Novel plant genotypes	*Describe the methods by which all novel plant genotypes were produced. This includes those generated by transgenic approaches, gene editing, chemical/radiation-based mutagenesis and hybridization. For transgenic lines, describe the transformation method, the number of independent lines analyzed and the generation upon which experiments were performed. For gene-edited lines, describe the editor used, the endogenous sequence targeted for editing, the targeting guide RNA sequence (if applicable) and how the editor was applied.*
Authentication	*Describe any authentication procedures for each seed stock used or novel genotype generated. Describe any experiments used to assess the effect of a mutation and, where applicable, how potential secondary effects (e.g. second site T-DNA insertions, mosiacism, off-target gene editing) were examined.*

Flow Cytometry

Plots

Confirm that:

☒ The axis labels state the marker and fluorochrome used (e.g. CD4-FITC).

☒ The axis scales are clearly visible. Include numbers along axes only for bottom left plot of group (a 'group' is an analysis of identical markers).

☒ All plots are contour plots with outliers or pseudocolor plots.

☒ A numerical value for number of cells or percentage (with statistics) is provided.

Methodology

Sample preparation	Cells were harvested from culture, washed with phosphate-buffered saline (PBS), and stained with Alexa Fluor 647-conjugated anti-human HER2 antibody (BioLegend, cat. no. 324412) at 4 μg ml^{-1} according to the manufacturer's recommendations. Following staining, cells were washed and resuspended in PBS for flow cytometric analysis.
Instrument	Flow cytometry data were acquired using a FACScan 5 flow cytometer (BD Biosciences).
Software	Data were analyzed using FlowJo software (BD Biosciences).
Cell population abundance	Flow cytometry was performed to quantify surface HER2 expression in cultured pancreatic cancer cell lines. No rare cell populations were analyzed or sorted.
Gating strategy	Cells were identified based on forward- and side-scatter characteristics to exclude debris. Single cells were gated using forward-scatter parameters, and HER2-positive populations were quantified based on Alexa Fluor 647 fluorescence intensity relative to unstained and/or control populations. Representative gating strategies are provided in the Source Data or Supplementary Information, where applicable.

☐ Tick this box to confirm that a figure exemplifying the gating strategy is provided in the Supplementary Information.
