# Supplementary material for: Acquired resistance to the RAS(ON) multi-selective inhibitor daraxonrasib guides rational combination therapy strategies in pancreatic cancer
Source: Nat Med. 2026 Aug 11;32(8):2865–77. doi: 10.1038/s41591-026-04537-w (PMC13472880; doi:10.1038/s41591-026-04537-w)
Supplement: Supplementary file 2 [file 41591_2026_4537_MOESM2_ESM.pdf]

nature portfolio

Peer Review File

Acquired resistance to the RAS(ON) multi-selective inhibitor daraxonrasib (RMC-6236) guides rational combination therapy strategies in pancreatic cancer

Corresponding Author: Dr Mallika Singh

This file contains all reviewer reports in order by version, followed by all author rebuttals in order by version.

Version 1:

Reviewer comments:

Reviewer #1

(Remarks to the Author)
Aronchik, Kar et al report manuscript ‘ Acquired resistance to the RAS(ON) multi-selective inhibitor daraxonrasib (RMC-6236) guides rational combination therapy strategies in pancreatic cancer’

44 patients from the phase 1/2 trial are analysed using Guardant Health Infinity NGS panel and 59% have evidence of genetic causes of acquired resistance including KRAS amplification in 1/3, 25% had multiple mechanisms of resistance (Not all had KRAS detected in pre treatment ctDNA). Broadly, acquired resistance is categorised into amplification in RAS, MYC, RTKs with acquisition of mutations in PI3K genes Findings are supported by in vitro/vivo data. Extensive pre-clinical resistance models are documented with resistance mechanisms similar to ctDNA findings which is important giving limitations of ctDNA

Combination experiments in pre-clinical models are provided suggesting roles for DDR agents, RTKi or combination or with allele specific inhibitors

This is an excellent paper which provides the community with important resistance data for future strategies. I do have comments below

Comments

1. N=134 on the second line trial at doses 160-300mg (paper included), 168 in total in phase 1/2, but study includes 44, can authors confirm where all patients include from the second line study or how were the 44 patients ‘selected’ understanding that disease control was a selection criteria as was the 160mg-300mg dosing – could a consort be provided (applies point 5)
2. Acquired resistance is defined as >3 months DCR, where patients scanned every 6 weeks on study? Would be helpful here to see VAF changes in KRAS in Fig1a, did all have initial decline in KRAS VAF? Eg pt 28 we see a KRAS VAF of 4.22 pre to 12.69 at end (supp table), if that patient progressed at second scan and the first showed growth but within SD criteria, this likely reflects de novo resistance if no KRAS VAF response.
3. In the 18 patients with no putative acquire genetic resistance mechanisms was tumour volume low and what was the KRAS VAF of these patients. In extended table 4 there are a number of patients where pre-treatment KRAS not detected in ctDNA, this would be important to summarise sensitivity and specificity of ctDNA analysis
4. It would seem a little confusing to add 13 additional patients at lower doses, without introducing earlier in manuscript, again denominator here important with consort to better understand. - (point 2 on benefit of consort even if supplemental)
5. Why were cases of de novo resistance not included given literature suggesting KRAS amp at the outset, would this not be also important for the pre-clinical combo data (also point 1, if SD but growth and rise in KRAS VAF are these not de novo resistant)
6. Can authors confirm there were no CNG or amplification in CDK 4/6
7. Authors state no secondary KRASm unlike G12C off inhibitors, however even in G12C inhibitors, the secondary KRAS m are most common in NSCLC and CRC even in G12C inhibitors, so this could be a PDAC phenomenon rather than inhibitor, this is in discussion but could be better introduced

Modelling acquired resistance

1. This paragraph is confusing, goes from no acquired secondary mutations in KRas which the authors confirm in vitro, to mentioning BRAF mutations (not describing pre-clinical modelling rather stating and why only BRAF here ?) back to no second site mutations in PDAC, perhaps this paragraph should entitle – Absence of acquired secondary site mutations in KRAS

Ras pathway hyperactivation
1. These experiments are very important to support findings, do authors believe these would also support mechanisms of de novo resistance and as such could data from patients be included if available

Combinations (Cell cycle, MYC, RTK, allele specific combinations)

1. For experiments with the WEE-1 inhibitor, CHK1 inhibitor and ATR inhibitor are all models represented by KRAS/TP53 and do the combinations have activity in absence of TP53, or in presence GOF mutations

Discussion

1. Doesn't discuss/summarise the DDR therapeutic opportunities as for other combinations, this should be briefly expanded upon.

Reviewer #2

(Remarks to the Author)
This is a timely work on resistance to daraxsonrasib. cfDNA implicates KRAS CN gain in many cases, a MET fusion and MET Cbl domain hotspot acquired, many RAF activation events in one patient and a ERBB2 amp in one patient.
In vivo models are used to show that KRAS amp occurs in some cases with derived resistant cell lines. Some work is also done to show some cell lines with pre-existing MET for example (KP4 cells) do respond to dual treatment with avimantinab targeting EGFR and MET.
P53 mutants may both provide a mechanism of KRAS amp as well as a liability for DDR stress. ATRi and Wee1 are synergistic in vitro and additive / synergistic in vivo
Finally, evidence is presented that G12D inhibition + "multiRas" inhibition may hold promise for not totally clear reasons.
The report is certainly timely and highly relevant. The authors are to be congratulated for their translational efforts on early – treated patients.
Many of the cfDNA observations are not corroborated by orthogonal (e.g. proteomic) methods, which calls into question how uniform these findings are even in a single patient. Given the difficulty in obtaining "end of treatment" biopsies, this is of course understandable.
The cell line work seems to support the emergence of increase RAS-RAF-ERK flux in the setting of acquisition of resistance and Dox-On KRAS experiments seem to confirm in HupT4 that increased KRAS signalling does impart some degree of resistance.
Some specific comments:
Page 5 of manuscript and Figure 2.
• Unclear whether figure 2 a-f and extended data 1 are all showing the same type of data per patient.
• For Fig 2 a&b as well as Ext Figure 1, the data is represented in tables. And Fig2 c-f are charts with varying x-axes (maybe due to dissimilar data collection points?)
• Extended figure 1 has VAF represented as a number and not a % (inconsistent with other figure)
Page 6 of manuscript and extended data 3
• Relevance of DMS to wider manuscript? Seems thrown in as these mutations were not found in patients and not found later in vivo/in vitro (as stated in top of page 7)
o Any clinical evidence of these mutations in wider populations?
Page 7
• A section of a figure illustrating what was done to cells in what order for the in vivo experiments would be helpful. I'm finding it difficult to keep clear what was done to which cells before/after implantation.
o This is explained some in the methods, but it doesn't reference the experiments within their context
Page 9
• "the combination [of azenosertib and daraxonrasib] drove deep and relatively durable tumor regressions at tolerated doses of both inhibitors, although these were not substantively different from those seen with single agent daraxonrasib at the optimal dose" suggests that there is not synergy between these drugs but is followed by "these results are consistent with the DDR machinery representing a therapeutic vulnerability in RAS mutant PDAC in the context of TP53 loss of function" which implies to me that it is a viable combinatorial therapy
o Is there a reason to pursue this? Is azenosertib better tolerated?
o The next section is fine stating that the combinatorial potential is limited
Page 11-12 Figure 5
• This figure is using at least 4 different cell lines. It's difficult to keep straight which models were used and why.
o Dosing varies by cell line
o Do the c3, c4, and c5 experiment (5c and 5d), the mice receive the full dose of both single agents. Is the effect between the two synergistic, additive, or subadditive? Should calculate this somewhere

the conceptual reasons for using two RAS inhibitors is not clear. is this stochimetric, structural (two drugs per molecule)? pharmacokinetic or kinetic (e.g. Km)

Reviewer #4

(Remarks to the Author)
In this original article, Aronchik et al. report results from an ancillary study analyzing paired pre- and end-of-treatment ctDNA samples from 44 patients who developed acquired resistance after initial partial response (PR) or stable disease (SD). The authors identify KRAS amplification as the predominant mechanism of resistance in 59% of cases, alongside other alterations involving RTK, MAPK, and PI3K pathways, and notably the absence of secondary KRAS mutations. Building on these findings, they perform a comprehensive series of preclinical experiments, using diverse in vitro and in vivo models of acquired resistance, to evaluate targeted combination strategies aiming to overcome this resistance.
Overall, this is a timely and important study for the translational and clinical oncology community. However, the experimental design is complex, combining multiple preclinical models, inhibitors, and co-occurring molecular alterations, and would benefit from additional clarification for the reader.

1. Patient population
• The authors indicate that their work derives from the phase I/II trial NCT05379985, which enrolled 38 evaluable patients at data cutoff. However, Extended Table 4 presents data from 57 patients (with PR/SD, at doses below and above 160 mg), and analyses focus on 44 patients treated at doses >160 mg. Clarification is needed on whether all eligible patients with PR/SD and doses >160 mg were included, and if not, whether these are representative of the overall population. What about patients with progressive disease (PD)? A flowchart summarizing patient selection and analysis subsets would help clarify this.
• It would also be valuable to analyze patients with upfront progression (PFS < 3 months) to describe their baseline genomic profiles and early acquired alterations, since amplification or emergent mutations might appear rapidly after RAS inhibitor exposure. This analysis could help contextualize combination therapy strategies according to primary/early vs. late resistance mechanisms.

2. KRAS amplification
KRAS amplification is well established by ISH on tissue, but its detection on ctDNA can be more challenging. The definition criteria for KRAS amplification in ctDNA and the denominator used are not clearly described. Additionally, the degree of copy number gain (CNV 2–3 versus higher levels) should be specified in the table, as this likely impacts biological significance. Some examples lack pre-existing amplification, yet preclinical models show baseline KRAS gains—please specify baseline CNV for all patients.
Concerning the statement “reduction in VAF demonstrates absence of KRAS amp,” this may not be entirely accurate. A drop in VAF could also reflect tumor response while amplification emerges in resistant subclones. Thus, clearer criteria for KRAS amplification calling in ctDNA are necessary.
Importantly, it remains uncertain whether KRAS amplification causes resistance or merely represents a reactive or compensatory mechanism to RAS inhibition. The Western blot data show limited evidence of pathway hyperactivation (pERK/ERK increase), with only one clear example (Fig. 3b). Even in resistant models harboring KRAS amplification, pERK decreases upon treatment, suggesting dose-dependent rather than absolute resistance. This is consistent with the observation that the HPAF-II model, despite KRAS amplification, retains sensitivity at baseline (Fig. 3j), potentially explaining the non-fully flattened tumor growth curves upon treatment (Fig. 3f, 3i).

3. Preclinical models of resistance
Interpretation is made difficult by the heterogeneous models and compounds used. The study includes pan-RAS inhibitors (daraxonrasib, RMC-7977) and KRAS G12D-selective inhibitors (zoldonrasib, RMC-9945). A comparative summary table outlining each compound’s mechanism of action would improve readability. Extended Table 5 is helpful but should be expanded to report, for each model (cell line, or in vivo model):
• Baseline molecular alterations
• Mechanism(s) of acquired resistance (exposure to drug X or Y…)
• All secondary alterations identified
For instance, the HPAF-II model exhibits baseline KRAS amplification, yet the secondary resistance mechanisms are not detailed. Similarly, the KBC/Y GEMM model is not characterized at baseline for genomic alterations or amplification status. Considering the heterogeneity in resistance mechanisms observed in patients, some degree of intra-model heterogeneity could be expected among CDX mice; have the authors replicated resistance patterns across experiments? Since resistance appears multifactorial, variability between animals is plausible. Moreover, to demonstrate the therapeutic relevance of drug combinations, sequential experiments (RAS inhibitor monotherapy followed by combination at regrowth) would be particularly informative. Clarification on whether coexisting alterations occur within the same clones or across subclones (e.g., via single-cell analyses) is also of interest.

4. Combination strategies
In tumors with TP53 loss or genomic instability, chemotherapy could mimic the effect of DDR inhibitors by inducing DNA damage. Thus, combinations with platinum compounds or consideration of HRD alterations (e.g. ATM acquired mutations) merit discussion.
The T-DXd combination is compelling, but since it is an ADC, distinguishing between the cytotoxic payload effect, targeted HER2 inhibition, or both would be important. Comparative experiments with trastuzumab or HER2 TKIs could clarify this.
Finally, KRAS amplification may simply reduce drug efficacy through target saturation. If so, higher doses of daraxonrasib may partially overcome this, which would be interesting to evaluate relative to dual inhibition strategies.

5. Additional comments
• There are inconsistencies in the Extended Table numbering: Tables 1 and 5 appear identical, while Ext Table 1 itself is missing. Please verify and correct the numbering throughout.
• Provide exact sample sizes per group in all panels.
• Figure 1: Include KRAS copy number data.
• Figure 3a and 3d: Clarify whether genuine pERK/ERK hyperactivation occurs.
• Extended Tables 1–3: ; clarify patient ordering and color coding in table ;
• Ext Table 2 : complete with baseline genomic data
• Ext Table 3 : clarify patient ordering and color coding; add the co alterations
• Ext Table 4 : same point about patient ordering, indicate whether blank rows (4 patients #29, 5, 38, 57) reflect missing data or no alteration detected.
• Comment on the relative sensitivity of ctDNA vs tissue testing (e.g. for KRAS mutation detection).
• Author contributions are very generic; please specify individual responsibilities.

Version 2:

Reviewer comments:

Reviewer #1

(Remarks to the Author)
The manuscript has benefited from review and revisions and I am happy with response and revisions to issues raised

Reviewer #2

(Remarks to the Author)
Aronchik and Kar et al resubmit their work on resistance to Daraxonrasib.
the authors have acceptably replied to most of reviewers' criticisms.
One exception is that the DMS data, while elegant, does not seem to serve any purpose in this manuscript. If space is limiting, that might go, but otherwise it is a lot of great work and I congratulate the authors for their vigorous and timely pursuit of this important problem in this poorly treated disease.

Reviewer #4

(Remarks to the Author)
The revisions appropriately address the concerns I previously raised.
The authors have responded to my comments in a thorough and satisfactory manner.

Open Access This Peer Review File is licensed under a Creative Commons Attribution 4.0 International License, which

permits use, sharing, adaptation, distribution and reproduction in any medium or format, as long as you give appropriate credit to the original author(s) and the source, provide a link to the Creative Commons license, and indicate if changes were made.
In cases where reviewers are anonymous, credit should be given to 'Anonymous Referee' and the source.
The images or other third party material in this Peer Review File are included in the article's Creative Commons license, unless indicated otherwise in a credit line to the material. If material is not included in the article's Creative Commons license and your intended use is not permitted by statutory regulation or exceeds the permitted use, you will need to obtain permission directly from the copyright holder.
To view a copy of this license, visit https://creativecommons.org/licenses/by/4.0/

Point by Point Responses to Referees

Referee #1 (Remarks to the Author):

Aronchik, Kar et al report manuscript ' Acquired resistance to the RAS(ON) multi-selective inhibitor daraxonrasib (RMC-6236) guides rational combination therapy strategies in pancreatic cancer'

44 patients from the phase 1/2 trial are analysed using Guardant Health Infinity NGS panel and 59% have evidence of genetic causes of acquired resistance including KRAS amplification in 1/3, 25% had multiple mechanisms of resistance (Not all had KRAS detected in pre treatment ctDNA). Broadly, acquired resistance is categorised into amplification in RAS, MYC, RTKs with acquisition of mutations in PI3K genes Findings are supported by in vitro/vivo data. Extensive pre-clinical resistance models are documented with resistance mechanisms similar to ctDNA findings which is important giving limitations of ctDNA

Combination experiments in pre-clinical models are provided suggesting roles for DDR agents, RTKi or combination or with allele specific inhibitors

This is an excellent paper which provides the community with important resistance data for future strategies. I do have comments below

We thank Referee #1 for the thoughtful and positive assessment of our manuscript and for recognizing the importance of these results for the field. We appreciate the referee's careful evaluation of both the clinical ctDNA analysis and the extensive preclinical modeling used to support and extend the resistance findings. We are grateful for the acknowledgment of the relevance of these resistance mechanisms and the proposed

combination strategies for future therapeutic development. We have addressed all comments raised below and believe that the resulting revisions further strengthen the clarity, rigor, and translational impact of this study.

R1-1

N=134 on the second line trial at doses 160-300mg (paper included), 168 in total in phase 1/2, but study includes 44, can authors confirm where all patients include from the second line study or how were the 44 patients 'selected' understanding that disease control was a selection criteria as was the 160mg-300mg dosing – could a consort be provided (applies point 5)?

We appreciate the referee (along with referee #4) highlighting the need to clarify patient denominators and selection criteria for the 44 patients profiled herein. We have now added a CONSORT flow diagram to the supplement (below and Extended Data Figure 1a, aligned with the corresponding clinical manuscript) which details patient enrollment, dose ranges, and the selection criteria for the 44 patients profiled by ctDNA with acquired resistance. All patients were from the phase 1/2 trial (NCT05379985) which included a total of 168 patients with PDAC who received daraxonrasib as second or later line of therapy. We have also clarified in the Results section that all patients included in this analysis were treated in the second-line or later setting.

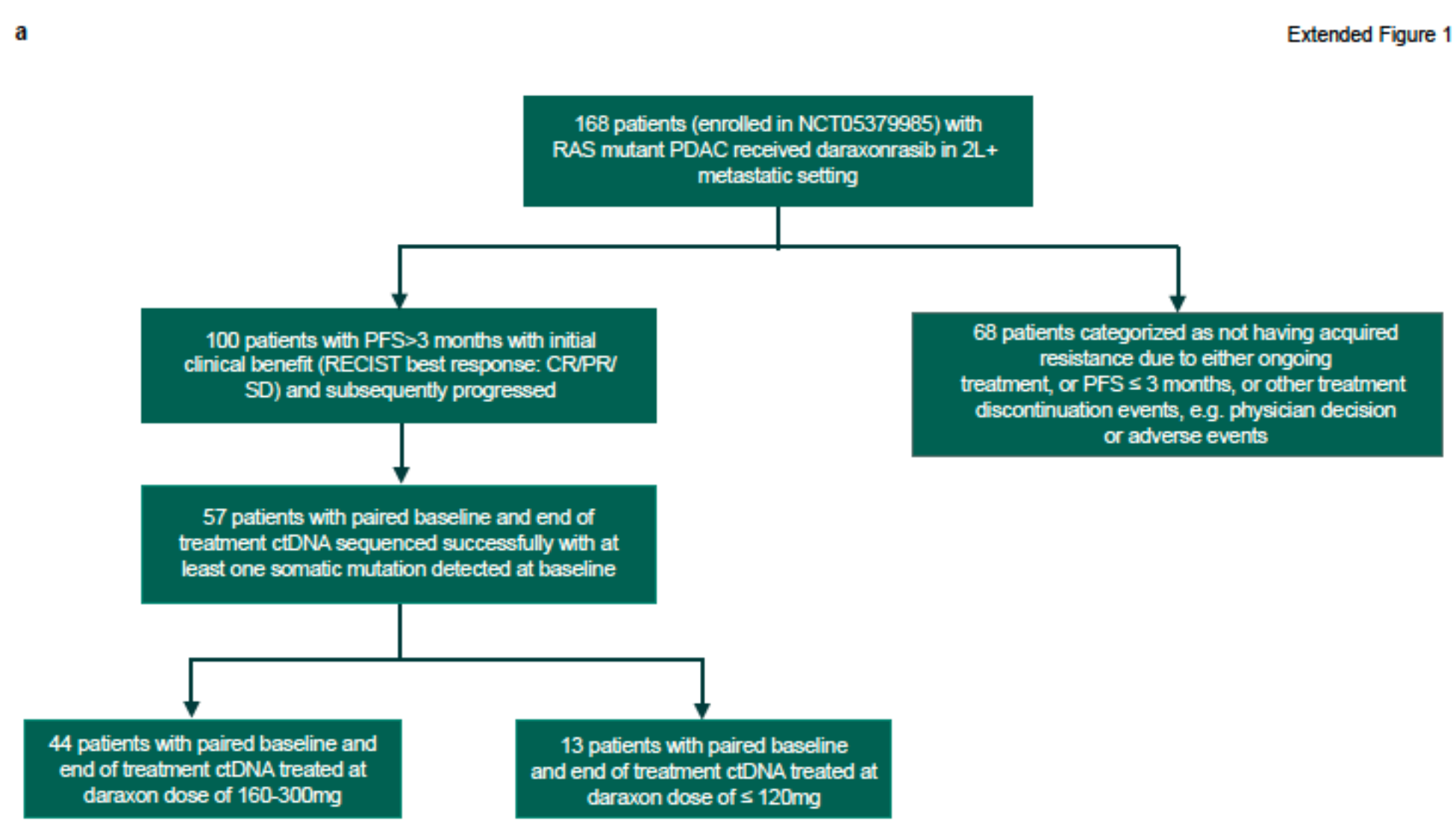

R1-2

Acquired resistance is defined as >3 months DCR, where patients scanned every 6 weeks on study? Would be helpful here to see VAF changes in KRAS in Fig1a, did all have initial decline in KRAS VAF?

Eg pt 28 we see a KRAS VAF of 4.22 pre to 12.69 at end (supp table), if that patient progressed at second scan and the first showed growth but within SD criteria, this likely reflects de novo resistance if no KRAS VAF response.

We thank the referee for a thoughtful request for more information on imaging intervals and ctDNA dynamics in the context of acquired resistance. As the referee notes, we defined clinical acquired resistance as following progression-free survival >3 months, consistent with similar studies defining acquired resistance after initial clinical benefit for which we have added citations in the Methods section (https://doi:10.1056/NEJMoa2303810; https://doi: 10.1056/NEJMoa2105281). Thus the 44 patients had SD, PR, or CR up to C5D1 (2nd scan with 21-day cycles). Patients with progression at the second scan were not included, while patients with stable disease were considered to have derived clinical benefit.

Per the referee's request, we have now expanded the supplementary materials in both tabular and visual form (Extended Data Table 1, Extended Data Figure 1b, and shown below) to include on-treatment RAS VAF at C2D1 for all 44 patients, demonstrating the early impact of daraxonrasib on this measure. Consistent with the clinical benefit observed, all patients demonstrated reductions or complete clearance of RAS VAF on treatment (if detectable at baseline), prior to progression.

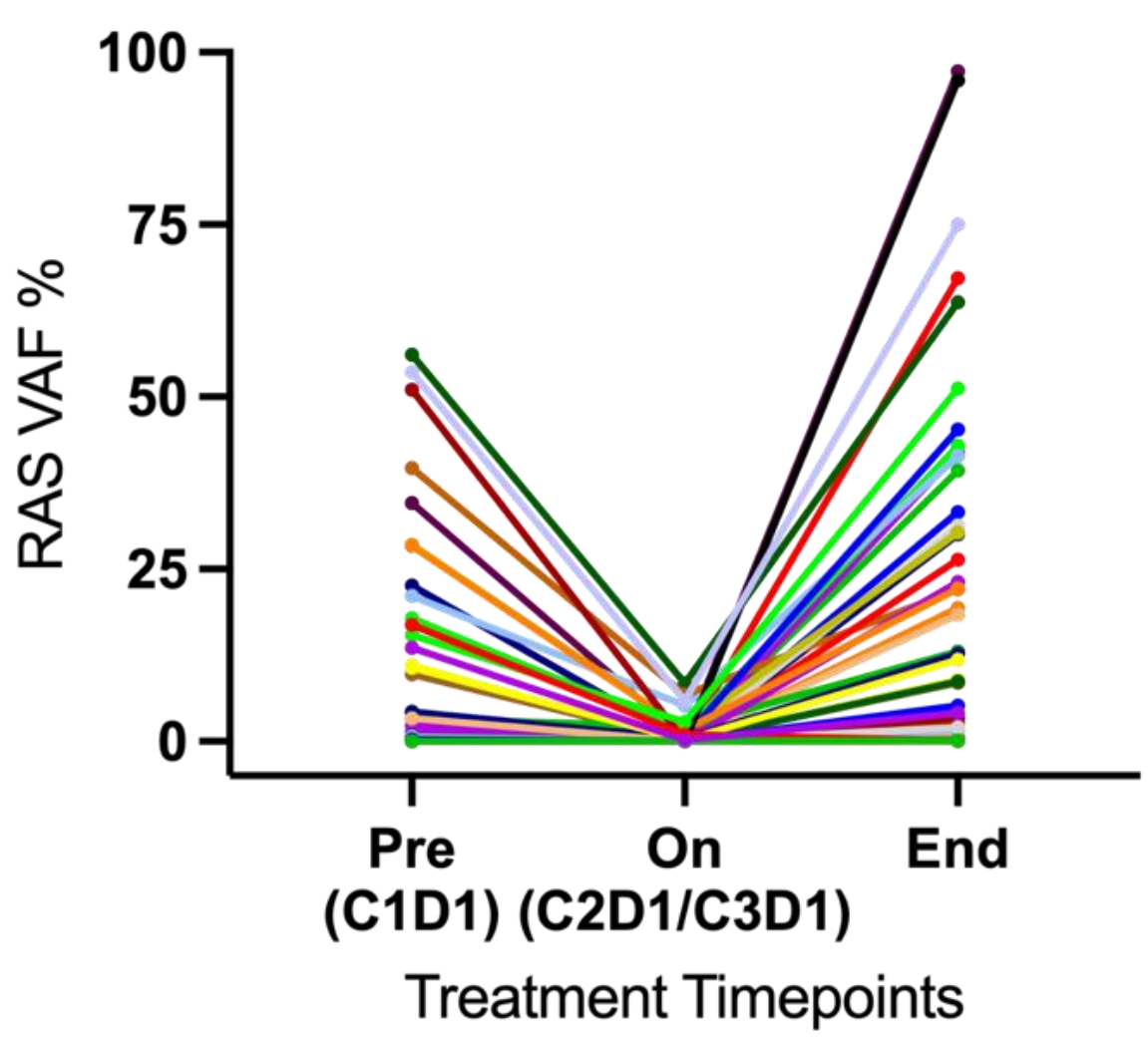

R1-3

In the 18 patients with no putative acquire genetic resistance mechanisms was tumour volume low and what was the KRAS VAF of these patients. In extended table 4 there are a number of patients where pre-treatment KRAS not detected in ctDNA, this would be important to summarise sensitivity and specificity of ctDNA analysis.

We appreciate the referee's request for additional details and have now added baseline KRAS VAF in the subgroup of 18 patients without identifiable acquired resistance mechanisms (Extended Data Table 1). As shown in the CONSORT diagram (above and Extended Data Figure 1a), all patients were required to have at least one somatic variant detected in pre-treatment ctDNA to minimize cases of low tumor shedding causing false positives for acquired alterations at progression, at least for mutation detection.

We detected acquired alterations in 13 of the 18 patients we characterize as having no putative genomic resistance mechanisms, shown in Extended Data Table 1; thus, assay sensitivity is likely less of a concern in these patients. However, as we previously describe in the Results section, we do not have evidence of these alterations being candidate resistance mechanisms. Rather, we believe these acquired alterations are likely to be CHIP alterations, or changes in tumor suppressor genes that were likely present in pre-treatment tumors (due to being early clonal events in PDAC) but not detected in pre-treatment ctDNA.

R1-4

It would seem a little confusing to add 13 additional patients at lower doses, without introducing earlier in manuscript, again denominator here important with consort to better understand. - (point 2 on benefit of consort even if supplemental).

Our thanks for highlighting the need to clarify the source and trajectory of 13 patients from this study treated at lower doses. These patients were part of the same 168 patient cohort in the Phase 1/2 clinical trial that received daraxonrasib treatment for PDAC in the second line or later setting– which included dose escalation at doses lower than 160 mg. We have now better introduced this cohort in the Results section and clarified within the newly added supplemental CONSORT diagram (also shown above). The text describes the source of these patients treated at lower doses, with similar clinical features and selection criteria for acquired resistance analysis as the cohort treated at >160 mg QD daraxonrasib.

R1-5

Why were cases of de novo resistance not included given literature suggesting KRAS amp at the outset, would this not be also important for the pre-clinical combo data (also point 1, if SD but growth and rise in KRAS VAF are these not de novo resistant)

The referee asks a very relevant question on mutant KRAS amplification at baseline. The frequency of this alteration is relatively uncommon in PDAC (≤5% in real world analyses, doi.org/10.1038/s41591-

024-03362-3), which limits the statistical power of *de novo* or baseline resistance mechanism analyses for KRAS amplification in the relatively small 44-patient data set examined herein. We also share the referee's interest in *de novo* resistance mechanisms; however, a rigorous evaluation of such *de novo* vs baseline resistance mechanisms would require a significantly larger patient cohort at a single dose with comprehensive baseline genomic profiling and extended clinical follow-up to enable adequately powered subgroup analyses. These efforts are ongoing with both the currently described Phase 1/2 study and the larger Phase 3 study (RASolute 302) mentioned in this manuscript. We plan to report these results separately when such analyses are completed, which we now acknowledge in the Discussion section.

In the current manuscript, we focused on acquired resistance mechanisms as an initial and timely body of work that can be achieved with a relatively small patient data set using paired baseline and EOT patient samples. While we cannot (yet) interrogate the question of whether therapeutic pressure via daraxonrasib treatment selects for pre-existing alterations and/or promotes *de novo* mechanisms, our finding that acquired genomic RAS pathway alterations (including mutant KRAS amplification) are more common at higher doses of daraxonrasib supports that these are selected for and likely acquired in the context of therapeutic pressure.

R1-6

Can authors confirm there were no CNG or amplification in CDK 4/6

We confirm that no CDK4/6 CN gains/amplifications were detected in ctDNA in these patients at baseline or at end of treatment.

R1-7

Authors state no secondary KRASm unlike G12C off inhibitors, however even in G12C inhibitors, the secondary KRAS m are most common in NSCLC and CRC even in G12C inhibitors, so this could be a PDAC phenomenon rather than inhibitor, this is in discussion but could be better introduced

We thank the referee for this insightful comment. We agree that the absence of acquired secondary KRAS mutations in our study may reflect, at least in part, a PDAC-specific phenomenon. We now elaborate on this context earlier in the revised Discussion and balance this with our findings also being consistent with the distinct mechanism of action of daraxonrasib as a RAS(ON) multi-selective tri-

complex inhibitor. Unlike KRAS G12C OFF-state inhibitors, daraxonrasib directly targets the active, GTP-bound form of RAS and (as previously described) retains inhibitory activity against several classes of secondary RAS mutations associated with acquired resistance to inactive-state KRAS G12C inhibitors (doi.org/10.1038/s41586-024-07205-6; doi.org/10.1158/2159-8290.CD-24-0027; doi.org/10.1158/2159-8290.CD-24-0421). These mechanistic distinctions may contribute to the lack of secondary KRAS mutations observed in the PDAC cohort described herein. Interestingly second site mutations at KRAS Y64 were observed at end of treatment with daraxonrasib in a mixed non-PDAC cohort in the same clinical trial (doi.org/10.1101/2025.04.24.649345). Substitutions at this position lower the affinity of the daraxonrasib:CYPA binary complex for active KRAS and therefore drive daraxonrasib resistance via a different mechanism than, for example secondary KRAS G12 mutations that drive resistance to inactive-state G12C inhibitors (doi.org/10.1016/j.ccell.2026.01.001). We have now expanded the manuscript text to clearly introduce these concepts and better integrate tumor-type biology with inhibitor-specific mechanistic considerations.

R1-8

Modelling acquired resistance

This paragraph is confusing, goes from no acquired secondary mutations in KRas which the authors confirm in vitro, to mentioning BRAF mutations (not describing pre-clinical modelling rather stating and why only BRAF here?) back to no second site mutations in PDAC, perhaps this paragraph should entitle – Absence of acquired secondary site mutations in KRAS

We appreciate the referee's helpful clarification regarding the structure and focus of this paragraph and agree that the prior presentation was confusing. We have revised the relevant section for clarity, retitled it as suggested to "Absence of acquired second-site mutations in KRAS," and reorganized the text to ensure a more coherent narrative flow. We now clearly explain the rationale for including BRAF alterations in this context and distinguish their relevance from the central message regarding the lack of second-site KRAS mutations observed in our study. We hope these revisions improve focus and align with the referee's recommendation.

R1-9

Ras pathway hyperactivation

These experiments are very important to support findings, do authors believe these would also support mechanisms of de novo resistance and as such could data from patients be included if available

We thank the referee for underscoring the importance of preclinical resistance models to examine the causative role(s) of clinically observed acquired genomic alterations. While we agree that the preclinical data could also support mechanisms of *de novo* resistance, as discussed above (comment 1-5) the relatively small patient cohort prohibits us from drawing definitive conclusions regarding baseline/*de novo* resistance mechanisms in the clinical setting. As noted above, a rigorous evaluation of *de novo* or baseline resistance mechanisms would require a substantially larger patient cohort, comprehensive baseline genomic characterization, and extended clinical follow-up to allow adequately powered subgroup and temporal analyses. We have added a note in the revised Discussion that dedicated studies to explore baseline vs *de novo* mechanisms of resistance are warranted.

R1-10

Combinations (Cell cycle, MYC, RTK, allele specific combinations)

For experiments with the WEE-1 inhibitor, CHK1 inhibitor and ATR inhibitor are all models represented by KRAS/TP53 and do the combinations have activity in absence of TP53, or in presence GOF mutations

We appreciate the referee's insightful question regarding the role of *TP53* status in the observed activity of WEE1, CHK1, and ATR inhibitor combinations. We agree that understanding the *TP53* context is critical for interpreting these findings and have now comprehensively reported the *TP53* status for all models included in these experiments along with additional genomic features (including those with KRAS/TP53 co-alteration) in Extended Data Table S5. In our experiments, the combinations with azenosertib and BBI-355 demonstrated combinatorial anti-tumor activity in the context of both wild type TP53 (HPAC model) and mutant TP53 (HPAF-II model). While loss of TP53 function is well established to increase reliance on the G2/M checkpoint and thereby sensitize tumors to WEE1, CHK1, and ATR inhibition, multiple studies indicate that replication stress driven by oncogenic signaling, particularly KRAS activation and oncogene amplification, can also confer DDR dependency independently of TP53 status (doi.org/10.1038/s41388-022-02291-0; doi.org/ 10.1038/s41467-023-39991-6). Oncogene amplification can specifically manifest under therapeutic pressure and this can further perturb DNA replication dynamics (doi.org/10.1038/s41573-022-00558-5; doi.org/10.1016/j.trecan.2021.06.003). Thus, although TP53 loss or mutation may potentiate sensitivity to DDR inhibition, our data support a model in which DDR inhibitors retain activity across TP53 contexts when tumors experience high intrinsic replication stress, as is characteristic of KRAS-driven PDAC or as a result of oncogene amplification in response to therapeutic pressure. This interpretation is consistent with emerging clinical and preclinical evidence suggesting that TP53 status may modulate,

but does not strictly determine, responsiveness to DDR-targeting strategies (doi.org/10.1053/j.jocp.2016.03.006; doi.org/10.14791/btrt.2025.0017).

R1-11

Discussion

Doesn't discuss/summarise the DDR therapeutic opportunities as for other combinations, this should be briefly expanded upon.

We thank the referee for this helpful suggestion and agree that the Discussion would benefit from a clearer summary of the therapeutic opportunities associated with targeting DNA damage response (DDR) pathways in combination with daraxonrasib, similar to the contextual framing provided for other combination approaches. In response, we have now expanded this section of the manuscript.

Specifically, we now outline how inhibition of RAS signaling can potentiate replication stress and impair DNA repair capacity, thereby creating biologically plausible opportunities for synergy with DDR-targeting agents such as WEE1, CHK1, and ATR inhibitors, citing rationale shared in response to the previous comment. As reflected in our preclinical studies, our findings support the concept that RAS-driven tumors may exhibit heightened vulnerability when both KRAS-driven proliferative signaling and DNA damage repair pathways are concurrently suppressed.

The revised Discussion now incorporates these considerations and articulates how our findings may motivate future exploration of DDR-based therapeutic strategies with daraxonrasib.

Referee #2 (Remarks to the Author):

This is a timely work on resistance to daraxsonrasib. cfDNA implicates KRAS CN gain in many cases, a MET fusion and MET Cbl domain hotspot acquired, many RAF activation events in one patient and a ERBB2 amp in one patient. In vivo models are used to show that KRAS amp occurs in some cases with derived resistant cell lines. Some work is also done to show some cell lines with pre-existing MET for example (KP4 cells) do respond to dual treatment with avimantinab targeting EGFR and MET. P53 mutants may both provide a mechanism of KRAS amp as well as a liability for DDR stress. ATRi and Wee1 are synergistic in vitro and additive / synergistic in vivo. Finally, evidence is presented that G12D inhibition + "multiRas" inhibition may hold promise for not totally clear reasons. The report is certainly timely and highly relevant. The authors are to be congratulated for their translational efforts on early –

treated patients. Many of the cfDNA observations are not corroborated by orthogonal (e.g. proteomic) methods, which calls into question how uniform these findings are even in a single patient. Given the difficulty in obtaining "end of treatment" biopsies, this is of course understandable. The cell line work seems to support the emergence of increase RAS-RAF-ERK flux in the setting of acquisition of resistance and Dox-On KRAS experiments seem to confirm in HupT4 that increased KRAS signalling does impart some degree of resistance.

We thank referee #2 for the careful, thoughtful, and comprehensive evaluation of our manuscript and for recognizing the timeliness and translational relevance of this work. We greatly appreciate the referee's detailed assessment of both the clinical cfDNA findings and the breadth of the preclinical modeling, as well as the acknowledgment of the challenges inherent to studying early resistance in patients treated on first-in-human trials. We also value the referee's constructive comments regarding the limitations of cfDNA-based analyses in the absence of routine orthogonal tissue or proteomic validation, which we now more explicitly acknowledge and discuss in the revised manuscript. In response, we have clarified the rationale, scope, and interpretive boundaries of the cfDNA observations and strengthened the Discussion to contextualize these findings alongside the functional in vitro and in vivo data that support increased RAS–RAF–ERK flux as a convergent resistance mechanism. We believe these revisions improve transparency and reinforce the biological conclusions, and we are grateful to the referee for their insightful feedback and encouragement of our translational efforts.

Some specific comments:

R2-1

General: Many cfDNA observations are not corroborated by orthogonal methods; understandable but should be acknowledged.

We thank the referee for identifying this limitation and completely agree that we should acknowledge this. We have updated the Discussion section to note that while we were able to verify acquired mutant KRAS amplification via paired tumor biopsies from Patient 2 (Results: Acquired Mutant $KRAS^{amp}$ in ctDNA from Patients with PDAC), we were unable to corroborate most cfDNA findings by application of orthogonal methods at this time. Future studies with paired tumor biopsy collections will be important to validate the initial ctDNA findings described herein.

R2-2

Page 5 of manuscript and Figure 2.

Unclear whether figure 2 a-f and extended data 1 are all showing the same type of data per patient.

Thank you for highlighting an inconsistency in Figure 2. Figure 2 highlights patients with acquired KRAS amplification, and further supplemental data for these patients (such as baseline genomic profile and RAS variant allele frequencies) are detailed in both Extended Data Table 1 and 3. The VAFs at different collection time points shown in the tables in Fig 2a and b are repeated in the line plots in Fig 2c-f. Fig 2a and b associate the genomic findings with tumor scans of clinical response and resistance. Meanwhile, we utilize line plots to visualize the changes in VAFs for mutant KRAS compared to other somatic mutations to demonstrate amplification of the mutant KRAS allele. To aid in matching the data types per patient, we have modified collection points and cycle naming in the line plots to match the rest of the figure and Extended Data Tables. We have also added language in the figure legend to clarify that the Extended Data Tables reference the same patients.

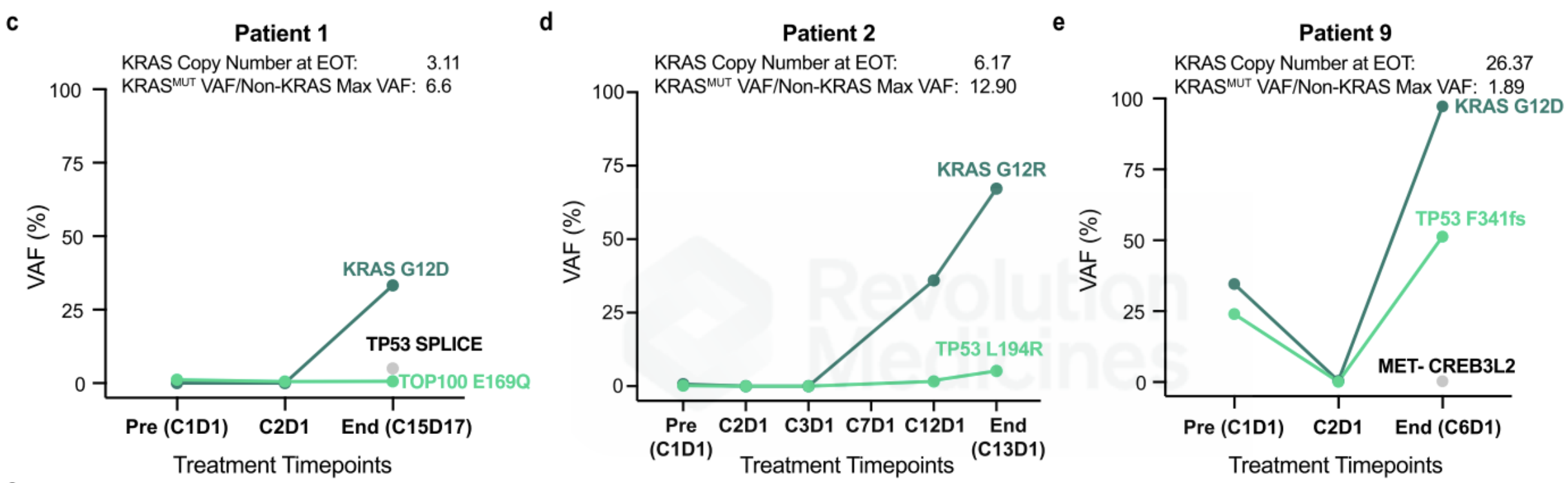

R2-3

Extended figure 1 has VAF represented as a number and not a % (inconsistent with other figure)

We thank the referee for highlighting the inconsistency in units for ctDNA. We have now corrected this in all relevant figures and tables, including Extended Data 1 (now Extended Data 2).

R2-4

Relevance of DMS to wider manuscript? Seems thrown in as these mutations were not found in patients and not found later in vivo/in vitro (as stated in top of page 7). Any clinical evidence of these mutations in wider populations?

We appreciate the referee's question regarding the relevance of the DMS analysis. Although the specific DMS-identified mutations were not observed in our current PDAC cohort, secondary RAS mutations predicted by the DMS were identified, albeit rarely, in a parallel study by Sang et al., in end-of-treatment ctDNA in other malignancies treated with daraxonrasib (https://doi.org/10.1101/2025.04.24.649345) as well as in orthogonal preclinical screens (https://doi.org/10.1101/2025.08.04.668444 and https://www.science.org/doi/10.1126/scisignal.adr3738). To date, we have not detected these events in the PDAC cohort described herein, potentially reflecting biological differences in PDAC, which (in the current study) appears more prone to acquiring amplifications of the original mutant KRAS allele rather than acquiring secondary RAS mutations. Or this may simply reflect the current cohort size, which may be insufficient to capture less frequent events.

Thus, the DMS screen serves as an important forward-looking tool to identify potential secondary RAS mutations that could compromise the mechanism of action of RAS(ON) inhibitors such as daraxonrasib. This information has been important for defining the breadth of on-target RAS mutations that have the potential to cause resistance to daraxonrasib, serving as a valuable reference for our ctDNA analyses. We have included the DMS data in the extended data section of the paper and believe that it will serve as a comprehensive dataset to cross-reference for any future emergent alterations with potential to disrupt RAS(ON) inhibitor binding or signaling.

R2-5

Page 7. A section of a figure illustrating what was done to cells in what order for the in vivo experiments would be helpful. I'm finding it difficult to keep clear what was done to which cells before/after implantation.

We agree that a visual representation of the experimental workflow would greatly improve clarity and assist readers in connecting specific in vivo manipulations to the corresponding results. In response, we have generated a schematic overview of resistance model development and the associated experimental workflow, summarizing the sequence of treatments and experiments underlying model development and characterization (below). This schematic has also been added to the revised manuscript (Supplementary Methods), and cross-referenced in both the Results and Methods sections to ensure clear navigation.

a. Schematic overview of resistant model development and experimental workflow

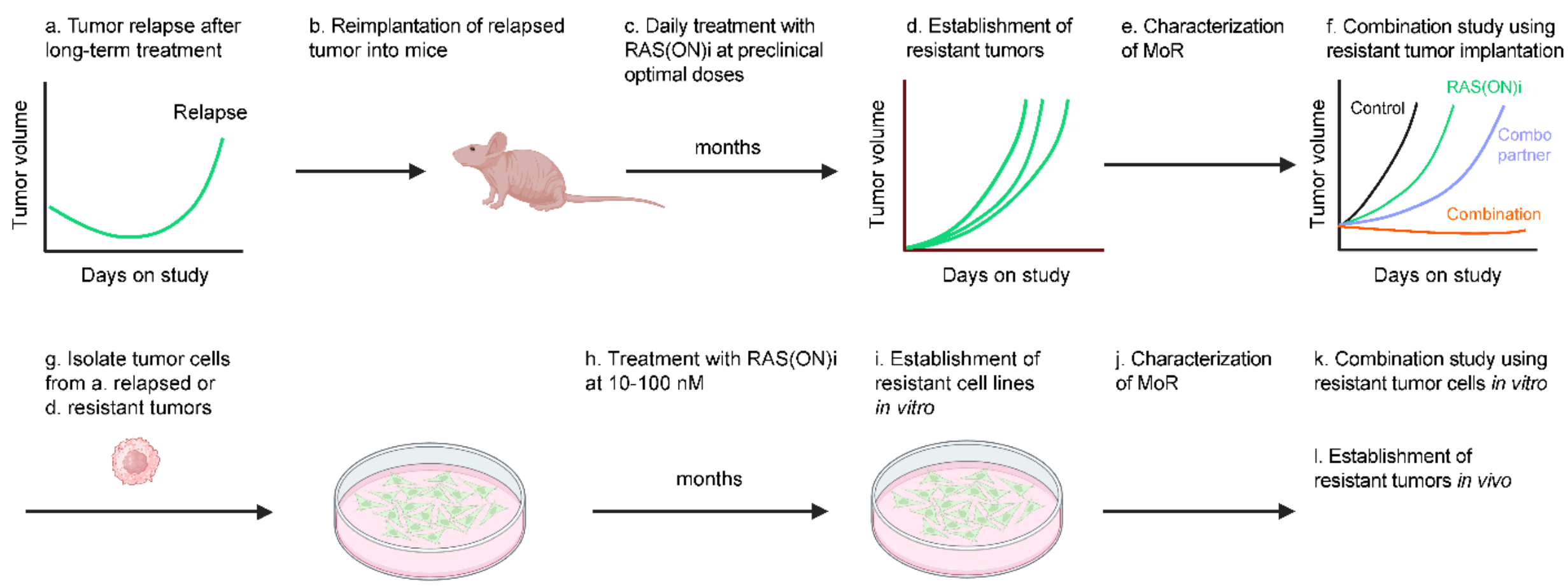

R2-6

Page 9 "the combination [of azenosertib and daraxonrasib] drove deep and relatively durable tumor regressions at tolerated doses of both inhibitors, although these were not substantively different from those seen with single agent daraxonrasib at the optimal dose" suggests that there is not synergy between these drugs but is followed by "these results are consistent with the DDR machinery representing a therapeutic vulnerability in RAS mutant PDAC in the context of TP53 loss of function" which implies to me that it is a viable combinatorial therapy. Is there a reason to pursue this? Is azenosertib better tolerated? The next section is fine stating that the combinatorial potential is limited

We agree that the benefit of the azenosertib + daraxonrasib combination is nuanced. While the magnitude of tumor regression observed is not consistently superior to optimal single-agent dosing of daraxonrasib, we believe that the combination is relevant given the distinct mechanisms underlying a similar antitumor response. In particular, targeting DNA damage repair pathways may help pre-empt or limit the emergence of additional oncogene amplifications, which are a well-described consequence of replication stress and genomic instability (doi.org/10.1016/j.cub.2014.04.012).

In this context, combining daraxonrasib with a DDR-targeting agent such as azenosertib may help reduce the likelihood of secondary genomic events—such as additional RAS-pathway gene amplifications emerging under therapeutic pressure. As the referee notes, the combination tolerability of RAS(ON) and DDR inhibitors is another important element that needs to be considered, which is best evaluated in the clinic. We have clarified this in the revised Discussion section.

R2-7

Page 11-12 Figure 5. This figure is using at least 4 different cell lines. It's difficult to keep straight which models were used and why. Dosing varies by cell line

We agree that the diversity of cell lines and dosing regimens made the figure difficult to follow and have now annotated the specific models used directly within the corresponding figure panels to aid clarity. In addition, we have added Extended Table 5 summarizing each model along with key features, and the dosing regimens employed. Overall, these revisions will facilitate interpretation of the data.

Specifically, in Figure 5, daraxonrasib (25 mg/kg) in combination with zoldonrasib (100 mg/kg) was well tolerated in syngeneic models using immunocompetent C57BL/6J mice. However, this same dosing regimen was not tolerated in xenograft models in immunodeficient mice. Therefore, a reduced dose of daraxonrasib (10 mg/kg) in combination with zoldonrasib (100 mg/kg) was used in the xenograft studies to ensure tolerability. This has now been elucidated in the Methods section.

R2-8

Do the c3, c4, and c5 experiment (5c and 5d), the mice receive the full dose of both single agents. Is the effect between the two synergistic, additive, or subadditive? Should calculate this somewhere, the conceptual reasons for using two RAS inhibitors is not clear. is this stochimetric, structural (two drugs per molecule)? pharmacokinetic or kinetic (e.g. Km)

We appreciate and share the referee's interest in formally assessing additivity and synergy in our in vivo combination studies. As included in the manuscript, we utilize statistically validated and established methods to assess combinatorial activity in vivo, including ANOVA-based analyses of tumor volume changes, depth and durability of treatment response, the latter via Kaplan-Meier analyses (doi.org/10.1038/nm.3954 ; doi: 10.1158/2159-8290.CD-24-0027). These methods have been commonly applied in in vivo efficacy studies to assess combinatorial benefits. Synergy-scoring frameworks such as Bliss or Loewe models, developed for controlled in vitro systems, have been extended to in vivo studies, however we have chosen not to adopt them given there is currently no consensus on their feasibility or applicability to in vivo combination data. We have clarified our approach in the revised text in Methods accordingly.

R2-9

Conceptual reasons for using two RAS inhibitors are not clear (stoichiometric/structural/pharmacokinetic/kinetic?).

We appreciate the referee's insightful request for greater clarity regarding the conceptual rationale for combining two RAS(ON) inhibitors. We articulate some of these below, and in the updated manuscript text.

- We have previously hypothesized and demonstrated that direct targeting of an oncogenic driver such as RAS is distinct from and results in improved responses as compared to upstream or downstream targeting (doi.org/10.1038/s41586-024-07205-6). This concept is now extended to the hypothesis that the combination of two direct RAS(ON) inhibitors could be a differentiated means to extinguish RAS pathway signaling in tumors and forestall resistance that results from pathway reactivation.

- The combination of a mutant-selective RAS(ON) inhibitor with multi-selective RAS(ON) inhibition maximizes suppression of the mutant KRAS oncogenic driver combined with multi-selective suppression of potential compensatory signaling via WT RAS, resulting in more complete suppression of RAS-GTP signaling flux than either agent alone (above) and forestalling resistance arising from RAS pathway reactivation. We have recently demonstrated this for the combination of elironrasib (a RAS(ON) G12C mutant-selective inhibitor) and daraxonrasib (doi: 10.1158/2159-8290.CD-25-1616). In the current manuscript, we demonstrate that these concepts extend to the combination of zoldonrasib and daraxonrasib, this is illustrated by our analysis of pERK in a series of allograft models as shown in figures 5e and f wherein each monotherapy drives clear but incomplete pathway suppression, which is demonstrably improved with the RAS(ON) inhibitor doublet. The combination benefit is anticipated to be most apparent in settings where RAS oncogenic output is high, e.g., due to gene amplification, or where adaptive resistance is mediated by increased RAS pathway flux.

We have added text to the Discussion to more clearly articulate these themes underlying the rationale for dual RAS inhibition, which form the scientific foundation for the pivotal RASolute 309 clinical trial currently being initiated by Revolution Medicines, testing the combination of zoldonrasib with daraxonrasib in patients with newly diagnosed metastatic PDAC.

Referee #4 (Remarks to the Author):

In this original article, Aronchik et al. report results from an ancillary study analyzing paired pre- and end-of-treatment ctDNA samples from 44 patients who developed acquired resistance after initial partial response (PR) or stable disease (SD). The authors identify KRAS amplification as the predominant mechanism of resistance in 59% of cases, alongside other alterations involving RTK, MAPK, and PI3K pathways, and notably the absence of secondary KRAS mutations. Building on these findings, they perform a comprehensive series of preclinical experiments, using diverse in vitro and in vivo models of

acquired resistance, to evaluate targeted combination strategies aiming to overcome this resistance. Overall, this is a timely and important study for the translational and clinical oncology community. However, the experimental design is complex, combining multiple preclinical models, inhibitors, and co-occurring molecular alterations, and would benefit from additional clarification for the reader.

We thank referee #4 for the thorough and positive evaluation of our study and for highlighting its translational relevance to the oncology community. We appreciate the recognition of the clinical and preclinical integration of our work, as well as the insightful comments regarding the complexity of the experimental design. In response, we have revised the manuscript to improve clarity and organization, with additional explanation and context provided to guide the reader through the study design, model selection, and combination strategy rationale. We believe these revisions address the referee's concerns and substantially enhance the accessibility and interpretability of the work.

1. Patient population

R4-1

The authors indicate that their work derives from the phase I/II trial NCT05379985, which enrolled 38 evaluable patients at data cutoff. However, Extended Table 4 presents data from 57 patients (with PR/SD, at doses below and above 160 mg), and analyses focus on 44 patients treated at doses >160 mg. Clarification is needed on whether all eligible patients with PR/SD and doses >160 mg were included, and if not, whether these are representative of the overall population. What about patients with progressive disease (PD)? A flowchart summarizing patient selection and analysis subsets would help clarify this

We thank the referee (along with referee 1) for highlighting the need to clarify selection of patients for this study from our Phase 1/2 study including 168 patients with PDAC who received daraxonrasib as second or later line of therapy (NCT05379985). We have now added a CONSORT flow diagram to clarify the clinical characteristics and selection of the 44 patients treated at >160 mg profiled in this study, which were representative of the characteristics of the 168 patients, e.g., line of therapy (Response R1-1 above and Extended Data Figure 1a).

All patients meeting RECIST 1.1 criteria for CR/PR/SD at doses >160 mg and PFS > 3 months (the definition of acquired resistance now described in Methods) and with successfully collected and sequenced paired ctDNA samples were included. Patients with a best response of PD (progression after first 21-day cycle) were not included as these did not meet the criterion of PFS > 3 months.

R4-2

It would also be valuable to analyze patients with upfront progression (PFS < 3 months) to describe their baseline genomic profiles and early acquired alterations, since amplification or emergent mutations might appear rapidly after RAS inhibitor exposure. This analysis could help contextualize combination therapy strategies according to primary/early vs. late resistance mechanisms.

We sincerely appreciate the ask from this referee and referee 1 to analyze baseline genomic profiles and early acquired alterations in this study and agree that characterization of *de novo* resistance (for example in patients with PFS < 3 months) will be invaluable for combination design strategy. However, as noted in responses 1-5 and 1-9, we find that a rigorous evaluation of *de novo* resistance vs baseline alterations requires a larger patient cohort and extended clinical follow-up to enable adequately powered subgroup analyses. These efforts are ongoing with data from the current Phase 1/2 study as well as a larger pivotal RASolute 302 study referenced in the manuscript, while the current submission focuses on analyses of acquired resistance which is feasible from the smaller patient cohort depicted herein. We have also added a note in the revised Discussion that dedicated studies of baseline, and *de novo* resistance are necessary in the near future.

2. KRAS amplification

R4-3

KRAS amplification is well established by ISH on tissue, but its detection on ctDNA can be more challenging. The definition criteria for KRAS amplification in ctDNA and the denominator used are not clearly described. Additionally, the degree of copy number gain (CNV 2–3 versus higher levels) should be specified in the table, as this likely impacts biological significance. Some examples lack pre-existing amplification, yet preclinical models show baseline KRAS gains—please specify baseline CNV for all patients.

We agree with the referee's recommendation and have incorporated these clarifications into the revised manuscript; we hope these sufficiently address the referee's comment. The Methods section now explicitly defines the ctDNA copy-number calling used in our analyses (see "Circulating Tumor DNA Assessment in Patients with Acquired Resistance"). We state that values greater than 2 are classified as copy-number gain; any patient without copy number amplification reported had two copies of the gene detected (diploid state). These criteria align with standard conventions for CNV interpretation in ctDNA, which treat CN = 2 as the diploid reference and call amplification when copy number exceeds this baseline (https://doi.org/10.1158/1078-0432.CCR-17-3831) Extended Data Table 3 lists the KRAS

copy number for patients with KRAS amplification acquired at EOT, and we have now added the KRAS copy number for the two patients with KRAS amplification detected at baseline in Extended data table 4.

R4-4

Concerning the statement "reduction in VAF demonstrates absence of KRAS amp," this may not be entirely accurate. A drop in VAF could also reflect tumor response while amplification emerges in resistant subclones. Thus, clearer criteria for KRAS amplification calling in ctDNA are necessary.

We appreciate the referee's clarification and agree that a reduction in VAF alone does not exclude the possibility that KRAS-amplified subclones may persist at low levels during treatment. It is also true that CNV calling sensitivity may decrease early on daraxonrasib treatment as overall ctDNA levels are reduced due to initial response. We have now modified the relevant sentence to avoid over interpretation and removed the previous claim. We have also now defined the amplification calling criteria as described in the previous response R4-3.

3. Preclinical models of resistance

R4-5

Interpretation is made difficult by the heterogeneous models and compounds used. The study includes pan-RAS inhibitors (daraxonrasib, RMC-7977) and KRAS G12D-selective inhibitors (zoldonrasib, RMC-9945). A comparative summary table outlining each compound's mechanism of action would improve readability. Extended Table 5 is helpful but should be expanded to report, for each model (cell line, or in vivo model):

- *Baseline molecular alterations*
- *Mechanism(s) of acquired resistance (exposure to drug X or Y…)*
- *All secondary alterations identified*

For instance, the HPAF-II model exhibits baseline KRAS amplification, yet the secondary resistance mechanisms are not detailed. Similarly, the KBC/Y GEMM model is not characterized at baseline for genomic alterations or amplification status.

We agree that our experimental approaches are complex and that providing a clearer comparative overview of the heterogeneous models and compounds strengthens the manuscript. We have now expanded Extended Table 5 to provide a comprehensive summary of the models and compounds used in the study. The revised table now includes, for each model: baseline molecular alterations, the resistance-induction method, identified secondary alterations, compounds tested and each compound's mechanism of action.

R4-6

Importantly, it remains uncertain whether KRAS amplification causes resistance or merely represents a reactive or compensatory mechanism to RAS inhibition. The Western blot data show limited evidence of pathway hyperactivation (pERK/ERK increase), with only one clear example (Fig. 3b). Even in resistant models harboring KRAS amplification, pERK decreases upon treatment, suggesting dose-dependent rather than absolute resistance. This is consistent with the observation that the HPAF-II model, despite KRAS amplification, retains sensitivity at baseline (Fig. 3j), potentially explaining the non-fully flattened tumor growth curves upon treatment (Fig. 3f, 3i).

We agree with the referee's astute observation that increased mutant KRAS expression resulting from KRAS amplification likely drives resistance via titration of the therapeutic effect of RAS inhibition by daraxonrasib. Our results are consistent with a shift in the inhibitory potency of daraxonrasib as a function of the expression level of mutant KRAS and the resultant impact on oncogenic flux through the RAS pathway. The present findings, together with those described in Sang et al. (doi10.1101/2025.04.24.649345); Wei et al. (doi:10.1158/2159-8290.CD-25-1616), Foth et al. (bioRxiv;doi:10.64898/2025.12.18.695203) and by others identify two major mechanisms by which RAS pathway reactivation can drive resistance to daraxonrasib monotherapy:

- Mechanisms such as mutant KRAS amplification and RTK fusions that result in increased oncogenic flux upstream, or at the level of RAS, will shift the concentration response curve to daraxonrasib or RMC-7977. In some 'resistant' models it may still be possible to achieve the tumor exposures necessary to suppress RAS pathway activity sufficiently to inhibit tumor growth, albeit the antitumor activity in these models may be reduced relative to the parental model, or an alternate model with reduced oncogenic flux. In other models, the extent of enhanced RAS pathway activation may be such that the higher exposures of the RAS(ON) multi-selective inhibitor required to achieve adequate pathway suppression are not tolerated *in vivo*, hence the apparent lack of antitumor activity. This would not reflect "absolute resistance"; for example, a combination of mutant selective and a multi-selective RAS(ON) inhibitors may still be able to achieve sufficient pathway suppression without tolerability concerns.

- Distinct mechanisms such as downstream BRAF class I or MEK mutation (e.g., $MEK1^{P124L}$ as described by Foth et al., bioRxiv (doi:10.64898/2025.12.18.695203) that result in gain-of-function signaling that is independent of upstream oncogenic signals from RAS. These are not anticipated to be sensitive to RAS(ON) inhibition and could be appropriately described as driving “absolute resistance”.

Thus, as the referee notes, there appears to be a dosage dependence in the increase in RAS pathway signaling driven by KRAS amplification, exemplified by the parental and resistant HPAF-II models. Consistent with our hypothesis, we agree the amplification levels in the latter appear to result in some, but not complete, abrogation of pathway inhibition by daraxonrasib treatment but appear sufficient to drive tumor outgrowth on treatment and be representative of the on-treatment relapse observed in the clinical setting. Of course, the two themes above are not mutually exclusive and our results do not cover orthogonal mechanisms, e.g., epigenetic changes that could also drive daraxonrasib resistance in addition to the mechanisms explored herein.

To further elucidate the relationship between RAS protein levels and downstream signaling flux we have added Figure 3a right panel and Extended Figure 3a and expanded our findings to an engineered HuPT3 KRAS G12R mutant cell line wherein we used doxycycline induction to progressively increase KRAS G12R expression levels. In this model, consistent with the hypothesis that KRAS expression level is positively correlated with RAS pathway signal output, KRAS overexpression led to corresponding increases in ERK phosphorylation. As anticipated, we observed a rightward shift in the inhibitory potency of the RAS(ON) multi-selective inhibitor RMC-7977 with increasing expression levels of KRAS G12R. Collectively these data support the hypothesis that KRAS expression levels influence RAS pathway output and that increases in RAS pathway output reduce sensitivity to RAS(ON) multi-selective inhibitor inhibition which ultimately can drive resistance to RAS(ON) inhibition.

Finally, we draw the referee’s attention to the patient-level case in Figure 2a, where an increase in mutant KRAS copy number was detected one treatment cycle prior to radiographic relapse. This temporal relationship, while anecdotal, aligns with the mechanistic evidence from our engineered models and supports the translational relevance of KRAS amplification as a driver of acquired resistance to daraxonrasib.

Together, these additions strengthen the causal link between KRAS amplification and reduced daraxonrasib response and integrate preclinical functional validation with patient-level observations.

Figures Incorporated Into the Manuscript (Figures 3a and c right panels and Extended Data Figure 3a, respectively.)

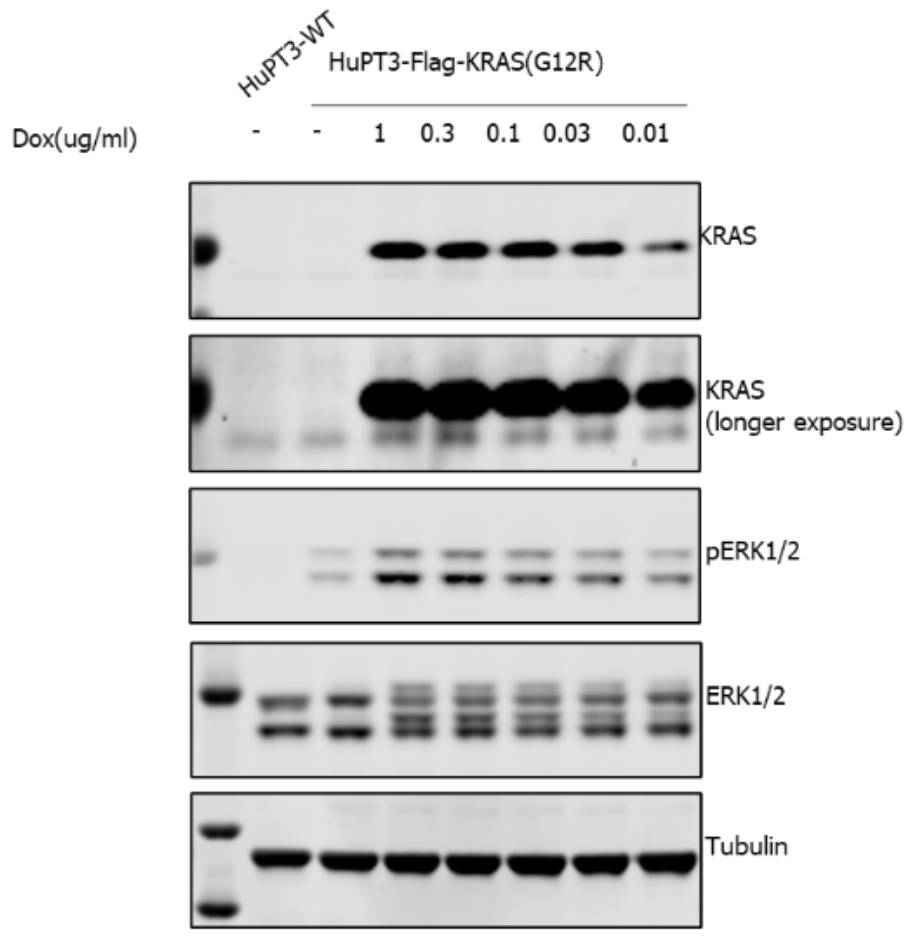

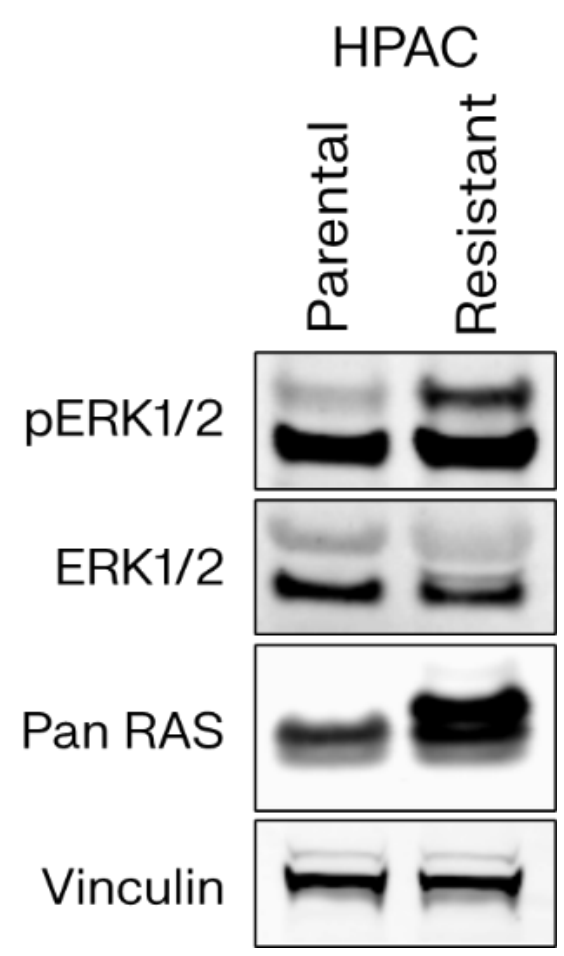

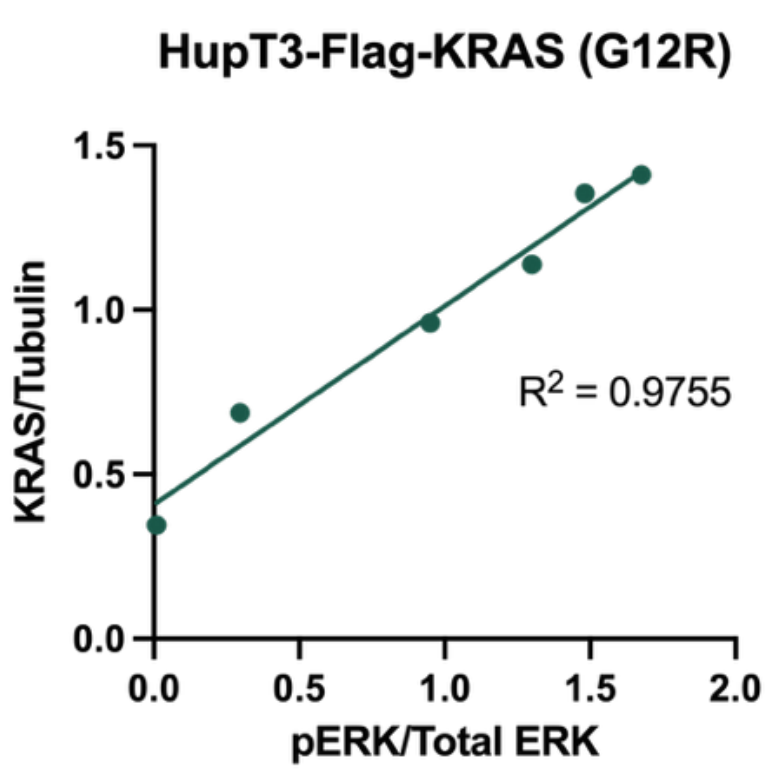

Additional Figures for Referees Consideration Only

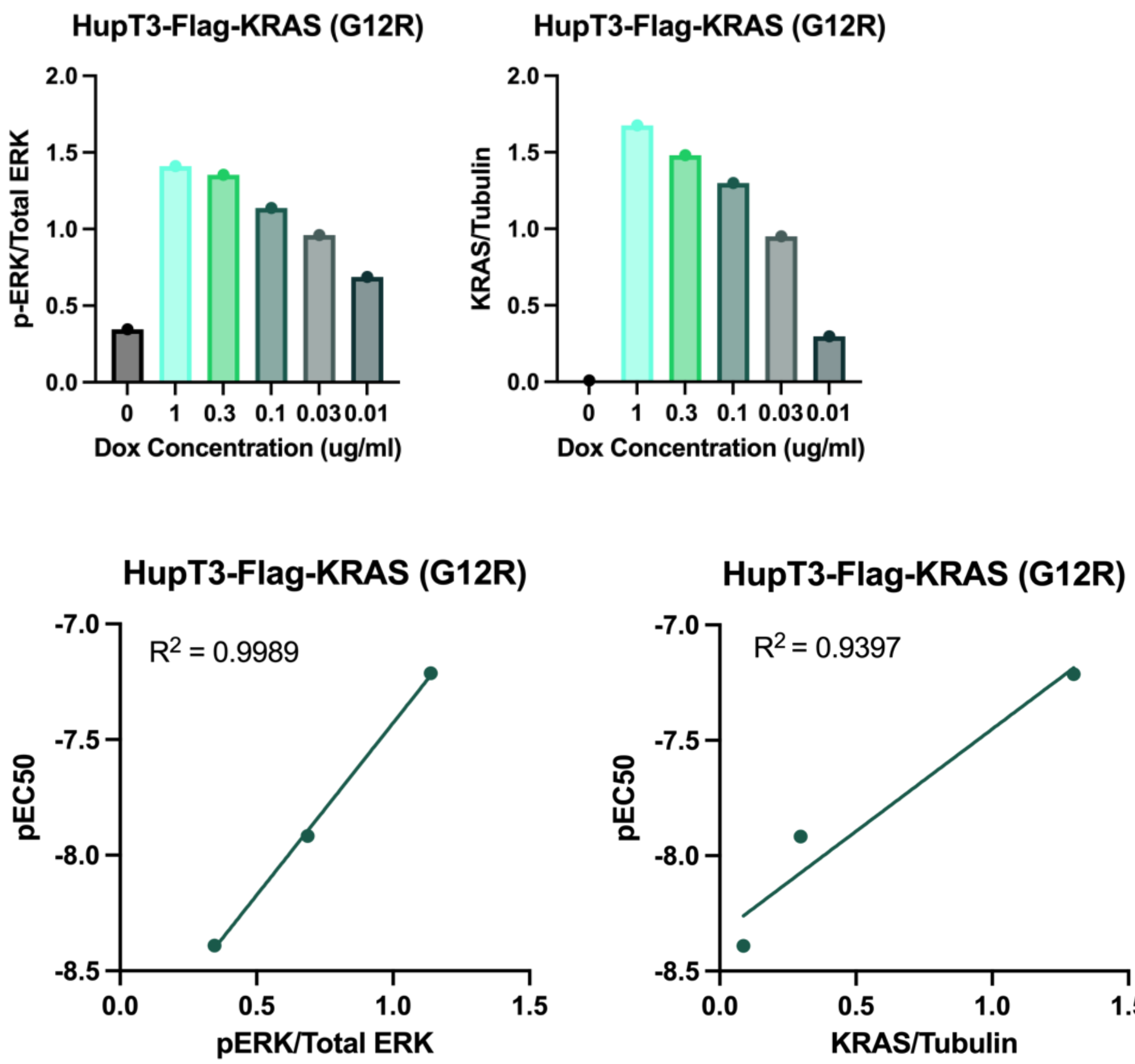

R4-7

Considering the heterogeneity in resistance mechanisms observed in patients, some degree of intra-model heterogeneity could be expected among CDX mice; have the authors replicated resistance patterns across experiments? Since resistance appears multifactorial, variability between animals is plausible.

We thank the referee for requesting this information. We have now added baseline molecular features of the HPAF-II model and the KPC/Y GEMM, including key genomic alterations and amplification status (Extended Table 5).

Intra-model resistance patterns within each CDX model were typically consistent; we speculate this is because heterogeneity in established cell lines is relatively low and it appears that a given model will tend to escape specific treatment pressures by the same set of mechanisms each time. However, as the referee notes and consistent with the heterogeneity in resistance mechanisms observed in patients, we did observe multiple putative resistance features within individual resistance models (Extended table 5).

R4-8

Moreover, to demonstrate the therapeutic relevance of drug combinations, sequential experiments (RAS inhibitor monotherapy followed by combination at regrowth) would be particularly informative. Clarification on whether coexisting alterations occur within the same clones or across subclones (e.g., via single-cell analyses) is also of interest.

We agree with the referee regarding the value of sequential experiments. For example, Figures 5g and h represent this scenario in which HPAF-II tumors that relapsed under monotherapy were passaged while being maintained on monotherapy to establish resistance. Following regrowth under continued treatment pressure, the combination treatment was initiated. This design reflects a sequential monotherapy-to-combination experiment at regrowth.

Similarly, we have now added more context for a sequential experimental paradigm with a daraxonrasib + T-Dxd combination and elaborated on the implications (below and Figure #):

The top panel shows the antitumor effects in Capan-1 parental tumors wherein the combination of daraxonrasib with T-Dxd shows marked durability whereas tumor outgrowth is observed in the context of daraxon monotherapy. Upon relapse under daraxonrasib monotherapy, resistant tumors were passaged and maintained under continued treatment to establish a resistant model. Sequential combination treatment was then tested this in this daraxonrasib-resistant Capan-1 tumor model (Lower panel).

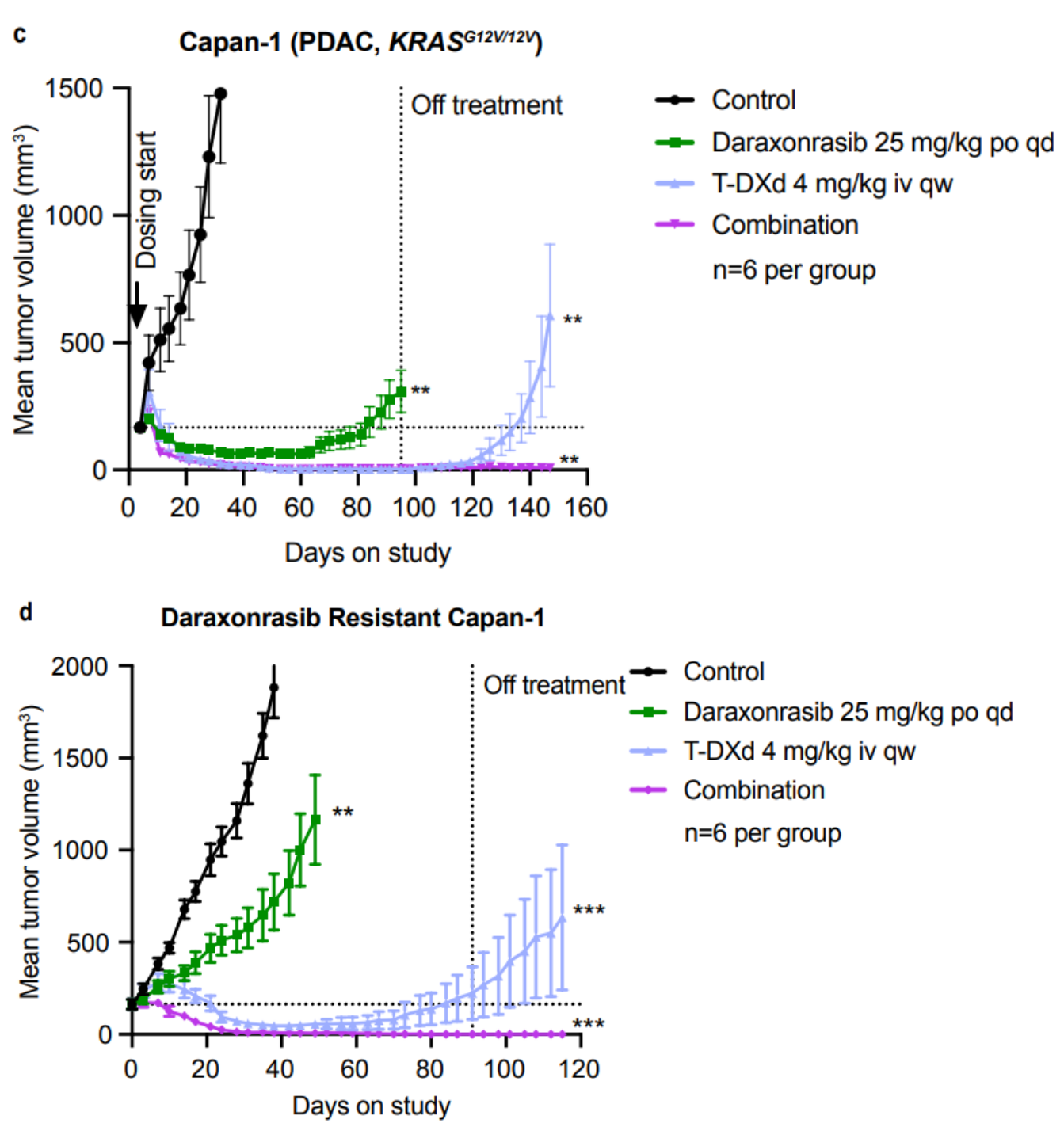

Given the complexity of these experiments, we have now added a schematic (R2-5 and Supplementary Methods) to clearly illustrate the development of the acquired resistance models and the subsequent combination testing strategies. The resistant models were generated through sequential in vivo

monotherapy (monotherapy → tumor regression → regrowth/relapse → continued monotherapy to establish a resistant model). Combination therapy was initiated at regrowth during ongoing monotherapy, representing a sequential monotherapy-to-combination setting.

4. Combination strategies

R4-9

In tumors with TP53 loss or genomic instability, chemotherapy could mimic the effect of DDR inhibitors by inducing DNA damage. Thus, combinations with platinum compounds or consideration of HRD alterations (e.g. ATM acquired mutations) merit discussion.

We appreciate the referee's comment and agree that chemotherapy, particularly platinum-based agents, can functionally overlap with DDR-targeting approaches in the setting of TP53 loss and genomic instability. We note also that Revolution Medicines is already exploring this concept clinically with our RAS(ON) inhibitors: ongoing trials are evaluating daraxonrasib in combination with standard-of-care chemotherapy regimens in PDAC. These studies are designed, in part, to assess whether cytotoxic chemotherapy may complement RAS pathway inhibition and influence resistance evolution.

We have also evaluated daraxonrasib–chemotherapy combinations in preclinical models, specifically as a potential strategy to mitigate or delay resistance mechanisms, including those associated with non-genomic features. These efforts are ongoing and form the basis of a separate, dedicated publication. We have revised the Discussion to contextualize the rationale for exploring chemotherapy combinations alongside DDR-targeting strategies.

R4-10

The T-DXd combination is compelling, but since it is an ADC, distinguishing between the cytotoxic payload effect, targeted HER2 inhibition, or both would be important. Comparative experiments with trastuzumab or HER2 TKIs could clarify this.

We appreciate the referee's insight and agree that distinguishing between HER2-targeted signaling inhibition and the cytotoxic payload effect of T-DXd is important for interpreting these results. In a prior publication (Bulle and Lim; doi.org/10.1038/s41467-024-46811-w), the authors evaluated combinations of MEK or ERK inhibition with HER2-directed agents such as afatinib and trastuzumab and compared these with the results observed with T-Dxd combination. Neither the trastuzumab nor the afatinib

combination produced the tumor regressions that were observed in combination with T-Dxd (in the same xenograft models as used in the present study with daraxonrasib), suggesting that HER2 signaling blockade alone is insufficient to drive meaningful antitumor activity in this context. Rather, it has been previously demonstrated that a key determinant of the significant combinatorial benefit is the delivery of the cytotoxic payload. In addition, the combinatorial and complementary effect of RAS pathway inhibition along with cytotoxic agents in preclinical models of PDAC has also been demonstrated by Dilly et al., and Singhal et al. (doi.org/10.1158/2159-8290.CD-24-0177; doi.org/10.1038/s41591-024-02903-0).

Finally, we have (now) also tested this combination hypothesis in the KP-4 model, which exhibits increased RAS pathway flux via upregulated RTK signaling levels at baseline and is relatively unresponsive to daraxonrasib or T-Dxd monotherapy. These data are consistent with our hypothesis that while HER2 upregulation in this model may play a role in daraxonrasib monotherapy resistance, inhibition of this RTK via an ADC alone is insufficient for antitumor activity. However, in line with the hypothesis above delineating complimentary effects of RAS inhibition and cytotoxic chemotherapy, we see a profound combinatorial effect in this model and have now included these results (below) in Figure 4.

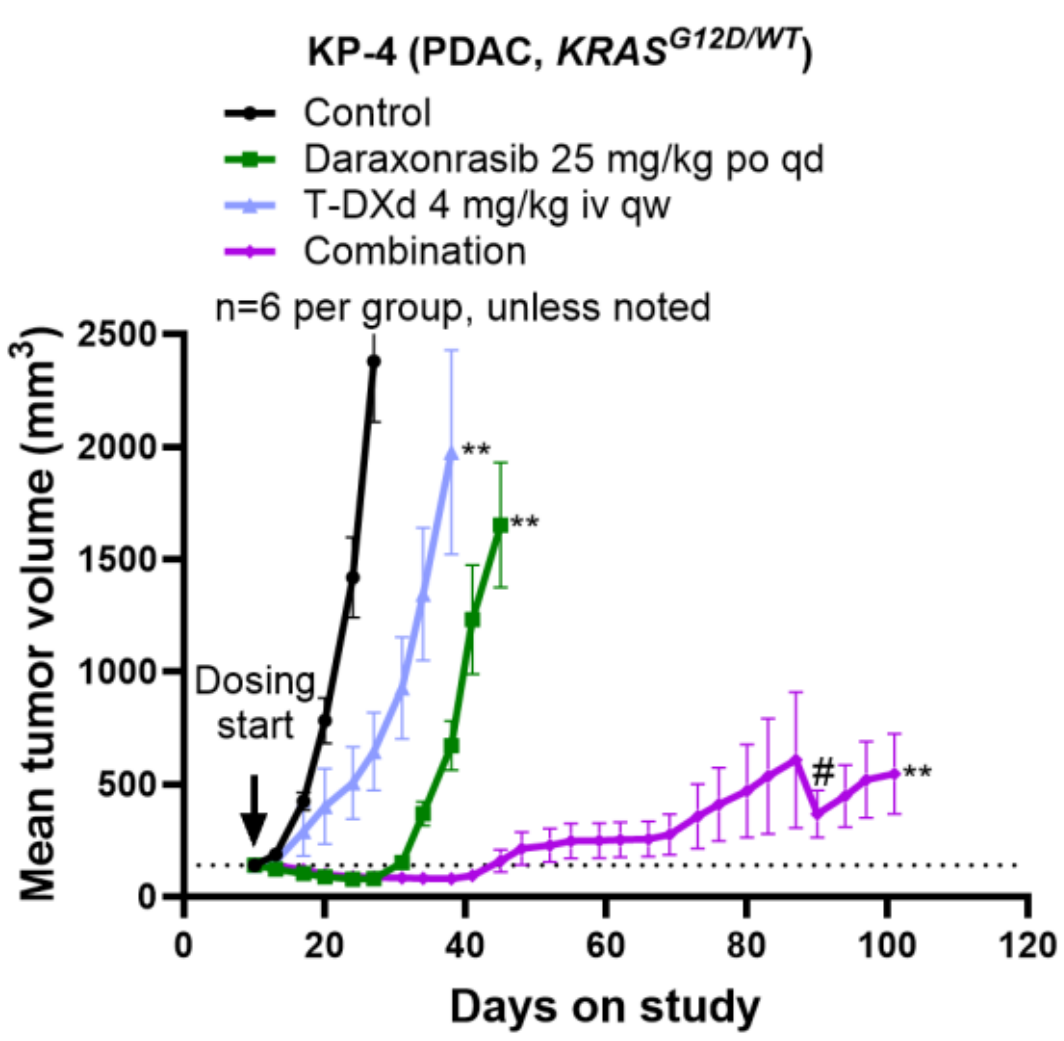

We have now added clarifying text to the manuscript to explain the overall rationale for T-Dxd combination effects and to place our findings within the context of prior mechanistic observations.

R4-11

Finally, KRAS amplification may simply reduce drug efficacy through target saturation. If so, higher doses of daraxonrasib may partially overcome this, which would be interesting to evaluate relative to dual inhibition strategies.

We appreciate and completely agree with the referee's view regarding the relationship between KRAS amplification, target coverage, and potential dose effects (see response R4-5). We agree that increasing the exposure of daraxonrasib could mitigate this (see discussion above in R4-5) however the extent to which this is possible is limited by the on-target, wild-type RAS mediated toxicities of RAS(ON) multi-selective inhibition. All our preclinical studies have used a dose of daraxonrasib, 25 mg/kg QD, that is translatable i.e. an unbound exposure in mice that is equivalent to that in humans at the dose (300 mg, QD) used in our pivotal monotherapy study in patients with mPDAC. We have added clarifying text to the Discussion to reflect this reasoning.

Additional comments

R4-12

There are inconsistencies in the Extended Table numbering: Tables 1 and 5 appear identical, while Ext Table 1 itself is missing. Please verify and correct the numbering throughout.

We appreciate the referee's careful attention to the Extended Table formatting. We identified the inconsistencies noted—specifically, the duplication between Extended Tables 1 and 5 and the missing reference to Extended Table 1—and have now corrected the numbering and cross-references throughout the manuscript. All Extended Tables are now accurately labeled and consistently cited.

R4-13

Provide exact sample sizes per group in all panels.

We appreciate the referee's comment and agree that providing exact sample sizes improves clarity and transparency. We have now added the precise n for each group in all relevant figure panels and updated the corresponding figure legends accordingly.

R4-14

Figure 1: Include KRAS copy number data.

To aid in interpretating KRAS copy numbers for patients shown in Figure 1 and 2, while keeping the oncoprint and other figures visually easy to interpret, we have added references in the Figure 1 legend to where the KRAS copy numbers can be found in Extended Data Table 3. As mentioned in other comments, we have also now re-ordered patients in Extended Data Tables in numerical order to aid in referencing the tables.

R4-15

Figure 3a and 3d: Clarify whether genuine pERK/ERK

We appreciate the referee's question regarding whether true pERK/ERK hyperactivation occurs in the resistance models. We have added language to clarify this in the figure legend. Additionally, consistent with response to R4-6, we include additional pERK/ERK quantification data for the HuPT3 and HPAC models for referee's reference. These data further clarify the extent and context of ERK pathway changes in the setting of emerging resistance.

R4-16

Extended Tables 1–3: ; clarify patient ordering and color coding in table

We appreciate the referee's comment and agree that clearer presentation of the tables will improve referencing figures with the tables. We have now standardized patient ordering numerically and removed color-coding as it was just used as formatting instead of scientific significance.

R4-17

Ext Table 2 : complete with baseline genomic data

We appreciate the referee's comment and agree with the recommendation. We have now completed the tables with the full baseline genomic data for all patients, consistent with the information already provided for the higher-dose cohorts.

R4-18

Ext Table 3 : clarify patient ordering and color coding; add the co alterations.

As above, we have now standardized patient ordering in numerical order across tables. We removed table coloring which was just formatting, as it had no scientific significance. Co-alterations for the patients in Extended Data Table 3 are found in Extended Data Table 4, to keep Table 3 focused on acquired KRAS amplification.

R4-19

Ext Table 4 : same point about patient ordering, indicate whether blank rows (4 patients #29, 5, 38, 57) reflect missing data or no alteration detected.

We have now standardized patient ordering in Extended Data Table 4 numerically and have filled in blank cells indicating “not detected”.

R4-20

Comment on the relative sensitivity of ctDNA vs tissue testing (e.g. for KRAS mutation detection).

We appreciate the referee’s request for commentary on the relative sensitivity of ctDNA versus tissue testing. In general, tissue-based NGS remains the most sensitive approach for detecting tumor mutations and CNVs. Tumor biopsies also offer ability to characterize non-genomic mechanisms of resistance, spatial differences, and intra-tumoral heterogeneity. However, due to the difficulty of obtaining biopsies at progression, particularly in PDAC where progression and a patient’s health decline can be rapid, large-scale clinical studies of acquired resistance typically utilize ctDNA (referenced in the manuscript). The Guardant Health Infinity assay used in this study is however one of most well characterized and sensitive assays available for mutation detection with an LOD of SNVs of about 0.3% VAF (https://guardanthealth.com/wp-content/uploads/AACR-2023-Infinity-RUO-AV-Poster.pdf).

As highlighted in the response to previous comments, we have attempted to control for false negatives in alteration detection at resistance by requiring that at least one somatic alteration was detected in pre-treatment ctDNA of all patients included in this study. We have added a brief sentence about sensitivity limitations of ctDNA, in the paragraph in the discussion where we discuss the value of tissue analysis for future studies.

R4-21

Author contributions are very generic; please specify individual responsibilities

We appreciate the referee's comment and agree that providing greater specificity regarding author contributions will strengthen transparency. We have now prepared a detailed spreadsheet outlining the individual responsibilities and contributions of each author, and this information has been incorporated into the revised Author Contributions section.

700 Saginaw Drive
Redwood City, CA 94063
